# Transcriptomic profiling of skeletal muscle in the *DMD*^*mdx*^ rat model of Duchenne muscular dystrophy

**DOI:** 10.1038/s41598-025-14756-9

**Published:** 2025-08-11

**Authors:** Abdolvahab Ebrahimpour Gorji, Katarzyna Kliczkowska, Marcin Ollik, Caroline Le Guiner, Jacek Wilczak, Wojciech Bielecki, Piotr Ostaszewski, Masoud Shirali, Zahra Roudbari, Tomasz Sadkowski

**Affiliations:** 1 Department of Physiological Sciences, Institute of Veterinary Medicine, Warsaw University of Life Sciences, Warsaw, 02-776 Poland; 2 Department of Pathology and Veterinary Diagnostics, Institute of Veterinary Medicine, Warsaw University of Life Sciences, Warsaw, 02-776 Poland; 3 Department of Biometry, Institute of Agriculture, Warsaw University of Life Sciences, Warsaw, 02-776 Poland; 4 Translational Gene Therapy Laboratory (TaRGeT), Nantes Université, CHU de Nantes, INSERM UMR 1089, Nantes, France; 5 School of Biological Sciences, Queen’s University Belfast, Belfast, BT9 5DL UK; 6 Department of Animal Science, Faculty of Agriculture, University of Jiroft, Jiroft, Iran

**Keywords:** Duchenne muscular dystrophy (DMD), Dystrophin gene mutation, Skeletal muscle degeneration, RNA-seq gene expression analysis, MAPK signaling pathway, *DMD*^*mdx*^ rat model, Transcriptomics, Neuromuscular disease

## Abstract

Duchenne muscular dystrophy (DMD) is a severe X-linked recessive disorder caused by a mutation in the *Dmd* gene, leading to progressive muscle degradation, increasing weakness, and typically resulting in death before the third decade of life. To investigate the pathobiology of DMD, this study employed the Sprague-Dawley *Dmd*-mutated rat model (*DMD*^*mdx*^) and analyzed gene expression profiles and pathological molecular pathways. The methods used included histopathological, biochemical, and transcriptomic analyses of dystrophic skeletal muscle from *DMD*^*mdx*^ and wild-type (WT) individuals. Histological analysis of skeletal muscle tissue from *DMD*^*mdx*^ rats revealed multifocal necrosis, fibrosis, and inflammation, whereas WT rats displayed normal muscle architecture. Biochemical analysis revealed significant alterations in plasma markers of muscle damage and metabolism in *DMD*^*mdx*^ rats compared to WT controls, including elevated AST, ALT, ALP, CPK, and LDH levels. Additionally, oxidative status measurements showed reduced antioxidant capacity and increased lipid peroxidation in dystrophic skeletal muscle, as evidenced by lower TAS, GR, GPx, and SOD activities and higher TBARS levels. RNA-seq analysis identified 3,615 differentially expressed genes between the two groups, associated with muscle contraction, extracellular matrix (ECM) organization, and cytoskeleton organization. Notably, *Dmd*, *Actc1*, *Col6a1*, and *Mmp2* were significantly downregulated. Gene ontology and pathway enrichment analyses indicated dystrophic changes in skeletal muscle, disruptions in calcium homeostasis, and alterations in actin cytoskeleton regulation. KEGG and Reactome pathway analyses revealed upregulation of the MAPK signaling and immune system pathways and downregulation of the ECM organization pathway. These findings support the hypothesis that targeting complex intracellular signaling pathways in DMD may represent a promising therapeutic strategy. Given that the *DMD*^*mdx*^ rat model closely mimics human DMD pathology compared to other animal models, it offers a more realistic platform for studying the molecular mechanisms of the disease and improving the translational potential of therapeutic approaches.

**Supplementary Information:**

The online version contains supplementary material available at 10.1038/s41598-025-14756-9.

## Introduction

DMD is a severe, progressive X-linked recessive disease due to mutations in the dystrophin gene (*Dmd*) that predominantly disrupts skeletal muscle tissue. It encodes the protein dystrophin, which is highly important for structural integrity in muscle fibers by its subsarcolemmal connection between the actin cytoskeleton and the extracellular matrix. The absence of dystrophin leads to repeated muscle damage, poor regenerative capacity, progressive muscle degeneration, and early mortality^1^. One of the worst-hit tissues in DMD patients is the skeletal muscle, manifested as muscle wasting, weakness, and fibrosis. Animal models, the *mdx* mouse models, the DMD dog models, the DMD pig models, and the DMD rat models have thus far been highly instrumental in the study of the pathogenesis of DMD and afford insight into the progress of the disease and possible therapeutic intervention^2–4^.

Of them all, the rat models for DMD are beneficial due to their larger size and physiological similarities to human muscle pathology^5–7^. Such models help in realizing the histopathological changes and the molecular disruptions underlying the disease mechanism in DMD. For instance, dystrophin deficiency in the models closely resembles that in human patients, resulting in degeneration, inflammation, and fibrosis of skeletal muscles, just like in humans^8^. This again points to a better resemblance of rats to many features of DMD in humans, including more severe muscle damage, more evident cardiomyopathy, more progressive muscle degeneration, and more severe deficits in motor responses^5,7^. The DMD symptoms are not as severe in *mdx* mice, with good muscle recovery due to the high activation of satellite cells^9–11^.

A study by Teramoto et al. (2020) described a new rat model harboring in-frame dystrophin gene mutations that mimicked the dystrophic phenotype of Becker muscular dystrophy (BMD). Though this study was mainly associated with BMD, it also gave insight into dystrophin expression patterns and skeletal muscle pathology relevant to the pathogenesis of DMD. Thus, in this model, progressive muscular dystrophy due to the functional absence of the complete dystrophin protein resulted in remarkably distinctive gene expression changes as a starting point in exploring disrupted molecular pathways in DMD^12,13^. In human models, gene expression profiling has also identified vital information on the biological processes and pathways disrupted by the absence of dystrophin, which includes inflammation, calcium signaling, and metabolic pathways^14^.

Next-generation sequencing (NGS) technologies aim to perform detailed and large-scale analyses, allowing researchers to study the effects of gene mutations on biological processes and molecular pathways^15^. A key example is RNA-Seq, which has significantly advanced our understanding of DMD gene expression and related pathway disruptions^16^. Transcriptomic data analyses allow the identification of differentially expressed genes (DEGs) associated with disease progression and pathway enrichments, highlighting changes in critical signaling processes such as inflammatory response, muscle regeneration, and calcium homeostasis^1^. This molecular insight is essential for establishing therapeutic targets and testing new treatments to restore dystrophin expression, including gene replacement, antisense oligonucleotides, and gene editing strategies, and/or to compensate for dystrophin deficiency by using pharmacological approaches^17^.

Despite progress in elucidating the pathology of DMD, critical gaps remain in completing the entire network of molecular pathways affected by dystrophin deficiency. Most previous studies used *mdx* mouse models and mainly concentrated on selected pathways^18^. The *DMD*^*mdx*^ rat model presents the opportunity to bridge this gap with a much more robust system to pursue pathways for therapeutic implications^5^.

Accordingly, the present study has developed a comprehensive gene expression and pathway enrichment analysis in the *DMD*^*mdx*^ rat model. We implement next-generation sequencing capabilities to probe into the molecular landscape of DMD by identifying essential dysregulated genes and pathways at distinct stages of disease progression. Our research represents another step towards filling the gap in understanding the molecular mechanisms of DMD pathology, within the framework of a new *DMD*^*mdx*^ rat model of significant translational importance due to its greater similarity to human DMD. This will provide basic information on the transcriptome profile of skeletal muscles (*biceps femoris* muscle), which will enable the planning of future studies focused on exploring specific genes and signaling pathways identified in this study, as well as the planning of future therapeutic interventions targeting the molecular factors causing DMD, with an emphasis on therapeutic strategies, gene editing technologies, and personalized medicine approaches.

## Materials and methods

### Animals and sampling

The Sprague-Dawley (*Rattus norvegicus*) male dystrophin-deficient rats (strain name: *DMD*^*mdx*^) and wild-type (WT) healthy rats were obtained from Nantes Université and the Nantes Vet School-ONIRIS, Nantes, France^5^. *DMD*^*mdx*^ and WT rats were littermates. All experimental protocols were approved by the 2nd Local Ethical Committee for Animal Experiments, number WAW2/128/2022. All experiments were performed in accordance with relevant guidelines and regulations and reported in accordance with ARRIVE guidelines (https://arriveguidelines.org). We enrolled rats aged 13–14 weeks (around 4 months of age) into two groups: *DMD*^*mdx*^ (*n* = 7), exhibiting a phenotype similar to DMD disease, and WT rats (*n* = 7). The mean weight of the rats was 470.86 ± 38.22 g and 554.00 ± 49.83 g for *DMD*^*mdx*^ and WT, respectively. Rats were housed in the animal house of the Institute of Veterinary Medicine, Warsaw University of Life Sciences, Poland, and kept in plastic cages (Tecniplast S. p. A, 21 020, Italy) in a temperature-controlled room (22 °C ± 1 °C) on a 12 h light/dark cycle, with 50% humidity. Animals were fed the Labofeed B maintenance rat chow (Wytwórnia Pasz Morawski, Poland) ad libitum with free access to water. After 12 h of fasting, animals were anesthetized with an intraperitoneal injection of ketamine/xylazine (90/9 mg/kg) and euthanized by exsanguination via cardiac puncture. Euthanasia was completed by anesthetic overdose, in accordance with Directive 2010/63/EU, Annex IV. The blood was collected by heart puncture using the Sarstedt blood collection system with lithium heparin (Sarstedt AG & Co. KG, Nümbrecht, Germany). Samples were immediately centrifuged to obtain plasma. The right and left hind limb skeletal muscle tissue (*biceps femoris*) was collected. Samples were quickly cleaned of connective tissue, placed in liquid nitrogen, and stored at − 80 °C until analyzed. Samples for histopathological examination were placed in 10% buffered formalin until analyzed.

### Histopathological examination

For histopathological examination, *biceps femoris* muscle samples (*n* = 7 per genotype) were fixed in 10% buffered formalin, embedded in paraffin wax, cut in 4 μm sections, and stained with hematoxylin and eosin (H&E). Additional slides were stained with Picrosirius red (PSR) stain for collagen and Koss stain for calcium. Histopathological slides were evaluated with light microscopy using the Leica DM 1000 microscope (Leica Microsystems GmbH, Germany) and KoPa Capture Pro 9.0 software (https://ostec.com.cn/; Guangzhou Ostec Electronic Technology Co., Ltd., China). The size of the fibers was determined in slides stained with Picrosirius red by measuring a minimum of 250 myofibers from each rat in the randomly selected fields using 100x magnification and the above software. Myofibers were measured perpendicular to the axis of the muscle fiber – maximal width and minimal width (max diameter and min diameter, respectively), as was previously described^19^. Minimal Feret’s diameter was not measured, as maximal and minimal diameters perpendicular to the fiber axis adequately captured fiber size variation. The fibrous connective tissue and skeletal muscle tissue areas were calculated using MicroImage 4.0 software (Olympus Optical CO., GMBH, USA). Fifteen pictures from each rat were taken from representative areas, avoiding superficial areas covered with perimysium from 200x magnification and Picrosirius red staining. Two pathologists independently performed the analyses, each evaluating all samples from all rats.

### Biochemical analyses

Biochemical analyses (*n* = 7 per genotype), including aspartate aminotransferase (AST, U/l), alanine aminotransferase (ALT, U/l), alkaline phosphatase (ALP, U/l), glucose (mg/dl), creatinine (mg/dl), urea (mg/dl), total protein (g/l), albumin (g/l), creatine phosphokinase (CPK, U/l), gamma-glutamyltransferase (GGT, U/l), lactate dehydrogenase (LDH, U/l), total bilirubin (mg/dl), amylase (U/l), uric acid (mg/dl), calcium (Ca, mg/dl), phosphorus (P, mg/dl), magnesium (Mg, mg/dl), potassium (K, mmol/l), and sodium (Na, mmol/l), were determined in Department of Veterinary Laboratory and Clinical Diagnostics, Institute of Veterinary Medicine, Warsaw University of Life Sciences, Poland. The analyses were performed using the Miura One automated biochemistry analyzer (I.S.E. S.r.l., Rome, Italy) and commercially available Pointe Scientific reagent kits (Pointe Scientific Inc., Canton, MI, USA) according to the manufacturer’s protocols (*n* = 7 per genotype). Blood plasma samples were processed under standardized conditions to ensure the reliability and accuracy of the results.

### Oxidative status measurements

*Biceps femoris* muscle samples (*n* = 7 per genotype) were homogenized in PBS, vortexed for 15 min, and centrifuged at 500×g for 15 min. The supernatants were used for analyses. Total antioxidant status (TAS) and activities of glutathione reductase (GR), glutathione peroxidase (GPx), and superoxide dismutase (SOD) activities were measured in the homogenates using Randox assay kits (TAS: NX2332, GR: GR2368, GPx: RS504/505/506, SOD: SD125) following the manufacturer’s protocols (Randox Laboratories Ltd., UK). Thiobarbituric acid reactive substances (TBARSs) were analyzed per Aguilar Diaz’s method^20^.

### RNA extraction, validation, and sequencing

According to the manufacturer’s protocol, total RNA was extracted from *biceps femoris* muscle samples (*n* = 4 per genotype; individuals most representative of the group average were selected) using the Rneasy Mini Kit (Qiagen, USA). RNase-free DNase was implemented to eliminate any DNA contamination. Quantifying the isolated RNA and libraries was performed on Qubit (Thermo Fisher Scientific, USA), and profiles of RNA and libraries were measured on Tape Station D1000 (Agilent Technologies, USA), generating a quality control (QC) report. The VAHTS Universal v10 RNA-seq Library prep kit for Illumina (Vazyme, China) was used. Sequencing was conducted on Illumina’s NovaSeqX Plus platform with a 25B flowcell (Illumina, USA), generating 100 million PE150 reads. Raw reads were obtained in a FASTQ file format. The files have been checked to assess the sequencing quality according to the Q score. RNA isolation, library preparation, and sequencing were performed at Dante Labs (Dante Labs, L’Aquila, Italy).

### RNA-seq data validation and processing

Following RNA-Seq sequencing, the next step was quality control, entailing adapter trimming, removing sequences not from the rat genome, and removing low-quality reads and uncalled bases. Since the data obtained came from Illumina, we utilized FastQC to assess the quality of the files. FastQC is freely available under the URL (http://www.bioinformatics.babraham.ac.uk/projects/fastqc/). Following quality control, we evaluated the mapping of the rat genome. Mapping extensive high-throughput sequencing data against a known genome is essential to RNA-Seq data analysis. In the present work, we used an Ensembl genome version’s Gene Transfer Format (GTF) file for the *Rattus norvegicus* genome as a reference. Paired-end reads were aligned using Hierarchical Indexing for Spliced Transcripts 2 (HISAT2)^21^, a software optimized for rapid and efficient mapping. Then, we used the featureCounts package to read and quantify RNA sequencing. FeatureCounts summarizes the aligned reads in SAM format and outputs count data by gene in a matrix.

### Differentially expressed genes

Differential gene expression analysis plays a vital role in processing RNA-seq data to investigate gene expression variations across distinct *DMD*^*mdx*^ and WT experimental conditions in rat muscle samples. Analysis was performed to find upregulated or downregulated genes, followed by normalized RNA-seq quantification data. The analysis used the DESeq2 pipeline in the R programming language^22^. All plots were done in R, using functions from ggplot2, Volcano, the principal component analysis (PCA), and pheatmap packages for visualization. Several statistical approaches have been used for detecting DEGs, and the selection criterion was based on log2FC between 0.3 and − 0.3 and *P* < 0.05.

### Functional analysis

Further considerations were made regarding the Gene Ontology (GO) and pathway enrichment studies using the DAVID (https://davidbioinformatics.nih.gov/; Sherman et al., 2022) and KEGG (www.kegg.jp/kegg/kegg1.html) databases. This study divided selections into three categories, including gene sets according to biological processes (BP), cellular components (CC), and molecular functions (MF). Enrichment of specific GO terms and signaling pathways for the used gene set showed significance (*P* < 0.05) by Fisher’s exact test. Only the statistically significant results were taken into consideration. After this, the findings were visualized using an online tool for further analysis and are represented by charts available at http://www.bioinformatics.com.cn/srplot.

### Statistical analysis

Histopathological features, blood plasma biochemical parameters, and antioxidative status were compared using the Student’s *t*-test with independent variance estimation. Variables with significant differences (*p* < 0.05) are presented in the graphs. Due to the sample size, normality could not be definitively rejected, justifying the use of the *t*-test. However, most variables exhibited heteroscedasticity, meaning that the variance increased proportionally with the mean. Therefore, the t-test for unequal variances (Welch’s t-test) was used to evaluate them. Statistical analyses were conducted using STATISTICA 13.3.

## Results

### Histopathological changes

**Fig. 1:**
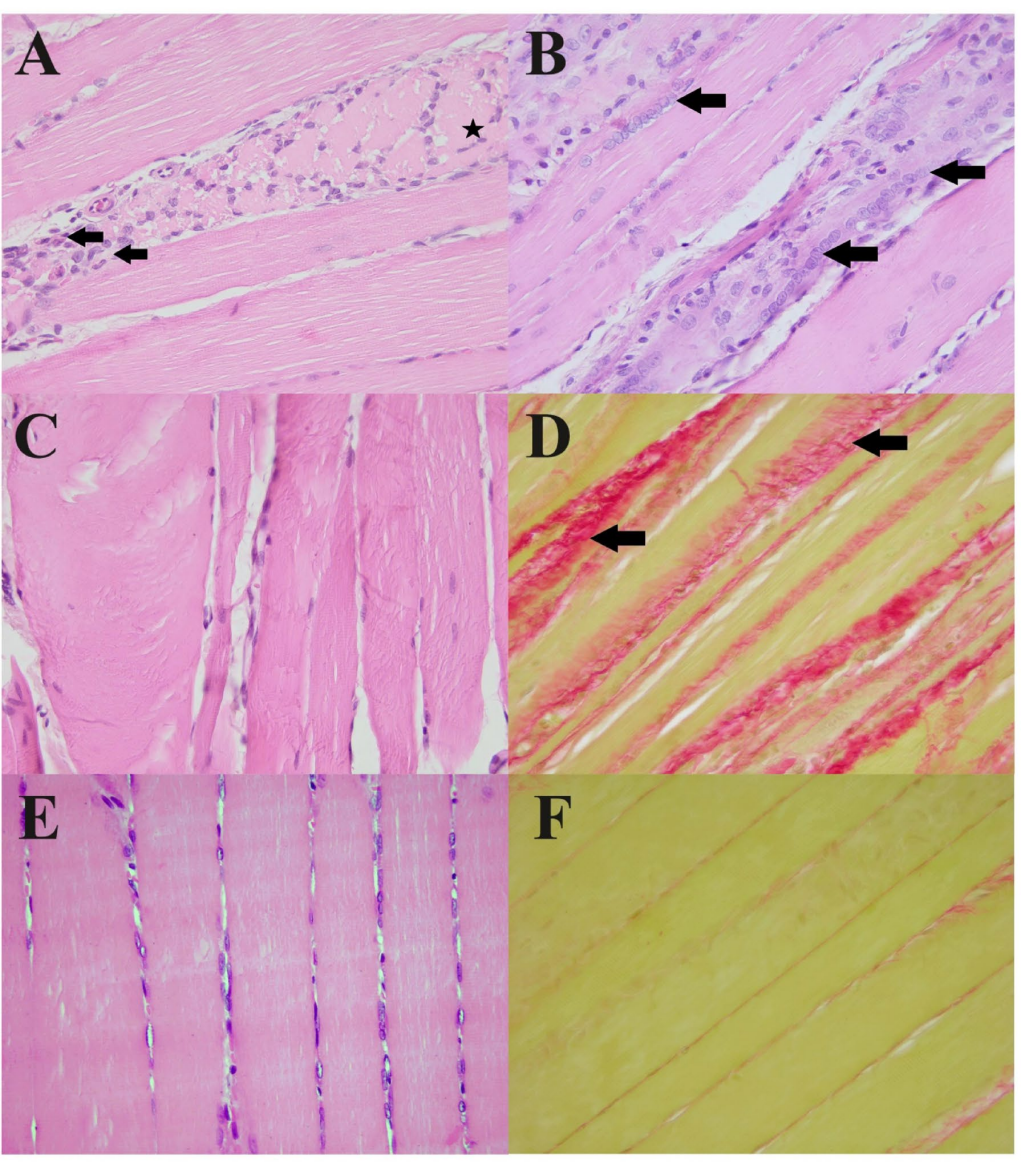
Histopathological features in the *biceps femoris* muscle of *DMD*^*mdx*^ and WT rats: **A**) multifocal necrosis of myofibers (asterisk) and inflammatory infiltrates with a predominance of mononuclear cells (arrows), *DMD*^*mdx*^, H&E stain, 400x; **B**) satellite cells at the periphery of myofibers (arrows) in *DMD*^*mdx*^ rat, indicating myofiber regeneration, H&E stain, 400x; **C**) marked variation in myofiber size, *DMD*^*mdx*^, H&E stain, 400x; **D**) interstitial hyperplasia of fibrous connective tissue (arrows) in *DMD*^*mdx*^, PSR stain, 100x; **E**) normal muscle fibers of WT, H&E stain, 100x, **F**) normal wild-type muscle of WT, PSR stain, 100x. *DMD*^*mdx*^ – Sprague-Dawley *DMD*^*mdx*^ rats; WT – Sprague-Dawley wild-type rats; H&E – hematoxylin and eosin; PSR – Picrosirius red.

Histopathological examination of *biceps femoris* muscle samples collected at 4 months of age from the *DMD*^*mdx*^ rats revealed massive multifocal myofiber necrosis, multifocal inflammatory infiltrates with a predominance of mononuclear cells, and marked interstitial hyperplasia of fibrous connective tissue (Fig. 1A-B). None of these changes were found in the WT rats. Moreover, regenerative activity was present in the form of satellite cells arranged at the periphery of the myofibers of *DMD*^*mdx*^ rats (Fig. 1C). Marked variation in myofiber size due to large hypertrophic fibers was also noted (Fig. 1D), all compared to normal WT skeletal muscle (Fig. 1E-F). Calcification wasn’t found in either WT or *DMD*^*mdx*^ rat *biceps femoris* samples.

**Fig. 2:**
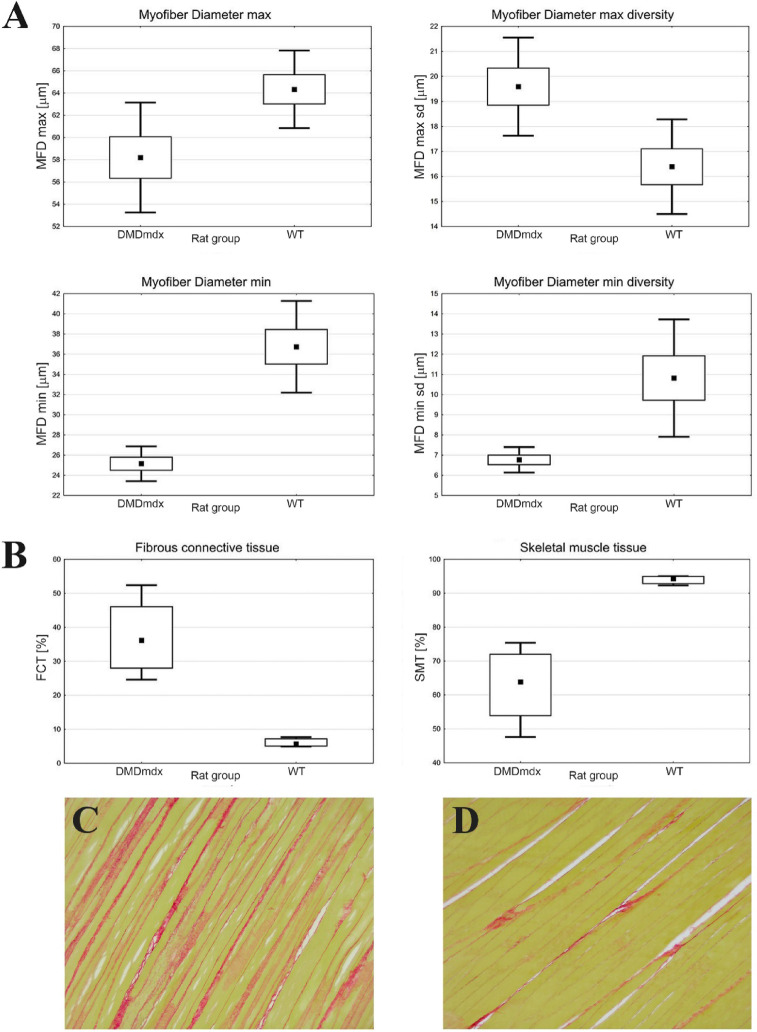
Biceps femoris muscle fiber diameter (µm) (**A**) and the average fibrous connective tissue and muscle fiber area in WT and *DMD*^*mdx*^ rats (percentage) (**B**). *DMD*^*mdx*^ muscle structure, PSR stain, 40x (**C**) and WT muscle structure, PSR stain, 40x (**D**). *DMD*^*mdx*^ – Sprague-Dawley *DMD*^*mdx*^ rats; WT – Sprague-Dawley wild-type rats; PSR – Picrosirius red. The dots represent the mean; the boxes – the standard error; the whiskers – the standard deviation. All comparisons between DMD^*mdx*^ and WT rats are statistically significant (*p* < 0.05; *n* = 7 per genotype).

The *biceps femoris* muscle fibers diameter analysis in both rat groups showed a statistically significant difference in the max diameter mean value in the *DMD*^*mdx*^ group (58.20 ± 4.95 μm) compared to the WT group (64.34 ± 3.48 μm) (*p* = 0.022). Similarly, the minimum diameter mean value is statistically different, with 25.15 ± 1.72 μm and 36.74 ± 4.54 μm (*p* = 0.000), for *DMD*^*mdx*^ and WT, respectively (Fig. 2A).

Surface area analysis of *biceps femoris* showed statistically significant differences between connective tissue surface area in *DMD*^*mdx*^ versus WT rats, which were 44.29% and 3.97% (*p* = 0.000), respectively. A similar analysis for muscle tissue in the same groups revealed 55.71% for *DMD*^*mdx*^ rat skeletal muscle tissue average area and 96.03% in WT rats (*p* = 0.000) (Fig. 2B-D).

### Blood plasma biochemical parameters

**Fig. 3:**
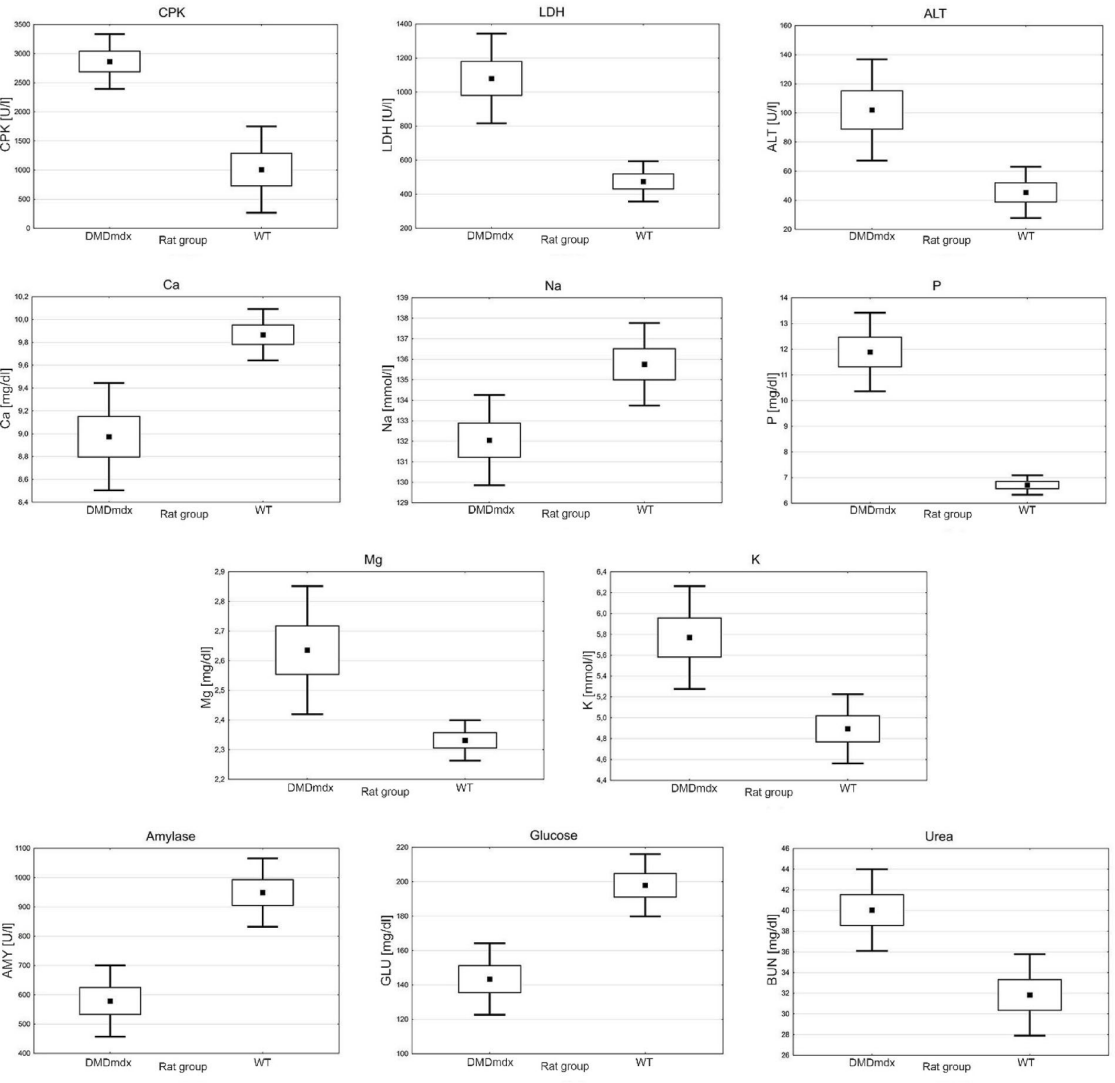
The biochemical analysis of blood plasma. CPK – creatine phosphokinase; LDH – lactate dehydrogenase; ALT – alanine aminotransferase; *DMD*^*mdx*^ – Sprague-Dawley *DMD*^*mdx*^ rats; WT – Sprague-Dawley wild-type rats. The dots represent the mean; the boxes – the standard error; the whiskers – the standard deviation. All comparisons between *DMD*^*mdx*^ and WT rats are statistically significant (*p* < 0.05; *n* = 7 per genotype).

The biochemical analysis of blood plasma showed statistically significantly lower activity in *DMD*^*mdx*^ compared to WT rats at 4 months of age in the following enzymes, respectively: mean CPK activity 2864.46 U/l and 1008.83 U/l (*p* = 0.000); mean LDH activity 1080.43 U/l and 475.03 U/l (*p* = 0.000); mean ALT activity 102.06 U/l and 45.40 U/l (*p* = 0.004) (Fig. 3). In addition, a difference in amylase activity was also demonstrated at 578.76 U/l and 948.87 U/l, showing higher activity in WT rats (*p* = 0.000). The analysis also revealed the electrolytes and minerals level variations between both examined groups: Ca (8.97 mg/l and 9.87 mg/l; *p* = 0.002) and Na (132.06 mmol/l and 135.76 mmol/l; *p* = 0.007) levels were higher in WT than in *DMD*^*mdx*^ rats. Conversely, P (11.89 mg/l and 6.71 mg/l; *p* = 0.000), Mg (2.64 mg/l and 2.33 mg/l; *p* = 0.009), and K (5.77 mmol/l and 4.89 mmol/l; *p* = 0.003) levels were lower in WT than in *DMD*^*mdx*^ rats. Moreover, significant differences in urea and glucose levels were also noticed. Higher levels of glucose (143.43 mg/dl and 197.94 mg/dl; *p* = 0.000) and lower levels of urea (40.04 mg/dl and 31.84 mg/dl; *p* = 0.002) were present in WT blood plasma than in *DMD*^*mdx*^ rats (Fig. 3). Trends of higher total bilirubin (*p* = 0.086) and uric acid (*p* = 0.086) in *DMD*^*mdx*^ rats were noticed. A trend of higher AST (*p* = 0.081) in WT rats was noticed. The remaining parameters, ALP (*p* = 0.911), creatinine (*p* = 0.163), GGT (*p* = 0.591), total protein (*p* = 0.247), and albumin (*p* = 0.638), showed no differences between *DMD*^*mdx*^ and WT rats.

### Antioxidative status

**Fig. 4:**
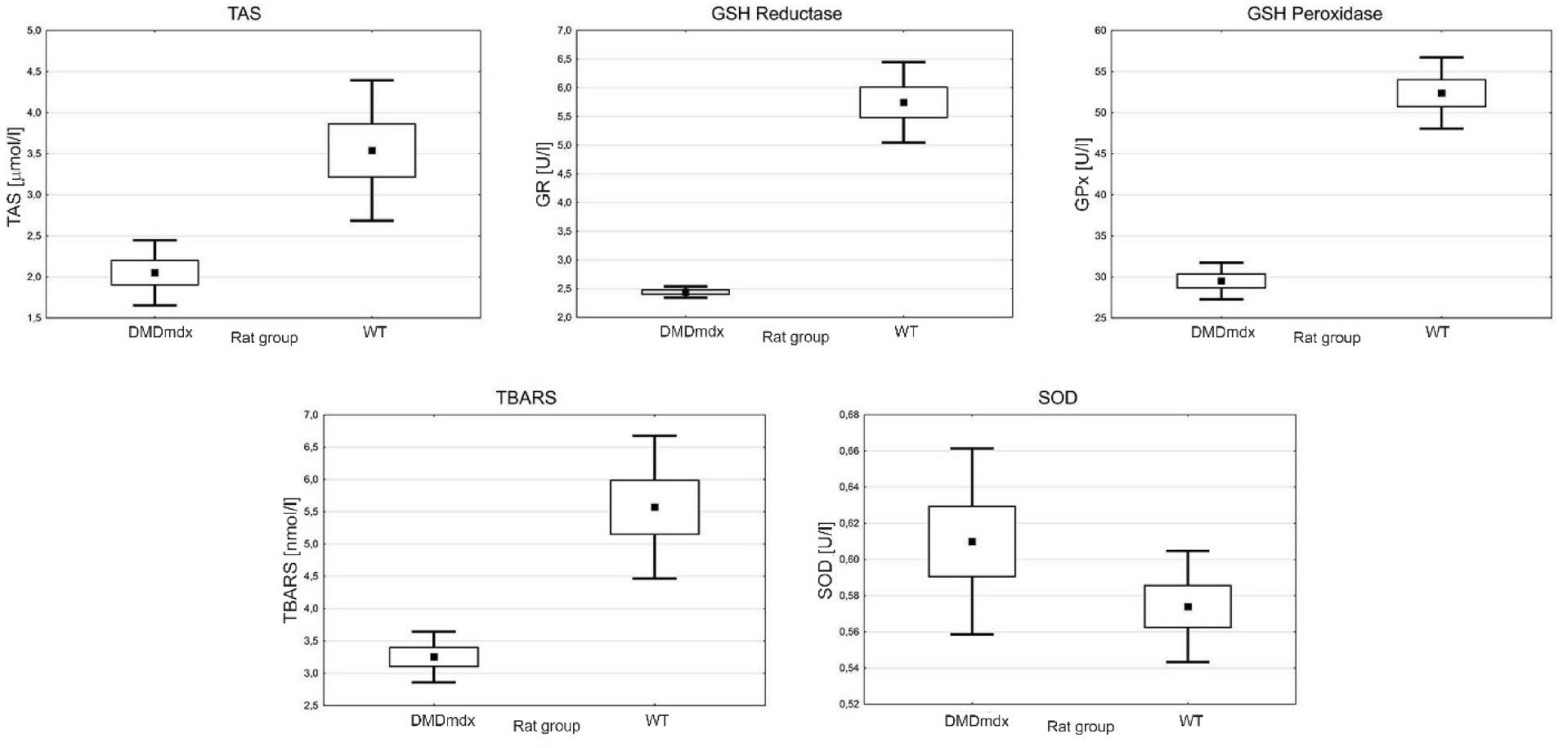
The antioxidative status parameters. TAS – total antioxidant status; GSH – glutathione; TBARS – thiobarbituric acid reactive substances; SOD – superoxide dismutase; *DMD*^*mdx*^ – Sprague-Dawley *DMD*^*mdx*^ rats; WT – Sprague-Dawley wild-type rats. The dots represent the mean; the boxes – the standard error; the whiskers – the standard deviation. All comparisons between *DMD*^*mdx*^ and WT rats are statistically significant (*p* < 0.05; *n* = 7 per genotype).

The analysis of antioxidative status parameters in the *biceps femoris* muscle homogenate showed significant differences between *DMD*^*mdx*^ and WT rats at 4 months of age. The mean TAS levels were 2.05 µmol/l in *DMD*^*mdx*^ rats and 3.54 µmol/l in WT rats (*p* = 0.003) (Fig. 4), indicating a marked reduction in total antioxidant capacity in the dystrophic group. Similarly, GSH reductase activity was significantly lower in *DMD*^*mdx*^ rats, with mean values of 2.44 U/l compared to 5.75 U/l in WT rats (*p* = 0.000). GSH peroxidase activity also showed a significant decrease in *DMD*^*mdx*^ rats, with values of 29.51 U/l vs. 52.37 U/l in WT rats (*p* = 0.000). Additionally, TBARS levels, a marker of lipid peroxidation, were significantly reduced in *DMD*^*mdx*^ rats, with mean values of 3.25 nmol/l compared to 5.57 nmol/l in WT rats (0.001). Conversely, the activity of SOD did not differ significantly between the groups, with mean values of 0.61 U/l in *DMD*^*mdx*^ rats and 0.57 U/l in WT rats (*p* = 0.144) (Fig. 4).

### RNA-sequencing data processing

After removing low-quality reads from raw RNA-Seq data of the transcriptome in the *biceps femoris* muscle of *DMD*^*mdx*^ and WT rats, the remaining reads were matched with the reference genome using HISAT2. Later, > 90–95% mapping rate of the reads to our dataset was obtained. Among those clean reads, more than 80% showed unique mapping in various regions of the rat genome. For further analysis, the non-mapped reads and the reads mapped to more than one position were discarded (Table S1).

### Analysis of differential gene expression

According to the DESeq2 pipeline statistical analysis results, the general distribution of DEGs was significantly skewed, with an adjusted p-value less than 0.05. Log2FC greater than 0.3 was the cutoff for fold change, while the tight threshold for the p-value was less than 0.05. The PCA explains 56.2% of the total variance (PC1) and distinguishes *DMD*^*mdx*^ and WT groups, while PC2 accounts for 13.9%, highlighting within-group variation (Fig. S1). According to PCA analysis, the clear separation of the *DMD*^*mdx*^ and WT groups indicates distinct transcriptional profiles associated with the loss of dystrophin.

**Fig. 5:**
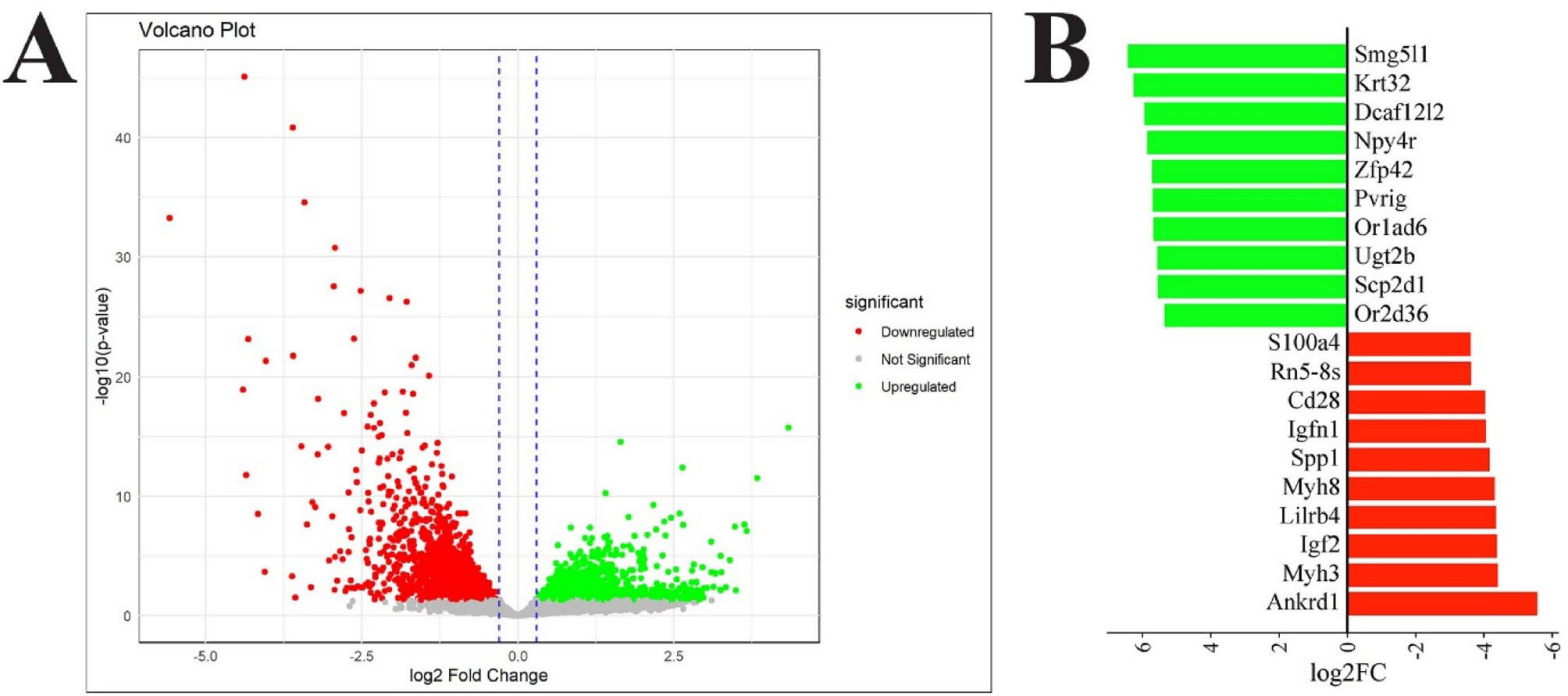
The distribution of DEGs between the *DMD*^*mdx*^ and WT rat *biceps femoris* muscle samples: **A**) The volcano plot with gene distribution. The log2 fold change (log2FC) is shown on the x-axis, while the -log10 (p-value) is shown on the y-axis. The genes on the left side are downregulated in *DMD*^*mdx*^ rats compared to the WT, while genes on the right are significantly upregulated. The vertical dashed lines represent a threshold of differential expression standard value log2FC > |0.3|. The horizontal lines represent the threshold for statistical significance (*P* < 0.05; *n* = 4 per genotype). **B**) Top 10 upregulated and downregulated genes in *DMD*^*mdx*^ rats compared to WT. Ranking according to the log2 fold change (log2FC). Significantly upregulated genes are colored green, while significantly downregulated genes are colored red. Each bar displays the level of expression change and the p-value. *DMD*^*mdx*^ – Sprague-Dawley *DMD*^*mdx*^ rats; WT – Sprague-Dawley wild-type rats; DEGs – differentially expressed genes.

The differential gene expression analysis between *DMD*^*mdx*^ and WT rat groups revealed 3,615 DEGs. Among them, 1,824 genes were upregulated, while 1,791 were downregulated in the *DMD*^*mdx*^ rats compared to the WT group (Fig. 5A; Table S2-S3). The top 10 significantly upregulated and downregulated genes are shown in Fig. 5B, ranked by log2 fold change and p-value.

### Gene ontology analysis

**Fig. 6:**
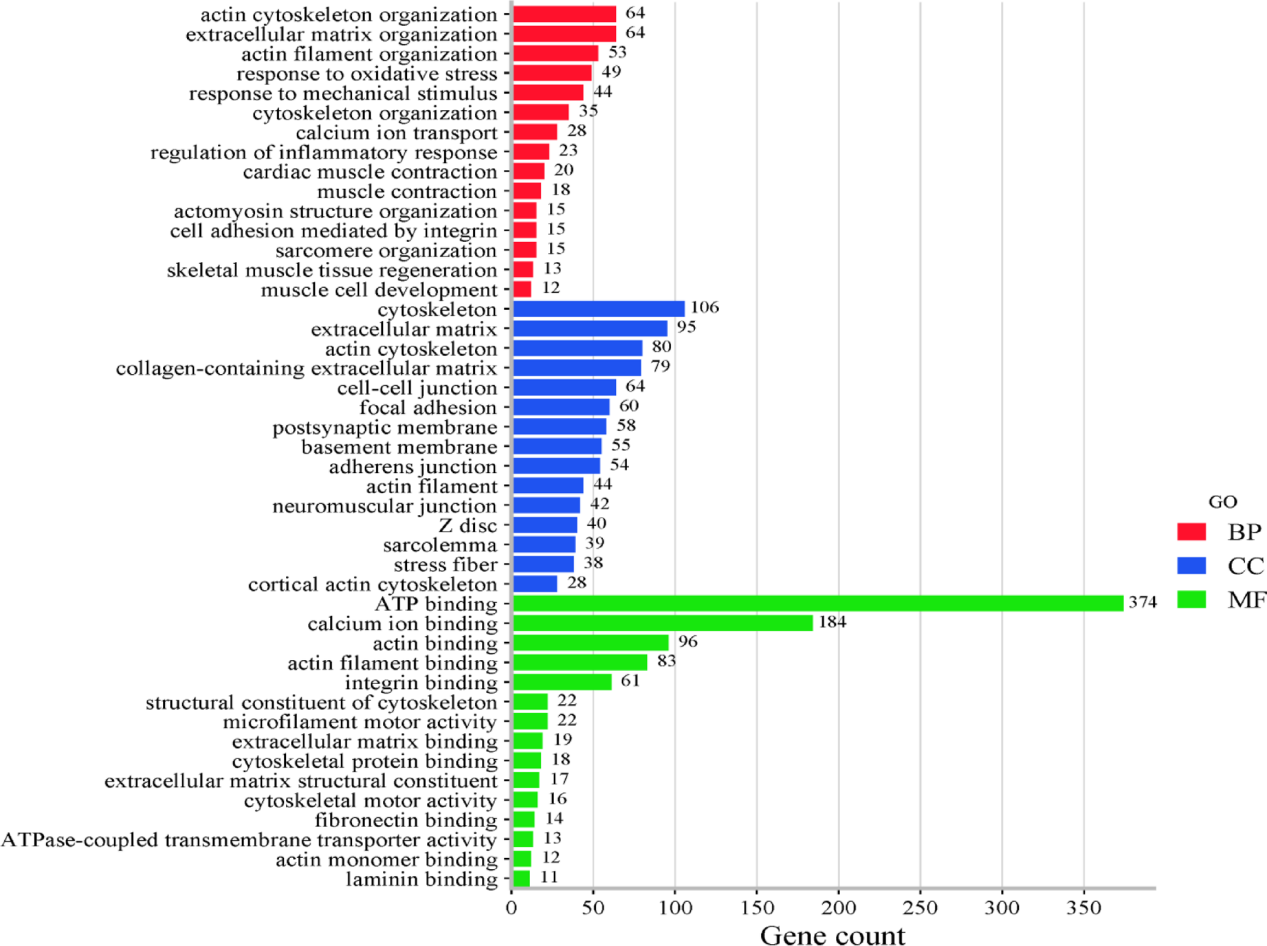
Top 15 enriched Gene Ontology (GO) terms categorized into Biological Processes (BP, red), Cellular Components (CC, blue), and Molecular Functions (MF, green), identified using DEGs in *DMD*^*mdx*^ vs. WT rat *biceps femoris* muscle samples. *DMD*^*mdx*^ – Sprague-Dawley *DMD*^*mdx*^ rats; WT – Sprague-Dawley wild-type rats; DEGs – differentially expressed genes.

Gene Ontology enrichment analysis showed that significant terms have emerged in BP, CC, and MF, pointing to significant disturbances in the dystrophic muscle tissue of *DMD*^*mdx*^ rats. In BP, the following were the most enriched terms: actin cytoskeleton organization (GO:0030036), extracellular matrix organization (GO:0030198), and actin filament organization (GO:0007015). CC enrichment analysis showed significant GO terms in the cytoskeleton (GO:0005856), extracellular matrix (GO:0031012), and actin cytoskeleton (GO:0015629). Under MF, the enriched terms included ATP binding (GO:0005524), calcium ion binding (GO:0005509), and actin-binding (GO:0003779) (Fig. 6; Table S4).

### Pathway analysis

**Fig. 7:**
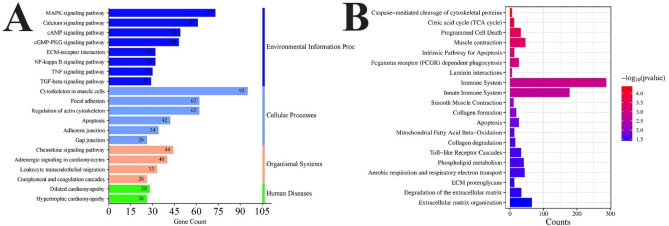
Pathway analysis of identified DEGs in *DMD*^*mdx*^ vs. WT rat *biceps femoris* muscle samples using **A**) KEGG and **B**) Reactome. KEGG pathways are sorted by the number of genes. The left column lists the target pathways, while the right column categorizes them according to their assigned KEGG classifications. Reactome pathways are sorted based on the p-value. *DMD*^*mdx*^ – Sprague-Dawley *DMD*^*mdx*^ rats; WT – Sprague-Dawley wild-type rats; KEGG – Kyoto Encyclopedia of Genes and Genomes DEGs – differentially expressed genes.

Pathway analysis was performed based on two databases: the Kyoto Encyclopedia of Genes and Genomes (KEGG) and Reactome. Both provided complementary information about which pathways are dysregulated in the *DMD*^*mdx*^ rat model (when compared to WT rats). KEGG pathway analysis emphasized specific, more essential pathways based on the number of genes. Among the top enriched pathways were the cytoskeleton in muscle cells, MAPK signaling pathway, focal adhesion, regulation of actin cytoskeleton, and the calcium signaling pathway. These pathways will be relevant in maintaining muscle structure, cellular signaling, and contraction. (Fig. 7A; Table S5). The Reactome pathway analysis highlighted immune-related and structural pathways. The top pathways include those related to the immune system, innate immune system, extracellular matrix organization, and muscle contraction (Fig. 7B; Table S6).

## Discussion

Duchenne muscular dystrophy is a severe genetic disorder characterized by progressive muscle degeneration and weakness, resulting from mutations in the *Dmd* gene that encodes dystrophin, a key protein in maintaining the integrity of muscle cells^23^. This research work employed the *DMD*^*mdx*^ rat model to investigate the molecular changes characteristic of the pathology of DMD in the *biceps femoris* muscle. Animals were analyzed at 4 months of age when pathology was known to be established, although not as severe as at later time points^5^. This age was, therefore, relevant for analyzing some pathways that future therapeutics could target.

### Histopathological and biochemical assessment

The histopathological picture of the *biceps femoris* muscle samples was typical for muscular dystrophy, containing extensive myofiber necrosis, inflammatory infiltration dominated by mononuclear cells, and significant interstitial fibrosis in the *DMD*^*mdx*^ group, which was not observed in WT rats (Fig. 1). Still, it was similar to the observations of Larcher et al. (2014)^5^ made on the same animal model, underlining the pathological severity associated with dystrophin deficiency. In *biceps femoris* of *DMD*^*mdx*^ rats, sprouting myoblasts (satellite cells) surround the margins of myofibers, indicating that the regenerative process is still active but insufficient (Fig. 1). Quantitative analysis verified significant differences in muscle morphology of *DMD*^*mdx*^ rats compared to WT, including decreased myofiber diameter (Fig. 2A) and increased fibrous connective tissue area (Fig. 2B-D), characteristic of DMD progression^24,25^. These structural changes lead to the failure of muscle function, as seen in DMD^26,27^. This study’s biochemical and antioxidative status data provide essential information regarding DMD’s systemic and muscular effects as modeled in Sprague-Dawley *DMD*^*mdx*^ rats. Such high levels of muscle-specific enzymes (CPK, LDH, ALT) in *DMD*^*mdx*^ rats (Fig. 3) correlate well with the muscle damage and membrane instability characteristic of DMD^28^ due to the absence of dystrophin^29^. Moreover, lower amylase activity in *DMD*^*mdx*^ rats indicates potential differences in metabolic or pancreatic function that require further exploration.

Several electrolyte disturbances were detected in the *DMD*^*mdx*^ rats, including a decrease in calcium and sodium and an increase in phosphorus, magnesium, and potassium (Fig. 3), similar to those observed in other cases of muscular dystrophy and possibly associated with dysregulated muscle homeostasis, including dysregulation of ion channels and muscle degeneration^24,29,30^. Above and beyond the local intra-/inter-tissue interactions that might occur, global metabolic changes reflect the dystrophic background by producing systemic alterations such as hyperglycemia and lower levels of urea measured in the blood plasma of *DMD*^*mdx*^ rats (Fig. 3). These metabolic dysregulations are known to occur in muscle diseases. They may also be related to the alteration of the normal systemic response to exercise or stimulus observed in muscle diseases^31^.

The antioxidative status analysis reveals a significant oxidative stress burden in *DMD*^*mdx*^ rats. Decreased TAS, GSH reductase, and GSH peroxidase activity indicate impaired antioxidant defenses, while elevated TBARS levels (Fig. 4) highlight increased lipid peroxidation, a hallmark of oxidative damage in dystrophic muscle. These findings align with the oxidative stress hypothesis of DMD progression, where excess reactive oxygen species (ROS) amplify muscle damage and inflammation^32–34^. Interestingly, the absence of significant differences in SOD activity between *DMD*^*mdx*^ and WT rats suggests selective impacts on specific antioxidant pathways (Fig. 4).

The analysis of 4-month-old *DMD*^*mdx*^ rats captures an intermediate stage of DMD pathology, where muscle degeneration, inflammation, and fibrosis are established but not as severe as in older rats^5^. Our findings likely reflect early-to-mid-stage molecular and histopathological changes compared to aged *DMD*^*mdx*^ rats (e.g., 12 months), which exhibit more pronounced fibrosis and muscle loss^6^. This age was chosen to identify pathways amenable to therapeutic intervention before irreversible damage occurs. Future studies exploring specific signaling pathways with expression changes identified in this study, comparing younger and older *DMD*^*mdx*^ rats, will help clarify how these molecular profiles evolve with disease progression. The analyses presented above, together with the earlier study by Larcher et al. (2014)^5^, conducted using the same *DMD*^*mdx*^ rat model, provide valuable insight into the pathophysiology of DMD and form the basis for the primary analysis presented in this study, in which we focused on detailed gene expression profiling using RNA-seq to characterize the skeletal muscle transcriptomic landscape as a foundation for further research involving the *DMD*^*mdx*^ model. Differences in histopathological, biochemical, and antioxidative status parameters can provide important context for interpreting molecular mechanisms and identifying new therapeutic targets in DMD.

### Gene expression analysis

In our NGS analysis, we identified 3,615 differentially expressed genes (DEGs), with significant enrichment in pathways related to muscle contraction, actin cytoskeleton, and extracellular matrix (ECM) organization (Fig. 5). Identified biological processes are critical for maintaining muscle fiber structure and function^35^. Their dysregulation reflects core pathological features of DMD, including fiber degeneration and impaired repair^36,37^. Changes in cytoskeletal components and ECM-related genes underscore the loss of cellular integrity in dystrophic muscle^38,39^. Moreover, Disruption of actin dynamics contributes to sarcolemmal instability^40^, while alterations in ATP and calcium-binding functions impair contraction and energy metabolism^41,42^. Reduced expression of actin-binding proteins further destabilizes muscle structure and regeneration^43,44^ (Fig. 6). The pathway enrichment performed in our study via KEGG and Reactome showed complementarity: KEGG emphasized cytoskeletal regulation and MAPK signaling, while Reactome highlighted inflammation and ECM remodeling. These findings align with hallmark DMD pathology (Fig. 7)^45–47–49^. Moreover, we observed downregulation of key structural genes: *Dmd* (–1.2), *Actc1* (–2.7), *Actn1* (–1.0), *Col6a1* (–1.7), and *Mmp2* (–1.4), indicating impaired contraction, ECM integrity, and cytoskeleton function^14,50,51^. From a therapeutic perspective, modulating these genes or pathways, for instance by enhancing Mmp2-mediated muscle repair, may aid in limiting muscle damage. Reduced expression of *Actb* (–0.8), *Actg1* (–0.9), and *Flna* (–0.8) suggests disrupted myogenic differentiation and enhanced fibrosis, as also supported by decreased Col5a1 (-1.39) and Col6a1^50^. Notably, *Myh3* (–4.4), *Myh8* (–4.3), and *Actc1* (-2.7) downregulation further reflect impaired regeneration, compounded by the absence of dystrophin, resulting in muscle weakness, fragility, and susceptibility to necrosis.

### Muscle degeneration and regeneration

DMD involves progressive muscle degeneration due to dystrophin deficiency, impairing regeneration. Chronic inflammation and fibrosis further hinder muscle repair, leading to functional decline^52^. In our study, on the molecular level, the genes *Myh9* (-0.8), *Myh3*, and *Actn1* are downregulated, which causes decreased contractility of muscle fibers. These genes are important in providing the mechanical contractions essential in skeletal muscle twitch, and their loss results in poor muscle function. Russell et al. (2023), who investigated the role of myosin inhibition in protecting skeletal muscles in dystrophic *mdx* mice, found that reducing muscle contraction through selective myosin inhibition protected against muscle stress injury without compromising strength. This suggests modulating myosin activity could be a promising therapeutic approach for improving muscle function in DMD patients^53^.

The downregulation of *Igf1* (-1.3) is significant for muscle regeneration, as it is crucial for satellite cell activation. Research indicates that lower levels of *Igf-1* impair satellite cell proliferation and differentiation, which are essential for muscle repair following injury^54,55^. Conversely, the increased expression of cardiac muscle genes such as *Tbx5* (3.03) and *Myh6* (1.89) indicates a compensatory response to muscle degeneration. This response, however, is insufficient to counterbalance the loss of skeletal muscle-specific contractile proteins, highlighting a maladaptive shift toward cardiac muscle gene expression in the context of skeletal muscle injury^56^. Interfering with the signaling pathways that regulate the *Igf1* gene could enhance satellite cell activation and muscle formation^57^.

It is also important to mention that several critical pathways are affected in DMD, such as PI3K/AKT signaling, calcium homeostasis, and cytoskeleton remodeling pathways. In our study, some genes belonging to the abovementioned pathways, including *Akt3* (-0.7), *Egfr* (-0.9), and *Rock1* (-1.02), were downregulated. These findings further support the idea of their role in disrupting muscle regeneration and enhancing fibrosis. Specifically, interfering with such molecular pathways, including the PI3K/AKT signaling pathway, which is crucial for muscle regeneration, may create a management approach to the degenerative progression of DMD^58^. Preventing fibrosis-related pathways, perhaps *Rock1* and *Pdgfrα* (-1.63), may also decrease fibrotic tissue, replacing functional muscles in DMD patients^59^.

### Fibrosis and ECM remodeling

In DMD, fibrosis results from excessive ECM remodeling, driven by chronic inflammation and muscle degeneration, leading to tissue stiffness and impaired muscle function^60^. Fibrosis, characterized by the deposition of ECM proteins, is another key pathophysiological feature in *mdx* mice^61^. In our study, several collagen genes, such as *Col1a1* (-2.09), *Col1a2* (-2.03), and *Col6a1* (-1.07), were found to be downregulated in *DMD*^*mdx*^ rats. These findings align with prior research and indicate a reduction in collagen synthesis as fibrosis advances^61^. However, the early stages of the disease are marked by extensive ECM production, resulting in fibrotic scarring. Interestingly, certain MMPs, such as *Mmp2*, are downregulated, reflecting decreased ECM turnover. Conversely, Mmp10 is upregulated, highlighting the intricate nature of the remodeling process^62^.

Fibrosis significantly contributes to the progression and worsening of Duchenne Muscular Dystrophy. *Tgfβ1* gene signaling (-1.3) modifies muscle atrophy and non-functional regeneration in DMD. The study by Mázala et al. (2020) aimed to understand *Tgfβ*-mediated muscle degeneration and the effects of early stages of regenerative failure under the *D2-mdx* mice model. *Tgfβ* signaling suppression was demonstrated to prevent muscle wasting by preventing the build-up of fibro-adipogenic progenitor (FAP) in a model study. At the same time, it failed to restore normal myogenesis^63^. Serrano, Rodriguez, and Muñoz-Cánoves (2017) have discussed this in detail regarding fibrosis development in muscles. According to their review, treatments targeting *Tgfβ* and other pathways might reduce the extent of fibrosis and positively impact muscle function^64^.

### Oxidative stress

Chronic oxidative stress defines DMD because it substantially drives muscle deterioration and inflammatory processes. Anatomical defects from dystrophin elimination impair muscle cell stability while raising mechanical vulnerability and calcium overload, damaging mitochondrial functions, and generating high ROS levels^33^. Oxidative stress directly impacts proteins, lipids, and DNA, accelerating muscle degeneration and fibrosis progression and activating inflammatory pathways^65,66^. In DMD, pathological processes accelerate as antioxidant defenses struggle against oxidative stress, leading to further muscle damage and necrosis^34^. Research indicates that targeting oxidative stress alongside its related pathways presents the potential to manage DMD^67^.

The muscle cell generates ROS mainly from mitochondria, while mitochondrial dysfunction substantially leads to oxidative stress that affects DMD patients^68^. In our research, *Actn1* (-1.00) and *Myh3* (-4.40) genes exhibit decreased expression levels, which may be the consequence of impaired muscle contractions and distorted energy metabolism properties associated directly with mitochondrial dysfunction. The observed decrease in *Akt3* (-0.71) expression levels may suggest that the essential PI3K/AKT pathway cannot activate properly to promote cell survival and oxidative stress responses^69^. Moreover, an elevated expression level of *Keap1* (0.54) can block the signaling of NRF2, the antioxidant system master regulator. In DMD, increased ROS damage occurs due to the cells’ inability to counteract oxidative stress, as the body’s antioxidant system fails to function correctly. The diminished expression of *Tgfβ1* (-1.30) identified in our study may interfere with redox balance and inflammatory regulation, causing disturbances in antioxidant responses^33^. Research indicates that decreased *Tgfβr2* (-0.90) receptor levels also limit antioxidant protection against oxidative stress^70^.

The development of fibrosis in DMD is closely connected to oxidative stress and ECM modifications. Muscle cells can become vulnerable to oxidative damage because *Col6a1* (-1.70) and *Col5a1* (-1.39) show decreased expression, leading to ECM breakdown in these muscles^71^. Reduced fibronectin expression, *Fn1( -1.13)*, impairs ECM remodeling, weakens cellular defense against oxidative stress, and contributes to ECM deterioration in dystrophic muscles^72^. The lower levels of *Mmp2* (-1.40) enhance the protection of tissues in the short term but also stimulate fibrotic changes, leading to more oxidative damage^73^. In addition, DMD leads to prolonged inflammation, which generates ROS primarily through the entry of immune cells and cytokine production. The elevated expression of *Tnfrsf19* (3.24) reflects amplified TNF signaling that fuels inflammatory and oxidative damage^74^. The *Dkk1* (4.48) elevates its levels and turns off Wnt pathway operation, leading to increased oxidative stress and disturbances in muscle regeneration^75^. The decreased expression of *Coro1a* (-0.84) indicates a weakened immune cell function and promotes chronic inflammation^76^.

Elevated intracellular calcium plays a central role in DMD pathogenesis because it causes oxidative damage through protease activation and inflammatory pathways^77,78^. The identified in our study, *Atp2b2* (1.91), together with *Atp1a2* upregulation, point to a potential compensatory response against calcium overload, but it proves inadequate to stop oxidative damage^79^. The decreased expression of *Pdgfrα* (-1.63) in DMD indicates dysfunctional mechanisms for responding to oxidative stress, which can worsen muscle destruction^59^. The damage pathway of DMD shows complexity because of abnormal calcium and oxidative stress regulation systems.

### Inflammation and immune response

In DMD, chronic inflammation exacerbates muscle degeneration, triggering an immune response that further damages tissue, creating a cycle of inflammation and immune-mediated injury^80^. When inflammation becomes chronic, macrophages and other immune cells sustain inflammation, leading to increased fibrosis. Juban et al. (2018) identified that pro-inflammatory macrophages correlate with fibrosis in DMD. They found that Ly6C-positive macrophages had pro-fibrotic activity through the maintenance of fibroblast collagen I synthesis by integrating high latent *Tgfβ1* (-1.3) production related to *Ltbp4* expression. However, it was delineated that AMPK activation inhibited the *Ltbp4* gene, lowering the fibrosis rate and enhancing the muscle force in the dystrophic mice^81^. It has been proposed that the pathophysiology of muscular dystrophy involves both regenerative and inflammatory mechanisms^82,83^. As the disease advances, immune homeostasis is impaired by the downregulation of *Lgals3* (-2.3)^84^ and *Cd44* (-1.8)^85^, related to immune cell recruitment^83^. Also, *Nos2* (1.45) in oxidative stress is increased while the inflammation is still active.

**Fig. 8:**
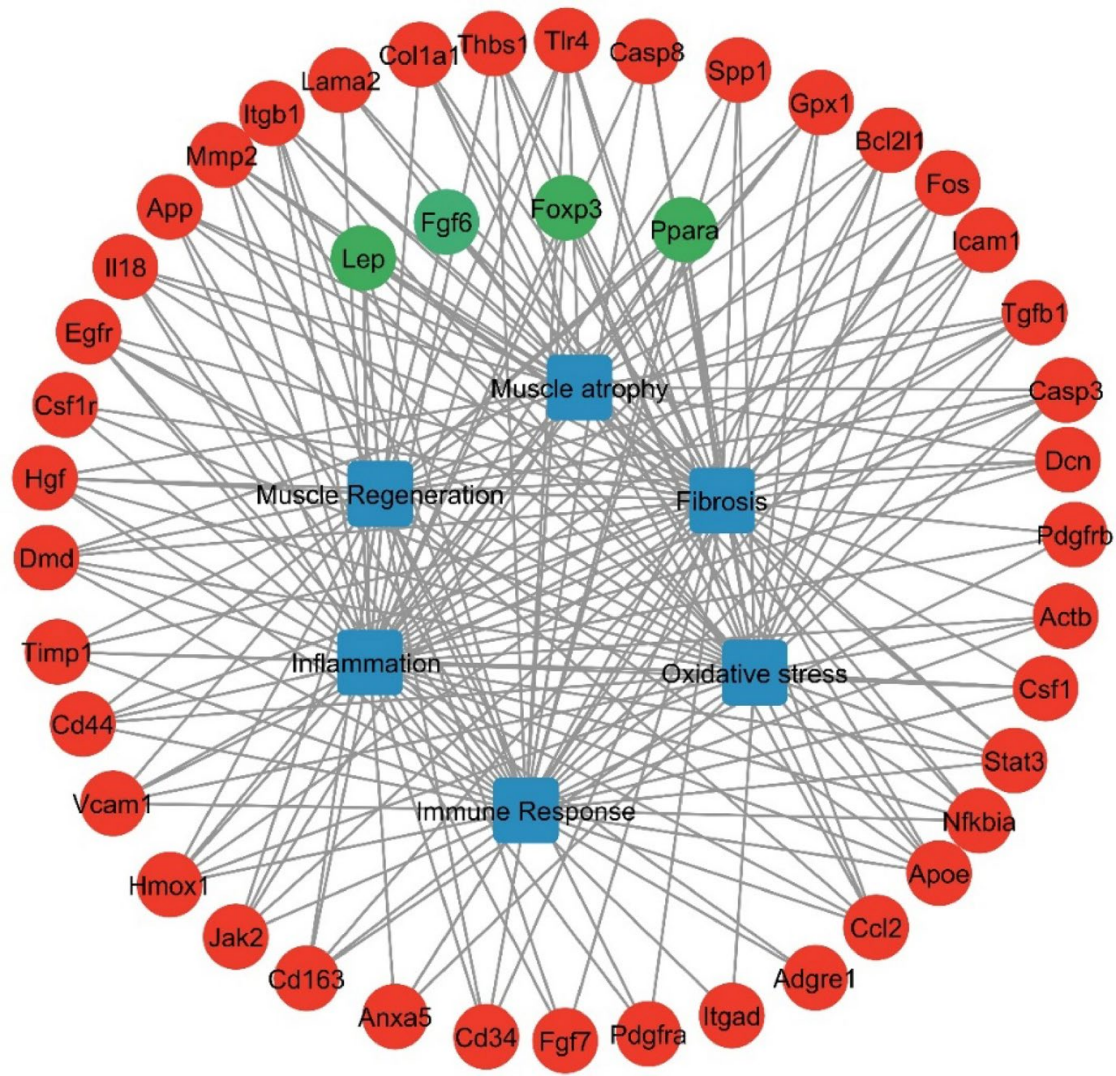
Relevance network of identified DEGs. Nodes in the network represent genes, while the expression status is indicated through color coding, where downregulated genes appear red and upregulated genes show green. Blue squares identify DMD-related biological processes.

In summary, we constructed a gene interaction network using the STRING plugin in Cytoscape to integrate the key processes implicated in DMD progression. This network highlights significant relationships among the DEGs identified in *DMD*^*mdx*^ rats, specifically those associated with the major pathological pathways discussed above. The associated GO terms exhibited high connectivity within the network, underscoring their central role in DMD pathogenesis (Fig. 8).

### Comparison with the *mdx* mouse model

In the *mdx* mouse model, a critical animal model for DMD, the primary gene implicated is the *Dmd* gene itself, characterized by a spontaneous mutation that disrupts dystrophin transcription and translation. Transcriptomic analyses of `*mdx*` muscle have identified several other genes exhibiting significant expression changes relevant to the disease phenotype. Notably, *Myh3* and *Myh8*, regeneration-linked isoforms of myosin heavy chain genes, show marked alterations. Furthermore, other myosin heavy chain genes, including *Myh2*,* Myh4*, and *Myh7*, alongside the SERCA Ca(2+)-ATPase gene *(Atp2a1)*, demonstrate differential expression patterns contributing to the understanding of muscle pathology in this model^86^. Another study shows that key pathways include cytoskeleton and ECM organization, mechanotransduction, and Ras, Rho, and Wnt signaling. Genes like *Sspn*, *Rho* family GTPases, and “*collagen*” genes are involved in compensatory remodeling and maintaining muscle integrity^87^. However, the *DMD*^*mdx*^ rat model exhibited more DEGs (3,615 vs. approximately 2,000–3,000 in *mdx* mice), likely reflecting the more severe muscle pathology observed in rats, as previously reported by Larcher et al.^5^. Notably, the downregulation of *Mmp2* in our study contrasts with its upregulation in some *mdx* mouse studies, suggesting species-specific differences in ECM remodeling. Previous studies, as well as our own results, highlight the *DMD*^*mdx*^ rat model as a complementary model with potentially greater translational relevance due to its closer mimicry of human DMD pathology. Further studies are needed, including a meta-analysis of RNA-seq data from humans, rats, and mice, which should allow for the identification of key differences relevant to selecting the most appropriate experimental model for human DMD.

### Advantages of the *DMD*^*mdx*^ rat model and alignment with human DMD

The *DMD*^*mdx*^ rat model offers significant advantages over the *mdx* mouse model due to its larger body size, more severe muscle pathology, and closer resemblance to human DMD, including advanced fibrosis and dilated cardiomyopathy^5,6^. This model more accurately reflects the gradual loss of muscle function and the multisystemic nature of the disease, which in humans affects not only the muscular system but also the cardiovascular and respiratory systems. The larger size of the animals enables the application of more precise experimental and functional procedures, such as muscle strength measurements, biopsies, local injections, and cardiac assessments using echocardiography. Our RNA-seq data align with human DMD studies^14^, showing dysregulation in muscle contraction (e.g., *Actc1*, *Myh3*), ECM organization (e.g., *Col6a1*), and inflammation (e.g., *Nos2*), consistent with human muscle biopsy transcriptomes. Moreover, in the rat model, we also observe elevated oxidative stress and gene alterations related to calcium homeostasis and cytoskeletal remodeling, which are likewise reported in human DMD. Unlike *mdx* mice, which exhibit strong muscle regeneration^9^, partly due to a large pool of active satellite cells and adequate compensation by utrophin, the limited regenerative potential of *DMD*^*mdx*^ rats more accurately reflects the chronic and progressive nature of the disease in humans. Rats do not show the same level of compensatory utrophin expression, resulting in a more pronounced dystrophic phenotype, including persistent muscle damage, increased fibrosis, and reduced tissue regeneration capacity. This makes the *DMD*^*mdx*^ rat model significantly more suitable for translational studies, particularly for evaluating the efficacy of therapeutic interventions at early and intermediate stages of the disease (Table 1). However, despite its advantages, the *DMD*^*mdx*^ rat model also has certain limitations. These include higher maintenance costs, fewer standardized protocols compared to the well-characterized *mdx* mouse model, and, as with other models, physiological differences that may affect the translational relevance of some findings.

**Table 1:** Core and contextual genes dysregulated in the *DMD*^*mdx*^ rat model.

	Log₂FC	Function / Relevance	Pathways / Processes	Therapeutic Implications	References
**Core Gene**					
*Dmd*	-1.2	Dystrophin protein; structural anchor	Membrane integrity	Gene therapy (e.g., exon skipping, AAV-based delivery), stop codon readthrough	^24^
*Actc1*	-2.7	Actin isoform; sarcomeric structure	Actin cytoskeleton	Targeting sarcomere stabilization to support contractile apparatus	
*Actn1*	-1.0	Z-disc actin cross-linker	Cytoskeletal integrity	Stabilizing cytoskeleton; potential biomarker for sarcomere disorganization	
*Myh3*	-4.4	Embryonic myosin; regeneration marker	Muscle repair	Myogenic regeneration enhancement (e.g., myosin-targeted therapies)	
*Myh8*	-4.3	Perinatal myosin; regenerating muscle	Regeneration	Stimulating expression may enhance myogenic regeneration	
*Igf1*	-1.3	Growth factor for satellite cell activation	PI3K/AKT signaling	IGF1-based therapies or agonists to enhance muscle regeneration	^54,57^
*Col6a1*	-1.7	ECM collagen VI	ECM structure, fibrosis	Antifibrotic therapies; gene reactivation to restore ECM resilience	^50]– [51^
*Mmp2*	-1.4	ECM degradation enzyme	ECM turnover, fibrosis	Balanced activation to reduce fibrosis and promote remodeling	^50,62^
*Tgfβ1*	-1.3	Pro-fibrotic cytokine	Fibrosis, inflammation	Anti-TGFβ therapies to reduce fibrosis and improve regeneration	^33^
*Akt3*	-0.7	PI3K/AKT pathway kinase	Cell survival, redox homeostasis	Activation may protect against oxidative stress and promote muscle repair	^58,69^
*Rock1*	-1.02	Cytoskeletal regulator	Cytoskeleton remodeling	ROCK inhibitors may reduce fibrosis and cytoskeletal disorganization	^58]– [59^
*Pdgfra*	-1.63	Fibro/adipogenic progenitor regulator	Fibrosis initiation	Anti-PDGFRα therapies to suppress fibroblast activation	^59^
**Contextual Gene**					
*Myh6*	1.89	Cardiac myosin; maladaptive shift	Cardiac gene expression	May indicate compensatory remodeling; monitor to avoid cardiac burden	
*Tbx5*	3.03	Cardiac transcription factor	Cardiac activation	Possible off-target effect of compensatory expression in skeletal muscle	^56^
*Egfr*	-0.9	Growth/survival receptor	PI3K/AKT, MAPK	EGFR modulation could restore muscle growth capacity	^58^
*Col1a1*	-2.09	Fibrillar collagen; ECM structural support	Fibrosis, ECM integrity	Anti-fibrotic therapy targeting collagen production in early-stage disease	^61^
*Col1a2*	-2.03	Partner of Col1a1; contributes to ECM rigidity	Fibrosis	Anti-fibrotic therapy targeting collagen production	^61^
*Mmp10*	4.22	ECM degradation	ECM remodeling	May serve as biomarker for active tissue remodeling/fibrosis	^62^
*Keap1*	0.54	NRF2 inhibitor; suppresses antioxidant response	Redox imbalance, oxidative stress	NRF2 activators could counter oxidative damage by inhibiting Keap1	
*Tnfrsf19*	3.24	TNF receptor; inflammation driver	TNF signaling	TNF blockers may mitigate inflammatory tissue damage	^74^
*Dkk1*	4.48	Wnt pathway inhibitor	Wnt signaling, regeneration	Wnt agonists could restore regenerative signaling impaired by Dkk1	^75^
*Nos2*	1.45	iNOS; reactive nitrogen species production	Inflammation, oxidative stress	NOS2 inhibitors may reduce nitrosative stress and tissue injury	
*Fn1*	-1.13	ECM adhesion protein	ECM remodeling	Enhancing fibronectin levels may aid in ECM repair	^72^
*Lgals3*	-2.3	Immune modulation	Immune cell recruitment	Galectin-3 supplementation or mimetics may restore immune function	^84^
*Cd44*	-1.8	Cell adhesion	Immune interaction	Therapeutic relevance in modulating inflammatory cell infiltration	^85^
*Atp2b2*	1.91	Ca²⁺ ATPase; calcium extrusion	Calcium homeostasis	Upregulation may reflect protective adaptation; target for calcium overload modulation	^79^
*Atp1a2*	0.91	Na⁺/K⁺-ATPase subunit	Calcium/ion regulation	May act as a compensatory marker; maintaining ionic balance	^79^
*Coro1a*	-0.84	Actin regulator in immune cells	Immune motility, inflammation	Targeting Coro1a could improve immune cell dynamics and reduce chronic inflammation	^76^

It should be noted, as a study limitation, that the *biceps femoris* muscle samples used for RNA-seq likely contained a heterogeneous cell population, including myofibers, satellite cells, immune cells (e.g., macrophages), and fibroblasts, particularly in *DMD*^*mdx*^ rats, due to inflammation and fibrosis. This cellular diversity may influence the transcriptomic profile, as upregulated immune-related genes (e.g., *Nos2*, *Tnfrsf19*) likely reflect immune cell infiltration, while downregulated muscle-specific genes (e.g., *Actc1*, *Myh3*) primarily originate from myofibers. Future single-cell RNA-seq studies could help dissect cell-type-specific contributions and provide a more precise interpretation of these molecular changes. Furthermore, as the analysis was performed on only one muscle type, future studies should include additional muscles, such as the diaphragm, a tissue severely affected in DMD and functionally critical in later stages of the disease, to more comprehensively characterize histopathological and molecular changes in the *DMD*^*mdx*^ rat model. Our transcriptomic profiling may facilitate this direction of research.

## Conclusion

Duchenne muscular dystrophy is a severe X-linked genetic disorder caused by mutations in the *Dmd* gene, leading to progressive loss of muscular strength. In this study, we further characterize the *DMD*^*mdx*^ rat model and have demonstrated that biochemical alterations, such as elevated ALT, LDH, CPK, and other markers, are closely associated with muscle damage and inflammation. Histopathological evaluation of *biceps femoris* muscle tissue reveals significant fiber attenuation, fibrosis, and mononuclear cell infiltration. Concurrently, quantitative gene analysis indicated the downregulation of genes involved in muscle contraction, ECM structure, and cytoskeletal organization, all contributing to the characteristic muscle phenotype in DMD. Pathways such as actin cytoskeleton organization, calcium signaling, PI3K/AKT, and MAPK were reported to be both enriched and impaired, promoting muscle atrophy and fibrosis. Therapeutic strategies for DMD include antisense therapies restoring dystrophin function, modulation of fibrosis-related genes such as *Col6a1* and *Mmp2*, and targeting signaling pathways like PI3K/AKT to enhance muscle regeneration and function. Additionally, anti-inflammatory approaches, including *Nos2* inhibitors and promoting the transition of macrophages from pro-inflammatory (M1) to pro-regenerative (M2) states, hold promise for mitigating chronic inflammation and improving disease outcomes. The studies described above provide a comprehensive transcriptomic characterization of the skeletal muscle of *DMD*^*mdx*^ rats, a model for Duchenne muscular dystrophy, whose results may support and guide the development of multifaceted therapeutic approaches targeting the molecular and cellular basis of DMD.

## Supplementary Information

Below is the link to the electronic supplementary material.

Supplementary Material 1

## Data Availability

The sequencing data have been deposited in the NCBI Sequence Read Archive (SRA) accession number SUB14872230; BioProject: PRJNA1191685, and are available from the corresponding author upon a reasonable request.

## Abbreviations

*Actb*: Actin beta
*Actc1*: Actin, alpha cardiac muscle 1
*Actg1*: Actin gamma 1
*Actn1*: Actinin alpha 1
*Akt3*: AKT serine/threonine kinase 3
ALP: Alkaline Phosphatase
ALT: Alanine Aminotransferase
*Atp2b2*: ATPase plasma membrane Ca2 + transporting 2
BMD: Becker muscular dystrophy
BP: Biological Process (GO category)
*Casp3*: Caspase 3
*Casp9*: Caspase 9
CC: Cellular Component (GO category)
*Cd44*: CD44 molecule (Indian blood group)
*Col1a1*: Collagen type I alpha 1 chain
*Col1a2*: Collagen type I alpha 2 chain
*Col5a1*: Collagen type V alpha 1 chain
*Col6a1*: Collagen type VI alpha 1 chain
*Coro1a*: Coronin 1 A
CPK: Creatine Phosphokinase
DEGs: Differentially expressed genes
*Dkk1*: Dickkopf WNT signaling pathway inhibitor 1
*Dmd*: Dystrophin
ECM: Extracellular Matrix
*Egfr*: Epidermal growth factor receptor
*Flna*: Filamin A
*Fn1*: Fibronectin 1
GFT: Gene Transfer Format
GGT: Gamma-Glutamyl Transferase
GO: Gene Ontology
GPX: Glutathione Peroxidase
GR: Glutathione Reductase
GSH: Glutathione (Reduced form)
H&E: Hematoxylin and eosin
HISAT2: Hierarchical Indexing for Spliced Transcripts 2
*Igf1*: Insulin-like growth factor 1
KEGG: Kyoto Encyclopedia of Genes and Genomes
LDH: Lactate Dehydrogenase
*Lgals3*: Galectin 3
*Ltbp4*: Latent transforming growth factor beta binding protein 4
*Ly6c*: Lymphocyte antigen 6 complex, locus C
MF: Molecular Function (GO category)
*Mmp10*: Matrix metallopeptidase 10
*Mmp2*: Matrix metallopeptidase 2
*Myh3*: Myosin heavy chain 3 (embryonic)
*Myh6*: Myosin heavy chain 6 (cardiac alpha)
*Myh8*: Myosin heavy chain 8 (perinatal)
*Myh9*: Myosin heavy chain 9 (non-muscle)
NGS: Next-generation sequencing
*Nos2*: Nitric oxide synthase 2 (inducible)
PCA: Principal Component Analysis
*Pdgfra*: Platelet-derived growth factor receptor alpha
PSR: Picrosirius red
*Rock1*: Rho-associated coiled-coil containing protein kinase 1
ROS: Reactive Oxygen Species
SOD: Superoxide Dismutase
SRA: Sequence Read Archive
TAS: Total Antioxidant Status
TBARS: Thiobarbituric Acid Reactive Substances
*Tbx5*: T-box transcription factor 5
*Tgfβ1*: Transforming growth factor beta 1
*Tnfrsf19*: Tumor necrosis factor receptor superfamily member 19
WT: Wild-type
