# Supplementary material for: Transcriptomic profiling of skeletal muscle in the DMDmdx rat model of Duchenne muscular dystrophy
Source: Sci Rep. 2025 Aug 11;15:29312. doi: 10.1038/s41598-025-14756-9 (PMC12340039; doi:10.1038/s41598-025-14756-9)
Supplement: Supplementary file 1 — Supplementary Material 1 [file 41598_2025_14756_MOESM1_ESM.zip › Table S1 & Figure S1.pdf]

Table S1. Summary of rat genome read mapping

	Total read	Mapped	Paired in sequencing	Read1	Read2	Properly paired	with itself and mate-mapped	Singletons
DMD^{mdx} 1	166162213	148184488	98047090	49023545	49023545	73816724	77427452	2641913
DMD^{mdx} 2	177706048	173333419	113718792	56859396	56859396	104548896	106617922	2728241
DMD^{mdx} 3	172923483	153843272	108985250	54492625	54492625	83127126	87604806	2300233
DMD^{mdx} 4	141439354	129338125	87871594	43935797	43935797	71324082	73939208	1831157
WT 1	171596716	164144524	126390858	63195429	63195429	112979998	115205004	3733662
WT 2	129686530	115797330	95752914	47876457	47876457	77431882	80484594	1379120
WT 3	136908149	134301437	97309986	48654993	48654993	92070198	93204570	1498704
WT 4	138869298	135010564	98299460	49149730	49149730	90855224	92353404	2087322

DMD^{mdx} – Sprague-Dawley DMD^{mdx} rats; WT – Sprague-Dawley wild-type rats.

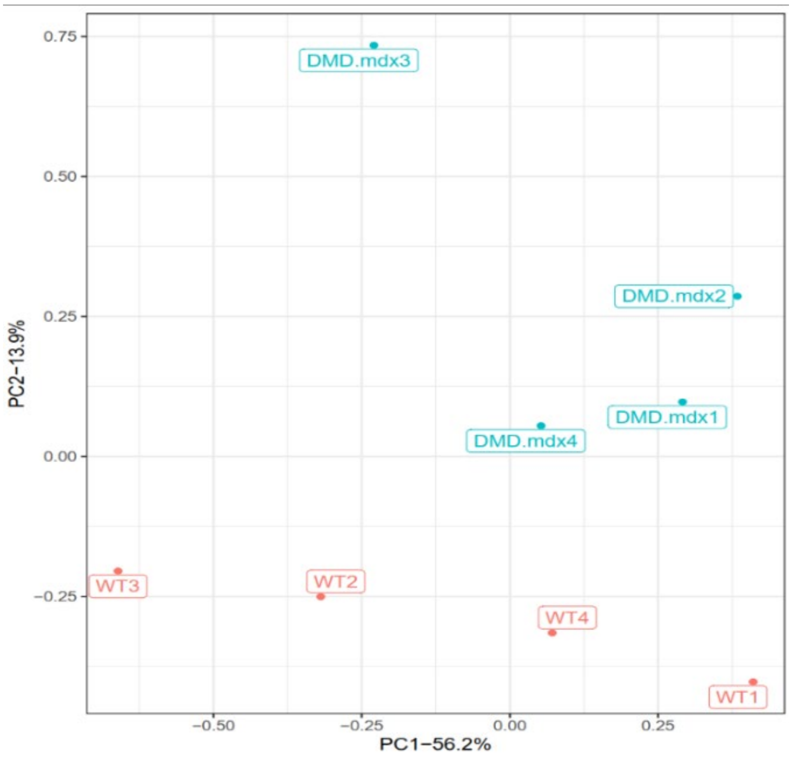

Fig. S1. The PCA plot shows the separation between the gene expression profiles of DMD^{mdx} and WT rat skeletal muscle samples. DMD^{mdx} samples (DMD^{mdx} 1-4) are highlighted in blue; WT samples (WT 1-4) are highlighted in red. DMD^{mdx} – Sprague-Dawley DMD^{mdx} rats; WT – Sprague-Dawley wild-type rats; n=4 per genotype.

Table S1. Differentially expressed genes (DEGs) up-regulated in DMDmdx vs WT rat skeletal muscle

GeneName	GeneStableID	log2FoldChange	pvalue	padj
Smg5l1	ENSRNOG00000024507	6.430291594	0.003034842	NA
Krt32	ENSRNOG00000013611	6.257480888	0.000661905	NA
Dcaf12l2	ENSRNOG00000031533	5.94961312	0.005800785	NA
Npy4r	ENSRNOG00000061026	5.857994455	0.009436106	NA
Zfp42	ENSRNOG00000067356	5.721723904	0.035694182	NA
Pvrig	ENSRNOG00000051906	5.695989803	0.010568421	NA
Or1ad6	ENSRNOG00000003668	5.682216876	0.009263559	NA
Ugt2b	ENSRNOG00000069670	5.562598779	0.014289324	NA
Scp2d1	ENSRNOG00000009461	5.558210946	0.003119973	NA
Or2d36	ENSRNOG00000068532	5.358837498	0.019962493	NA
Akr1c1	ENSRNOG00000038331	5.355209949	0.026274723	NA
Krtap7-1	ENSRNOG00000043017	5.354035184	0.023489907	NA
Or52n1	ENSRNOG00000070647	5.305089764	0.022549118	NA
Sprr2g	ENSRNOG00000071130	5.293744755	0.023281646	NA
Or4f59	ENSRNOG00000070396	5.246138609	0.025501803	NA
Defb22	ENSRNOG00000007525	5.232181213	0.028021942	NA
Nfilz	ENSRNOG00000063608	5.211032773	0.001830324	NA
Taar7c	ENSRNOG00000071165	5.174754608	0.027628696	NA
Wfdc15a	ENSRNOG00000053099	5.149853636	0.030440296	NA
Glra3	ENSRNOG00000049792	5.146317979	0.007024555	NA
Vom1r38	ENSRNOG00000065923	5.141528357	0.036921212	NA
St8sia3	ENSRNOG00000018305	5.095273711	0.032158424	NA
Or4c103b	ENSRNOG00000070601	5.093789434	0.033877957	NA
Hrh4	ENSRNOG00000016887	5.090613557	0.03051552	NA
Asic5	ENSRNOG00000011842	5.068740519	0.038547331	NA
Vom2r72	ENSRNOG00000066629	5.035170952	0.038188522	NA
Or5an10	ENSRNOG00000068291	4.993353232	0.014873788	NA
Clrn3	ENSRNOG00000028288	4.976240468	0.041208494	NA
Ndufb2	ENSRNOG00000026616	4.968483401	0.040667461	NA
Taar7h	ENSRNOG00000063959	4.959261797	0.048087891	NA
Krtap13-1	ENSRNOG00000042531	4.949642819	0.045474187	NA
Wfdc21	ENSRNOG00000029668	4.943345907	0.046287459	NA
Serpina3m	ENSRNOG00000009921	4.903873342	0.012559314	NA
Dnase2b	ENSRNOG00000016262	4.900201252	0.010825908	NA
Prpmp5	ENSRNOG00000010485	4.885197029	0.004021185	NA
Or2ak4e	ENSRNOG00000064663	4.884979819	0.049400837	NA
Slc5a7	ENSRNOG00000010597	4.786624184	0.009363553	NA
Or12j3	ENSRNOG00000068160	4.741093066	0.015313292	NA
Cuzd1	ENSRNOG00000029945	4.734974844	0.019298029	NA
Bpifa1	ENSRNOG00000013859	4.710276307	0.015179541	NA
Cd209a	ENSRNOG00000040128	4.647801337	0.009470711	NA
Cd209e	ENSRNOG00000028803	4.645421051	0.010403796	NA
Dhrs7l1	ENSRNOG00000025648	4.61226925	0.026356813	NA
Fcrl6	ENSRNOG00000000055	4.585766082	0.020247096	NA
Vom1r32	ENSRNOG00000067757	4.560037226	0.017446432	NA
Nxf7	ENSRNOG00000023256	4.494769922	0.02155432	NA
Scgb2b2	ENSRNOG00000023367	4.493518281	0.035618225	NA

Dkk1	ENSRNOG00000048688	4.486070723	0.022952072	NA
Pgk2	ENSRNOG00000013600	4.477337851	0.027966391	NA
Gm47791	ENSRNOG00000067340	4.464954615	0.035671558	NA
Faiml	ENSRNOG00000048802	4.457021242	0.025939358	NA
Cmtm2a	ENSRNOG00000025420	4.446277308	0.021891553	NA
Armc12	ENSRNOG00000000508	4.430218541	0.007127137	NA
Prss3	ENSRNOG00000068192	4.419764658	0.04023016	NA
Cldn17	ENSRNOG00000027691	4.403330171	0.029411888	NA
Tex28	ENSRNOG00000051438	4.374746951	0.019899803	NA
Barx2	ENSRNOG00000008592	4.332142576	1.78E-16	9.90E-14
Awat1	ENSRNOG00000026231	4.317874661	0.047147774	NA
Rad21l1	ENSRNOG00000022335	4.313108691	0.036927443	NA
Or9a4	ENSRNOG00000066955	4.253787126	0.014448372	NA
Nmur2	ENSRNOG00000014081	4.251560788	0.028738916	NA
Or5ar1	ENSRNOG00000009624	4.23577614	0.014164269	NA
Lrrc10	ENSRNOG00000005555	4.226786604	0.008174605	NA
Or13a19	ENSRNOG00000070653	4.218774511	0.013337237	NA
Mmp10	ENSRNOG00000032832	4.216907797	0.043024596	NA
Clpsl2	ENSRNOG00000038807	4.214441765	0.048184984	NA
Vom1r6	ENSRNOG00000065978	4.15980036	0.049926044	NA
Or2y15	ENSRNOG00000069386	4.148478601	0.049259957	NA
Slurp2	ENSRNOG00000031437	4.144004934	0.005998403	NA
Tff3	ENSRNOG00000001159	4.120404823	0.027146965	NA
Tex46	ENSRNOG00000022257	4.100065638	0.018491424	NA
Slc10a4	ENSRNOG00000026091	3.980606258	0.010791339	NA
Or4d11	ENSRNOG00000070229	3.975929035	0.006156628	NA
Rps19l1	ENSRNOG00000031474	3.931594537	0.046043587	NA
Tmem212	ENSRNOG00000012608	3.92657491	0.025379998	NA
Or2t1	ENSRNOG00000068637	3.901263162	0.046708105	NA
Moxd2	ENSRNOG00000012916	3.874893402	0.035322791	NA
Or10h1d	ENSRNOG00000050907	3.860727253	0.023994759	NA
Lypd4	ENSRNOG00000020098	3.860329642	0.021610435	NA
Lrit2	ENSRNOG00000022726	3.855369122	0.02592357	NA
Saxo1	ENSRNOG00000048197	3.851743827	0.007963558	NA
Percc1	ENSRNOG00000065484	3.84686151	0.024112769	NA
Aqp4	ENSRNOG00000016043	3.835981727	3.01E-12	7.97E-10
Htr1d	ENSRNOG00000012038	3.805886358	0.025847494	NA
Nrl	ENSRNOG00000018528	3.797279456	0.023608958	NA
NMS	ENSRNOG00000038979	3.784679345	0.046440678	NA
Slc22a25	ENSRNOG00000017964	3.732782735	0.04157368	NA
Krt88	ENSRNOG00000008242	3.711361469	0.010611989	NA
Slc17a4	ENSRNOG00000022505	3.699071131	0.033779761	NA
Or52x1	ENSRNOG00000017379	3.694436692	0.047084008	NA
Fam43b	ENSRNOG00000043113	3.688002596	0.048291093	NA
Crnn	ENSRNOG00000008721	3.668744904	0.034130529	NA
Rs1	ENSRNOG00000030434	3.658859371	0.038569692	NA
Nrsn1	ENSRNOG00000017444	3.653197401	0.039127923	NA
Ccl26	ENSRNOG00000001444	3.652588436	0.020428944	NA
Rnf17	ENSRNOG00000008059	3.651340019	0.013162061	NA
Ces2e	ENSRNOG00000011635	3.649280265	0.027819675	NA

Pramef12	ENSRNOG00000037035	3.639412866	0.03181166	NA
Or10g3c	ENSRNOG00000064358	3.634683231	0.025879633	NA
Pdilt	ENSRNOG00000015368	3.634189149	0.026713675	NA
Brinp1	ENSRNOG00000005561	3.634092727	0.011781822	NA
Sbk2	ENSRNOG00000038625	3.628299687	2.30E-08	2.39E-06
Tm4sf4	ENSRNOG00000016437	3.611112098	0.020350484	NA
Slc25a54	ENSRNOG00000027522	3.607786227	0.041998402	NA
Apon	ENSRNOG00000033466	3.589071704	0.033555197	NA
Or52b2	ENSRNOG00000025819	3.58756394	0.026855833	NA
Fshr	ENSRNOG00000016783	3.585575953	0.038500049	NA
FAM187A	ENSRNOG00000002918	3.561141965	0.009707817	NA
Apoa4	ENSRNOG00000055909	3.559559469	0.0288849	NA
Tlx3	ENSRNOG00000004976	3.55509893	0.011728181	NA
Cdh8	ENSRNOG00000056643	3.533162276	0.005224295	NA
Adad1	ENSRNOG00000017246	3.520636949	0.0439722	NA
Gpr50	ENSRNOG00000011335	3.519498836	0.043612575	NA
Sox3	ENSRNOG00000058173	3.509137899	0.040052332	NA
Bhmt2	ENSRNOG00000040120	3.497065779	0.021583789	NA
Zp4	ENSRNOG00000027506	3.487433763	0.016744328	NA
Spry3	ENSRNOG00000060153	3.487408472	0.047576863	NA
Kprp	ENSRNOG00000028865	3.485446247	0.014369462	NA
Ttll2	ENSRNOG00000033901	3.476801355	0.040657135	NA
Tex45	ENSRNOG00000000973	3.475841164	0.019540158	NA
Cbln4	ENSRNOG00000004372	3.475596257	0.022333117	NA
Lmod1	ENSRNOG00000051548	3.475535751	3.70E-08	3.65E-06
Clca4	ENSRNOG00000029889	3.462937003	0.025280258	NA
Wdr72	ENSRNOG00000054889	3.46080863	0.016537685	NA
Slc35f4	ENSRNOG00000014982	3.458762898	0.046992479	NA
Cyp1a1	ENSRNOG00000019500	3.457974315	0.012002462	NA
Panx3	ENSRNOG00000031675	3.442803129	0.030139273	NA
Rptn	ENSRNOG00000018679	3.442723976	0.044774613	NA
Or10ad1	ENSRNOG00000064488	3.441941382	0.045012305	NA
Atp6v0d2	ENSRNOG00000006926	3.435560092	0.017393894	NA
Gal	ENSRNOG00000015156	3.433812601	0.036833522	NA
Gk2	ENSRNOG00000029397	3.424093373	0.018430668	NA
Pla2g2f	ENSRNOG00000016798	3.418649479	0.017253259	NA
Neto1	ENSRNOG00000014006	3.396818227	0.013749386	NA
Caly	ENSRNOG00000018337	3.390630346	2.17E-05	0.00084757
Or13p3	ENSRNOG00000062918	3.376936665	0.04567214	NA
Cryga	ENSRNOG00000014790	3.375850558	0.003100025	NA
Vwc2	ENSRNOG00000049076	3.367022021	0.029158523	NA
Bhlha9	ENSRNOG00000022232	3.361918151	0.016910774	NA
Abhd12b	ENSRNOG00000026855	3.35195149	0.014475311	NA
Slc12a1	ENSRNOG00000005367	3.347430767	0.022847406	NA
Slc22a8	ENSRNOG00000018086	3.332202863	0.004040804	0.046807227
Dynlrb2	ENSRNOG00000012450	3.319902838	0.020314382	NA
Adm2	ENSRNOG00000029830	3.319304411	0.040319723	NA
Glycam1	ENSRNOG00000036826	3.313824491	0.000466586	NA
Wnt3a	ENSRNOG00000003039	3.310542684	0.013465429	NA
Lrit3	ENSRNOG00000061097	3.297197745	0.023557543	NA

Prap1	ENSRNOG00000018446	3.293826196	0.015567222	NA
Defb36	ENSRNOG00000007558	3.28353436	0.024549369	NA
Spata31d3	ENSRNOG00000056413	3.275780629	0.035742089	NA
Ucma	ENSRNOG00000017987	3.268994752	0.041917929	NA
Gja8	ENSRNOG00000046703	3.265266993	0.040451439	NA
Pck1	ENSRNOG00000028616	3.261980283	0.000225029	0.00542716
Ldc1	ENSRNOG00000024788	3.261532832	0.03437185	NA
Or2y1d	ENSRNOG00000063155	3.251060844	0.028782832	NA
Gjb6	ENSRNOG00000022116	3.248908958	0.007438015	0.069809805
Spaca7	ENSRNOG00000054006	3.246041019	0.02005762	NA
Tnfrsf19	ENSRNOG00000014265	3.244248517	1.01E-05	0.00046191
Lcn10	ENSRNOG00000028074	3.236513405	0.029460002	NA
Cyp2c22	ENSRNOG00000021924	3.236495528	0.038836936	NA
Il5ra	ENSRNOG00000005954	3.231851932	0.030430135	NA
Tyrp1	ENSRNOG00000029318	3.230079597	0.034665254	NA
Lypd5	ENSRNOG00000019432	3.223166198	0.026282308	NA
Lta	ENSRNOG00000000838	3.217422155	0.042642059	NA
Abcb5	ENSRNOG00000006398	3.21308977	0.01063061	NA
Katnal2	ENSRNOG00000017788	3.206057364	0.024874746	NA
Or5a1	ENSRNOG00000067177	3.193024799	0.032904134	NA
Ccdc42	ENSRNOG00000004075	3.186461713	0.018603521	NA
Tmem266	ENSRNOG00000015201	3.164152475	0.000264239	0.006237653
Cd207	ENSRNOG00000013222	3.161427542	0.018769715	NA
Adipoq	ENSRNOG00000001821	3.16085153	0.000294381	0.006774676
Lipc	ENSRNOG00000060338	3.158499702	0.027193462	NA
Ces2c	ENSRNOG00000036571	3.156750013	0.013065585	NA
Mucl3	ENSRNOG00000042287	3.149750103	0.023873747	NA
Acnat1	ENSRNOG00000065119	3.14540708	0.034694743	NA
Actbl2	ENSRNOG00000043292	3.144574823	0.034184844	NA
Or6d14	ENSRNOG00000070550	3.12697055	0.026657778	NA
Or2ak4	ENSRNOG00000067952	3.123499921	0.039102469	NA
Chst4	ENSRNOG00000016953	3.122914474	0.03952095	NA
Fndc11	ENSRNOG00000013243	3.122665576	0.024858321	NA
Acot3	ENSRNOG00000053460	3.119991035	0.037494981	NA
Pnma8c	ENSRNOG00000063049	3.117742103	0.008349144	NA
Efcab10	ENSRNOG00000010730	3.117068747	0.01584967	NA
Adarb2	ENSRNOG00000030775	3.097055275	0.00021765	0.005315026
Clca4l	ENSRNOG00000036877	3.093160215	0.047637341	NA
Bpifa2	ENSRNOG00000013540	3.083920182	0.023038171	NA
C8b	ENSRNOG00000007639	3.081077849	0.024717077	NA
Nrsn2	ENSRNOG00000023935	3.079104354	0.032805884	NA
Gys2	ENSRNOG00000059753	3.054984052	0.00372267	NA
Igbp1b	ENSRNOG00000062370	3.045908756	0.038176286	NA
Slc25a52	ENSRNOG00000033251	3.03441235	0.013677089	NA
Cdh10	ENSRNOG00000009771	3.034079807	0.048240288	NA
Azgp1	ENSRNOG00000001333	3.033736531	0.021584277	NA
Tbx5	ENSRNOG00000001399	3.031719142	0.020397829	NA
Spata31d1	ENSRNOG00000069288	3.027977589	0.004896251	0.053122277
Tdrd15	ENSRNOG00000060052	3.026407502	0.009499718	NA
Qrfprl	ENSRNOG00000006782	3.02382198	0.002349643	NA

Dgat2l6	ENSRNOG00000038046	3.019230163	0.03040016	NA
Odad2	ENSRNOG00000018905	3.018812198	0.014356085	NA
Htr5a	ENSRNOG00000007066	3.016572786	0.036221262	NA
Tex52	ENSRNOG00000062735	3.012864357	0.045313923	NA
Cldn24	ENSRNOG00000031521	3.004923405	0.038553436	NA
Aspdh	ENSRNOG00000019424	2.998610964	0.00857395	NA
Slc22a2	ENSRNOG00000016625	2.990786155	0.019512277	NA
Igsf5	ENSRNOG00000028216	2.984777543	0.028705597	NA
Gpr6	ENSRNOG00000049580	2.972889678	0.045583441	NA
Cfap92	ENSRNOG00000031408	2.970224098	0.02926662	0.164255741
Slc9c2	ENSRNOG00000002890	2.968943666	0.000545034	NA
Or51s1	ENSRNOG00000015444	2.966394001	0.038209926	NA
Msh4	ENSRNOG00000010431	2.962002033	0.012157859	NA
Sh2d7	ENSRNOG00000023870	2.959288814	8.92E-05	0.002667761
Pde6g	ENSRNOG00000069361	2.946804647	0.010398171	NA
Vom2r35	ENSRNOG00000050831	2.944357455	0.014354356	0.106954028
Gabrb3	ENSRNOG00000060599	2.942828962	0.049847709	NA
Car7	ENSRNOG00000012371	2.937685564	0.040344998	NA
Lactbl1	ENSRNOG00000065157	2.931392591	0.009203629	NA
Adh6	ENSRNOG00000069176	2.930228923	0.00818364	0.074693092
Slc35f3	ENSRNOG00000049456	2.927254059	0.015637946	0.113403432
Scg3	ENSRNOG00000010784	2.924810738	0.041979521	NA
Stox1	ENSRNOG00000023712	2.908984775	0.042572076	0.200517654
Dydc1	ENSRNOG00000011165	2.907376646	0.031803849	NA
Trpv5	ENSRNOG00000015394	2.907245363	0.042587101	NA
Cldn18	ENSRNOG00000030386	2.907243631	0.026064142	NA
Ces4a	ENSRNOG00000014257	2.892197692	0.039047006	NA
Vom1r39	ENSRNOG00000070447	2.891067475	0.048992253	NA
Vom1r100	ENSRNOG00000055950	2.885474543	0.044042124	NA
Tcp10b	ENSRNOG00000013374	2.879028311	0.038527516	NA
Morc2b	ENSRNOG00000004501	2.875945643	0.035449682	NA
Corin	ENSRNOG00000002302	2.875130025	0.007052803	0.067685168
Ttc24	ENSRNOG00000028765	2.865071106	0.020869428	0.134191368
Myo3b	ENSRNOG00000030022	2.863481219	0.001747906	0.026084644
Ido1	ENSRNOG00000031189	2.845971087	0.025076054	NA
Lrit1	ENSRNOG00000012790	2.845626741	0.035997984	NA
Kcnk15	ENSRNOG00000010816	2.843441926	0.01151646	NA
Or6ae1	ENSRNOG00000018955	2.83951252	0.020514237	NA
Cyp4a3	ENSRNOG00000066878	2.838361434	0.039267703	0.192750728
Gpr152	ENSRNOG00000021912	2.837326979	0.047145381	NA
Ces2h	ENSRNOG00000039079	2.827297767	0.04860577	NA
Wnt16	ENSRNOG00000005781	2.820272268	5.43E-05	0.001791724
Utf1	ENSRNOG00000050207	2.819684279	0.048922742	NA
Gm6569	ENSRNOG00000065138	2.809348683	0.048017802	NA
Prss30	ENSRNOG00000033266	2.809111739	0.04784576	NA
Ccdc116	ENSRNOG00000001860	2.804736008	0.049789742	NA
Gkn3	ENSRNOG00000037782	2.800433013	0.033737605	NA
Aqp3	ENSRNOG00000009797	2.797959556	0.031664825	NA
Kng1	ENSRNOG00000030387	2.791315581	0.044648949	NA
Slc15a1	ENSRNOG00000011598	2.790682756	0.025028734	0.149290838

Snorc	ENSRNOG00000016606	2.790370056	0.044005377	NA
Rbp4	ENSRNOG00000015518	2.789565344	0.000172323	0.004484835
Il22ra1	ENSRNOG00000066331	2.781444775	0.03854805	0.190605048
Cidec	ENSRNOG00000009153	2.780160931	0.001624661	0.024913597
M1ap	ENSRNOG00000007288	2.778764002	0.019507635	NA
Tmem40	ENSRNOG00000010430	2.777670348	0.023413353	NA
Bhlhe23	ENSRNOG00000010312	2.777113392	0.025966504	NA
Krt83	ENSRNOG00000030814	2.774157887	0.028553458	0.161592035
Adgrb2	ENSRNOG00000014375	2.769035549	0.02370251	0.144647512
Lrtm2	ENSRNOG00000007508	2.765480318	0.044752098	0.204995198
Ttc22	ENSRNOG00000007189	2.758137504	0.044876966	0.205238046
Dmgdh	ENSRNOG00000023588	2.755753841	0.041726789	0.198504848
Lep	ENSRNOG00000045797	2.753233434	0.007183682	0.068453681
Slc44a5	ENSRNOG00000042332	2.750450838	0.02554303	NA
Bpifb1	ENSRNOG00000015468	2.750050019	0.04306611	0.201613578
Irx4	ENSRNOG00000012720	2.745766173	0.016077593	0.11473343
Marchf11	ENSRNOG00000024461	2.733915559	0.045197911	NA
Slc34a2	ENSRNOG00000004626	2.733532061	0.011801548	0.094134295
Dnah12	ENSRNOG00000059865	2.73062049	0.007611832	0.071055928
Slc22a16	ENSRNOG00000063767	2.723307781	0.028137023	NA
Pdzph1	ENSRNOG00000011042	2.720793734	0.041723674	NA
Glra1	ENSRNOG00000013588	2.720634503	0.034593202	0.180074202
H2bc1	ENSRNOG00000069905	2.716016975	0.035100144	NA
Foxi1	ENSRNOG00000006293	2.712374251	0.049853098	NA
Bpifa6	ENSRNOG00000043258	2.706237263	0.032744895	NA
Ahrr	ENSRNOG00000014721	2.70573058	0.015898491	0.114199398
Prss8	ENSRNOG00000019616	2.68698642	0.004346659	0.049002131
Qrfpr	ENSRNOG00000014414	2.685494362	0.031621111	NA
Or2c1	ENSRNOG00000039773	2.679808962	0.021495994	NA
Krt86	ENSRNOG00000008367	2.677109979	0.021763064	0.137077168
Plaat5	ENSRNOG00000023528	2.666611462	0.012489424	NA
Epha6	ENSRNOG00000029184	2.661924527	0.011735227	0.093905927
Pnpla3	ENSRNOG00000022268	2.6598726	0.002247861	0.030751888
Slc14a2	ENSRNOG00000061714	2.655415342	0.021391942	0.135882992
Btbd17	ENSRNOG00000003109	2.652120926	0.047445936	NA
Vom1r19	ENSRNOG00000063416	2.647480333	0.024564294	0.14787933
Amigo2	ENSRNOG00000007032	2.646480613	0.019437714	NA
Matn3	ENSRNOG00000056141	2.642924572	0.036069429	NA
Got1l1	ENSRNOG00000038473	2.639694258	0.044050422	NA
Cd24	ENSRNOG00000000321	2.639042277	3.91E-13	1.22E-10
Necab2	ENSRNOG00000015084	2.6373717	0.007205439	0.06857712
Rd3	ENSRNOG00000050766	2.634931008	0.022751774	NA
Retn	ENSRNOG00000001001	2.631588385	0.038337933	NA
Diras2	ENSRNOG00000062731	2.629150746	0.003762642	0.04456093
Pthlh	ENSRNOG00000069267	2.624889066	0.015623498	NA
Tmem139	ENSRNOG00000063089	2.619057002	0.034770531	NA
Egfem1	ENSRNOG00000023908	2.617615078	0.026638597	NA
Hs6st3	ENSRNOG00000037886	2.612017172	0.03119091	NA
Or8s8	ENSRNOG00000070632	2.605651369	0.037592405	0.187865853
Loxhd1	ENSRNOG00000017410	2.590620393	0.029534309	0.165163151

Mylk4	ENSRNOG00000070083	2.58654196	2.93E-09	3.96E-07
Lrrc55	ENSRNOG00000028266	2.583955307	0.044281611	NA
Zp1	ENSRNOG00000020907	2.576231036	0.005352241	0.05622921
Pla2g3	ENSRNOG00000025121	2.56993643	0.027469515	0.158099388
Lgals5	ENSRNOG00000012557	2.566681805	0.012888044	NA
Dsg2	ENSRNOG00000016526	2.554102533	0.037693027	0.188283858
Asic3	ENSRNOG00000008380	2.552608769	0.00849422	0.076144963
Pwwp4	ENSRNOG00000068777	2.551369368	0.024148297	0.145882305
Tmem179	ENSRNOG00000013128	2.548861857	0.00089059	0.015893919
Ubqln3	ENSRNOG00000023833	2.545168367	0.027295868	NA
Dbh	ENSRNOG00000006641	2.541195348	0.046369559	0.209099198
Pxt1	ENSRNOG00000000516	2.54108634	0.00890897	NA
Fbxw12	ENSRNOG00000020720	2.537475938	0.049837464	NA
Sez6l	ENSRNOG00000025612	2.537031084	0.014245624	0.106602701
Odf3l2	ENSRNOG00000008199	2.532606523	0.000138263	0.00381938
Ihh	ENSRNOG00000018059	2.527787487	0.02118543	0.135099801
Atp6v1g2	ENSRNOG00000000840	2.516505393	0.01642051	0.116029163
Nell2	ENSRNOG00000006235	2.512179616	0.023488577	0.144007339
Rnf151	ENSRNOG00000014127	2.506931503	0.030760669	NA
Fabp12	ENSRNOG00000038825	2.506609269	0.043143949	NA
Otos	ENSRNOG00000016567	2.504607716	0.045422133	NA
Ajap1	ENSRNOG00000050137	2.50227963	0.011403403	0.091911548
Efcab3	ENSRNOG00000042255	2.500504353	0.046966227	0.210681663
Guca1a	ENSRNOG00000015402	2.500447827	0.048169864	NA
Nhlh1	ENSRNOG00000005166	2.499386823	0.044272677	0.204266287
Pcdh15	ENSRNOG00000000606	2.49451471	0.015436639	0.112647701
C14h4orf5	ENSRNOG00000038427	2.484257268	0.01507543	0.110947186
Inhbc	ENSRNOG00000007700	2.47349313	0.011461951	NA
Ptchd4	ENSRNOG00000012708	2.470411854	0.022728814	NA
Dlx6	ENSRNOG00000063630	2.469931668	0.028494744	NA
Cmtm1	ENSRNOG00000057711	2.467824548	0.033578962	NA
Ces1f	ENSRNOG00000070517	2.463101268	0.044591659	NA
Pilrb	ENSRNOG00000070677	2.461829056	0.031011295	0.169090722
Irx2	ENSRNOG00000012742	2.455778806	0.034324793	0.17921591
Unc5a	ENSRNOG00000059840	2.454279395	6.31E-09	7.93E-07
Tekt5	ENSRNOG00000002571	2.452400999	0.045407382	NA
Myt1l	ENSRNOG00000004269	2.451469374	0.030588413	0.167856722
Dbx1	ENSRNOG00000014706	2.43950574	0.047532489	NA
Kcnj16	ENSRNOG00000004713	2.437405333	0.034616375	0.180101905
Abcc12	ENSRNOG00000015666	2.436970765	0.047688434	0.212127047
Cyct	ENSRNOG00000024457	2.432379021	0.030978195	0.169031608
Dcc	ENSRNOG00000033099	2.425195841	0.027193924	0.157093562
Apob	ENSRNOG00000005542	2.421225145	0.034798423	0.180520548
Amer3	ENSRNOG00000023603	2.418743533	0.035515629	0.182087617
Noxa1	ENSRNOG00000009286	2.408489891	0.033951917	NA
Syt3	ENSRNOG00000019318	2.405103125	0.000205639	0.005134396
Six3	ENSRNOG00000057031	2.400577355	0.043721898	0.203278776
Tssk5	ENSRNOG00000013753	2.400542417	0.015242428	0.111753896
Pgbd5	ENSRNOG00000026791	2.399097702	0.02881672	0.162702578
Adgra1	ENSRNOG00000054955	2.398873449	0.009702681	0.083158921

Bpi	ENSRNOG00000034195	2.390985951	0.015128833	0.111287639
Slc24a2	ENSRNOG00000008169	2.387137342	0.034893665	0.180734912
Pnck	ENSRNOG00000058317	2.386307199	0.032161667	NA
Lpcat2b	ENSRNOG00000055849	2.385952551	0.034326198	NA
Lrrc8e	ENSRNOG00000028460	2.383343171	0.013435587	0.102711699
Nrn1	ENSRNOG00000050767	2.381816163	0.032716188	NA
Nphs1	ENSRNOG00000020873	2.380596641	0.047695064	0.212127047
Krt72	ENSRNOG00000030876	2.376552655	0.046731056	NA
Asic4	ENSRNOG00000019985	2.37573912	0.013731419	0.104155557
Rims4	ENSRNOG00000010868	2.373970376	0.013422619	0.102662939
Rhbg	ENSRNOG00000019412	2.37252788	0.024851143	0.149030333
Asphd1	ENSRNOG00000027213	2.368393311	0.0362621	NA
Ascl3	ENSRNOG00000013591	2.367978873	0.01047002	NA
Cdhr3	ENSRNOG00000047455	2.363119293	1.87E-07	1.53E-05
Tnfsf14	ENSRNOG00000047730	2.358564392	0.026337316	NA
Hes5	ENSRNOG00000013850	2.357848199	0.023408147	NA
Pnma2	ENSRNOG00000009815	2.356967221	0.028082282	NA
F7	ENSRNOG00000032737	2.354440024	0.032610552	0.174355661
Kcnip4	ENSRNOG00000032350	2.351969383	0.02197447	NA
Myo5b	ENSRNOG00000014104	2.350994193	0.021946662	0.138041583
Vsig1	ENSRNOG00000054219	2.349199129	0.017505639	NA
Pcdh10	ENSRNOG00000031974	2.348872694	0.033393915	0.176544689
Phox2a	ENSRNOG00000019706	2.347642073	0.049815393	0.217889898
Slain1	ENSRNOG00000024125	2.346635896	0.007781766	0.072295713
Krt1	ENSRNOG00000028996	2.346272617	0.01977704	0.130318069
Adig	ENSRNOG00000065916	2.345048992	1.28E-08	1.46E-06
Inava	ENSRNOG00000025948	2.344144742	0.046933967	0.210681663
Dscam	ENSRNOG00000027992	2.338532969	0.035895056	0.182854236
Frrs1l	ENSRNOG00000043103	2.338011491	0.038184759	NA
Akap3	ENSRNOG00000059227	2.335363095	0.038134556	NA
Pacrg	ENSRNOG00000068261	2.334485701	0.011640801	NA
Cct6a-ps7	ENSRNOG00000069794	2.333125102	0.043906191	NA
Fam228a	ENSRNOG00000050114	2.33279891	0.034429039	NA
Pax3	ENSRNOG00000013670	2.33048849	0.002941886	0.037477172
Cacna1b	ENSRNOG00000004560	2.319181019	0.026142996	0.153585171
Kctd16	ENSRNOG00000069221	2.317134109	8.90E-06	0.000412759
Insm1	ENSRNOG00000068937	2.297864029	0.008922173	0.078938089
Zan	ENSRNOG00000067638	2.296289004	0.043685089	0.203228931
Galr1	ENSRNOG00000016654	2.294606755	0.038178561	NA
Or6b13	ENSRNOG00000065821	2.292867246	0.041473589	0.197722926
Iqcn	ENSRNOG00000068146	2.282826693	0.041249721	0.197343617
Lrrc37a	ENSRNOG00000042025	2.281576837	0.049094071	NA
Adad2	ENSRNOG00000015690	2.27755106	0.018524974	0.125105803
Slc7a14	ENSRNOG00000028097	2.276558302	0.030130935	0.166596846
Npffr1	ENSRNOG00000000559	2.274409943	0.013379081	0.102581002
Cntnap2	ENSRNOG00000006617	2.273962946	0.016556352	0.11645506
Grpr	ENSRNOG00000004124	2.272097993	0.022414723	NA
Crygb	ENSRNOG00000032926	2.267998756	0.019889234	NA
Pde6b	ENSRNOG00000000065	2.267041701	0.011352438	0.091642999
Creb3l3	ENSRNOG00000032202	2.265192417	0.038790717	0.191192463

Kcng3	ENSRNOG00000004535	2.263681659	0.012823812	NA
Mei1	ENSRNOG00000007003	2.261209162	0.033501839	0.176755387
Clca2	ENSRNOG00000013771	2.261176476	0.035157787	NA
Robo3	ENSRNOG00000030016	2.24858185	0.029710448	0.165553927
Stpg1	ENSRNOG00000031448	2.246612384	0.034460615	0.179624085
Slc9a3	ENSRNOG00000015159	2.241581942	0.025396111	0.150451285
Galnt9	ENSRNOG00000037476	2.23957198	0.044415697	0.204490707
Il27ra	ENSRNOG00000005729	2.238259981	0.032868941	0.175256022
Psca	ENSRNOG00000061376	2.235842479	0.027856871	NA
Lhfpl7	ENSRNOG00000051192	2.231603792	0.026514818	0.154893461
Nrg3	ENSRNOG00000063450	2.230232734	0.049910687	NA
Ppp1r14d	ENSRNOG00000012648	2.229649786	0.012406418	NA
Reep6	ENSRNOG00000033262	2.226061291	0.002039857	0.028913108
Tectb	ENSRNOG00000015671	2.226044431	7.03E-05	0.002196044
Wnt8b	ENSRNOG00000013817	2.221951258	0.043900612	NA
Nanos3	ENSRNOG00000031593	2.221870901	0.049520837	NA
Commd5	ENSRNOG00000004484	2.219976135	0.020867999	NA
Grhl3	ENSRNOG00000029427	2.219252413	0.032939759	0.175453483
Slc6a5	ENSRNOG00000031662	2.212430237	0.038631844	0.190666315
Zyg11a	ENSRNOG00000010885	2.209919154	0.038509324	NA
Shisa7	ENSRNOG00000016877	2.206309516	0.015968153	0.114372565
C2h1orf56	ENSRNOG00000021656	2.205717565	0.021453196	NA
Bmp8b	ENSRNOG00000033209	2.198642208	0.042014702	NA
Or6f2	ENSRNOG00000067810	2.197067367	0.043753574	0.203311687
Ccdc63	ENSRNOG00000034266	2.196303716	0.031605072	0.170737524
Best4	ENSRNOG00000018749	2.194547227	0.033659354	NA
Blk	ENSRNOG00000010798	2.189744667	0.039249475	0.192721971
Cdkl4	ENSRNOG00000040266	2.188627967	0.005776106	0.059361304
Madcam1	ENSRNOG00000008217	2.186680785	0.04498454	NA
Umodl	ENSRNOG00000037191	2.186093324	0.04060647	0.196154298
Rimbp3	ENSRNOG00000030452	2.18459842	0.022572244	0.140554646
Nim1k	ENSRNOG00000016353	2.17840937	0.011925736	NA
Syt2	ENSRNOG00000004756	2.175937963	0.044562273	0.204618632
Hcn4	ENSRNOG00000009450	2.172475815	0.030250684	0.166869718
Rhou	ENSRNOG00000062578	2.169872519	5.49E-10	8.91E-08
Tdrd9	ENSRNOG00000053631	2.16189275	0.035980228	0.182894602
Abca4	ENSRNOG00000012892	2.16135607	0.007221243	0.068643667
Ush2a	ENSRNOG00000003738	2.160880194	0.021137561	0.135023861
Tle6	ENSRNOG00000006350	2.157490249	0.01269198	0.098808964
Aloxe3	ENSRNOG00000007454	2.150286826	0.040847382	0.196691674
Hpd	ENSRNOG00000001338	2.148607473	0.006580642	0.064767154
Ttc21a	ENSRNOG00000034089	2.144473769	0.039806802	0.194173442
Tmem89	ENSRNOG00000031141	2.143166347	0.042423239	NA
Ggt7	ENSRNOG00000018441	2.142415027	0.01923296	0.128274625
Lgals12	ENSRNOG00000021207	2.129993316	0.019754228	0.130318069
Slc5a1	ENSRNOG00000017775	2.125009977	0.030912032	0.168926504
Arfgef3	ENSRNOG00000011460	2.12474654	0.02334711	0.143651291
Rsph6a	ENSRNOG00000061526	2.123242192	0.027279828	0.157356433
Cpne5	ENSRNOG00000000522	2.122051958	0.017991532	0.122512266
Tll2	ENSRNOG00000013519	2.1196642	0.046695563	0.210022193

Cdh20	ENSRNOG00000014556	2.107667801	0.044705743	NA
Acvr1c	ENSRNOG00000004828	2.106488487	0.025921923	0.152689437
Grip1	ENSRNOG00000004013	2.10641799	0.034140277	0.178611659
Ccdc33	ENSRNOG00000008219	2.105748505	0.033643385	0.176910609
Myl7	ENSRNOG00000014409	2.104849401	0.04164671	0.198366171
P3r3urf	ENSRNOG00000049874	2.104781954	0.021208391	NA
Fam83g	ENSRNOG00000027924	2.10350085	0.030813894	0.168614035
Stk32a	ENSRNOG00000027066	2.097387065	0.02794687	NA
Otop3	ENSRNOG00000053055	2.093643559	0.02393246	0.145367533
Lpo	ENSRNOG00000008422	2.086952474	0.049437124	0.217149813
Ksr2	ENSRNOG00000028630	2.082466679	0.047212081	0.211088481
Bhlha15	ENSRNOG00000025164	2.077607646	0.033008653	0.175640305
Dpysl5	ENSRNOG00000008996	2.072176154	1.63E-07	1.36E-05
Cdh4	ENSRNOG00000052405	2.071257431	0.033857028	0.177666724
Hrh3	ENSRNOG00000061153	2.063084296	0.040474454	0.195896858
Mybpc3	ENSRNOG00000012307	2.061411706	0.026558663	0.154996014
Sorcs3	ENSRNOG00000028832	2.056318449	0.026752922	0.155541632
Khk	ENSRNOG00000008047	2.050029015	0.037854234	NA
Spata20	ENSRNOG00000003273	2.046225585	0.013460971	0.102822398
Wnt5b	ENSRNOG00000008168	2.042871936	0.016249075	0.115464338
Plin1	ENSRNOG00000015086	2.042041802	0.005763386	0.059297985
Ccdc154	ENSRNOG00000030541	2.038602154	0.040802573	0.196690621
Chst13	ENSRNOG00000025930	2.038555424	0.042483571	NA
Dcst1	ENSRNOG00000020621	2.03825674	0.030222186	0.166816097
Ccdc78	ENSRNOG00000022331	2.034858651	0.000105545	0.003056502
Mpl	ENSRNOG00000028377	2.033406231	0.044544804	0.204618632
Sytl5	ENSRNOG00000003585	2.032730818	0.036753128	NA
Smoc1	ENSRNOG00000005998	2.030593191	0.036871395	0.185653992
Bspry	ENSRNOG00000015105	2.028888936	0.020897336	NA
Bpifb3	ENSRNOG00000034174	2.028223687	0.035907583	0.182854236
Abcc2	ENSRNOG00000046727	2.025534918	0.035118543	0.181018762
Msx3	ENSRNOG00000046776	2.025319016	0.013810535	NA
Gucy2g	ENSRNOG00000015724	2.023681765	1.75E-05	0.000708233
Casp16	ENSRNOG00000067255	2.022796364	0.003807157	0.044901974
Cysrt1	ENSRNOG00000010600	2.021958315	0.02055732	NA
Dcst2	ENSRNOG00000058618	2.020964565	0.049578737	0.217465295
Rrlt	ENSRNOG00000063217	2.017011752	2.79E-06	0.000156997
Cdk18	ENSRNOG00000008137	2.010658615	4.30E-05	0.001508227
Gabrr3	ENSRNOG00000001679	2.008841594	0.049056275	NA
Ltk	ENSRNOG00000025130	2.007005958	0.035578433	0.182219586
Myo1a	ENSRNOG00000004177	2.006692609	0.043194501	0.202017492
Fbxo44	ENSRNOG00000009298	2.003717633	7.48E-06	0.000358406
Stra6	ENSRNOG00000008312	2.002137323	0.036573396	0.185004384
Tdrd12	ENSRNOG00000012719	2.001236747	0.032383699	0.173500012
Mb	ENSRNOG00000004583	1.998121374	4.46E-05	0.00154707
Acsbg3	ENSRNOG00000047549	1.989419275	0.041095089	0.197185549
Mrap	ENSRNOG00000021524	1.987788118	0.025759668	NA
C5h9orf15	ENSRNOG00000012068	1.986502675	0.04387458	NA
Sftpb	ENSRNOG00000010761	1.984757214	0.041128824	0.19728666
Cacng7	ENSRNOG00000056257	1.980721697	6.93E-05	0.002171026

Dbp	ENSRNOG00000021027	1.978368139	0.000100097	0.002948042
Cel	ENSRNOG00000010406	1.972630034	0.047626104	0.212070624
Slc26a9	ENSRNOG00000029514	1.971914217	0.000721234	0.013603908
Hoxa11	ENSRNOG00000059870	1.970804467	0.017013006	0.118331535
Gpr149	ENSRNOG00000014793	1.969459937	0.036553914	NA
Pitx1	ENSRNOG00000011423	1.969334545	0.03369023	0.176910609
Tmem25	ENSRNOG00000014218	1.967822458	0.000140005	0.003860664
Pdia2	ENSRNOG00000063310	1.965238883	0.037757471	0.188364201
Ankef1	ENSRNOG00000005792	1.964936795	0.00897171	0.079239931
Wasf3	ENSRNOG00000048898	1.963248371	0.025010655	0.149288993
Nppc	ENSRNOG00000018854	1.961567327	1.54E-05	0.000641924
Atp2c2	ENSRNOG00000049334	1.960505294	0.04449468	0.204618632
Iqca1	ENSRNOG00000019592	1.957545754	0.004809771	0.052476356
Cyp2a2	ENSRNOG00000069320	1.954829666	0.035442051	NA
Oprd1	ENSRNOG00000010531	1.950813628	0.044754999	NA
Mylk3	ENSRNOG00000017546	1.950250493	0.030114138	0.166596846
Lgi3	ENSRNOG00000011323	1.950088562	2.95E-05	0.001099618
Hnf1a	ENSRNOG00000001183	1.947553825	0.030524364	0.167648975
Pde6a	ENSRNOG00000017816	1.945604423	0.018495711	0.12501656
Lhx2	ENSRNOG00000010551	1.943160007	0.006800786	0.066016355
Atp4a	ENSRNOG00000020985	1.935367649	0.027232883	0.157202043
Hhipl2	ENSRNOG00000056400	1.934638757	0.021383584	0.135882992
Rab36	ENSRNOG00000001311	1.933262287	0.044196149	0.204142982
Dhcr24	ENSRNOG00000006787	1.931583333	5.65E-06	0.000283719
Mmel1	ENSRNOG00000012593	1.930622509	0.0186692	0.125414757
Dnah9	ENSRNOG00000004171	1.92899284	0.013306091	0.102324236
Rtbdn	ENSRNOG00000043215	1.919127113	0.048072178	NA
Stab2	ENSRNOG00000038948	1.918208578	0.016311264	0.115565935
Otud7a	ENSRNOG00000015503	1.912117787	0.021993148	0.138222366
Atp2b2	ENSRNOG00000030269	1.911037775	0.044060579	0.204001135
Nudt17	ENSRNOG00000037217	1.904471231	0.014027268	NA
Cirop	ENSRNOG00000057827	1.901006142	0.027169704	NA
Pkp3	ENSRNOG00000015152	1.899503692	0.030718453	0.168222669
Myh6	ENSRNOG00000025757	1.897453804	0.028070194	0.160136806
Tmprss9	ENSRNOG00000032429	1.88977873	0.049718531	0.217706658
Pcsk9	ENSRNOG00000006280	1.88819196	0.01418377	0.106568425
Vsig8	ENSRNOG00000008668	1.881187726	0.031633611	0.170773272
Sphkap	ENSRNOG00000016388	1.878294574	0.047284904	0.211088481
Tekt3	ENSRNOG00000027212	1.876070719	0.033318673	NA
Tmem239	ENSRNOG00000047004	1.87435777	0.022202449	0.138947902
Trim10	ENSRNOG00000000782	1.873532336	0.049589365	NA
Hoga1	ENSRNOG00000029501	1.87209527	2.14E-07	1.71E-05
Dnah7	ENSRNOG00000060984	1.86836634	0.045873542	0.208077543
Grap2	ENSRNOG00000018316	1.866884126	0.024142656	0.145882305
Alkal2	ENSRNOG00000005154	1.866417275	0.02263147	NA
Spock1	ENSRNOG00000012747	1.860492283	0.030715974	0.168222669
Hs3st3b1	ENSRNOG00000003384	1.855572652	0.032725452	NA
Mxd3	ENSRNOG00000070523	1.853498771	0.036257097	NA
Baiap2l2	ENSRNOG00000012215	1.85205763	0.000724146	0.013642316
Adamts16	ENSRNOG00000016812	1.849119652	0.00834414	0.075450782

Slc9a4	ENSRNOG00000015306	1.848459494	0.02526345	0.150049714
Fam221a	ENSRNOG00000009447	1.847729966	0.039676118	NA
Angptl6	ENSRNOG00000020603	1.847102214	0.04780296	0.212245686
Sv2b	ENSRNOG00000011160	1.838104352	0.012370683	0.097095841
Inpp5j	ENSRNOG00000019361	1.833147944	0.011828833	0.094267634
Kif26b	ENSRNOG00000028624	1.823136768	0.032122162	0.172692646
Ccdc181	ENSRNOG00000002860	1.820674166	2.77E-06	0.000156966
Dnah17	ENSRNOG00000003028	1.819421468	0.035370207	0.181690679
Fbxo41	ENSRNOG00000033202	1.818905884	0.013365364	0.102527019
Xpnpep2	ENSRNOG00000004009	1.818301311	0.004595647	0.050816307
Pex5l	ENSRNOG00000011211	1.812076765	0.043003299	0.201561792
Ush1g	ENSRNOG00000028456	1.81186087	0.043610186	0.203030868
Grin2a	ENSRNOG00000033942	1.804892288	0.044760366	0.204995198
Mpped2	ENSRNOG00000004863	1.798594573	0.000154862	0.00413142
Accsl	ENSRNOG00000042533	1.793766819	0.035681648	0.18254949
Tmprss6	ENSRNOG00000000184	1.791971501	0.027679432	0.158896664
Tjp3	ENSRNOG00000020501	1.789820363	0.029811639	0.165759994
Tssk4	ENSRNOG00000019789	1.786830113	0.046009055	NA
Tmem150c	ENSRNOG00000002258	1.784714868	1.40E-06	8.88E-05
Srrm4	ENSRNOG00000001141	1.783852656	0.037597287	0.187865853
Heatr9	ENSRNOG00000037100	1.783410412	0.024962864	0.1492604
Cabp7	ENSRNOG00000057703	1.781009353	0.029073358	0.163524521
Spag4	ENSRNOG00000048056	1.779625135	0.025110862	0.149608885
Sorl1	ENSRNOG00000064634	1.774919735	5.36E-09	6.79E-07
Mettl24	ENSRNOG00000024221	1.768466335	0.039880324	0.19426514
Sbk3	ENSRNOG00000042927	1.767260295	0.035689714	0.18254949
Duox2	ENSRNOG00000017395	1.76421215	0.048177835	0.2131882
Pcsk4	ENSRNOG00000016405	1.764066956	0.001142226	0.019094296
Lingo1	ENSRNOG00000017193	1.762032866	0.047282787	0.211088481
Acat2l1	ENSRNOG00000013975	1.760022659	0.047854603	0.21235395
Fam83e	ENSRNOG00000021039	1.756808835	0.029740975	0.165565884
Tnfrsf18	ENSRNOG00000022012	1.750919273	0.024745781	NA
Fam174b	ENSRNOG00000012369	1.748107577	0.041600628	NA
Nfasc	ENSRNOG00000030515	1.744018283	0.002108839	0.029467008
Igfals	ENSRNOG00000015061	1.743158321	0.015573495	0.113222141
Atg9b	ENSRNOG00000023449	1.742099249	0.023645729	0.144470765
Nat8l	ENSRNOG00000049351	1.740085191	0.017946063	0.122256084
Adcy1	ENSRNOG00000059479	1.733986265	0.001826975	0.026903853
Rgn	ENSRNOG00000007949	1.733734681	0.005975775	0.060898262
Treml4	ENSRNOG00000042360	1.729938074	0.048339834	NA
S100a1	ENSRNOG00000012410	1.72968025	0.000309361	0.007066914
Fgf6	ENSRNOG00000053662	1.72428493	0.010911382	NA
Nr1i2	ENSRNOG00000002906	1.705576089	0.039911538	0.194302343
Nrtn	ENSRNOG00000048651	1.701976935	0.007203139	0.06857712
Bsn	ENSRNOG00000030714	1.693878108	0.04182597	0.198685187
Sec14l3	ENSRNOG00000004555	1.687234794	0.005859391	0.05997984
Neu3	ENSRNOG00000018106	1.686138266	1.49E-06	9.22E-05
Oxtr	ENSRNOG00000005806	1.685878379	0.000587438	0.011660885
Lrrc46	ENSRNOG00000009902	1.684933498	0.000405524	0.008750775
Spint1	ENSRNOG00000012811	1.682246721	0.046259999	0.208786436

Igfbp2	ENSRNOG00000016957	1.675062281	0.035681595	NA
Celsr2	ENSRNOG00000020058	1.674916703	0.000213679	0.00525927
Cacna2d4	ENSRNOG00000008031	1.674790126	0.04346251	0.202559655
Ppp1r1a	ENSRNOG00000036827	1.671263225	8.18E-06	0.000385095
Pkdrej	ENSRNOG00000029591	1.666921168	0.019582853	0.129819618
Frmd5	ENSRNOG00000016189	1.665408407	0.009339992	0.081249062
Kcnd3	ENSRNOG00000014686	1.649836353	0.047275618	0.211088481
Cyp2f4	ENSRNOG00000032805	1.644600745	0.028422562	0.161153912
Foxo6	ENSRNOG00000032639	1.644119572	2.90E-15	1.37E-12
Snph	ENSRNOG00000009588	1.634583264	0.00961453	0.082713622
Lrrc52	ENSRNOG00000004356	1.632524111	0.031780768	NA
Pacsin1	ENSRNOG00000054603	1.626951099	0.04520671	0.205978747
Acot1	ENSRNOG00000055221	1.626159589	0.047147907	NA
Lingo3	ENSRNOG00000032569	1.617617474	0.049786182	0.217823283
Atp8b5p	ENSRNOG00000021704	1.614885171	0.015569897	0.113222141
Myl3	ENSRNOG00000020955	1.609110887	0.006670649	0.06516986
Kcnip2	ENSRNOG00000018018	1.608955571	0.018033709	0.122676369
Lrrc27	ENSRNOG00000017538	1.605093473	0.045247357	0.206006377
B3gat2	ENSRNOG00000046852	1.599738495	0.042914055	0.201204026
Tex22	ENSRNOG00000028158	1.597047832	0.032752875	NA
Traip	ENSRNOG00000030101	1.595260825	0.026505773	0.154893461
Rnf43	ENSRNOG00000007606	1.589512801	0.001635816	0.024986289
Proser2	ENSRNOG00000045553	1.586695005	6.80E-06	0.000333618
Tnfrsf13b	ENSRNOG00000002304	1.579160573	0.012850119	0.099653982
Dusp26	ENSRNOG00000011518	1.575621072	3.04E-06	0.000169032
Stpg4	ENSRNOG00000040136	1.575272422	0.022290409	0.139248024
Fam241b	ENSRNOG00000000551	1.574324385	0.019070944	0.127521594
Rab6b	ENSRNOG00000009198	1.573392084	0.040872004	0.196691674
Arhgef4	ENSRNOG00000014035	1.572643157	0.015034922	0.110753705
Rorc	ENSRNOG00000020836	1.571661454	0.045212365	NA
Kcne2	ENSRNOG00000029811	1.568168182	0.00276903	0.035812095
Cacng8	ENSRNOG00000057848	1.566311419	0.037132913	0.186502507
Sctr	ENSRNOG00000049766	1.562412349	0.034885831	0.180734912
Dnah5	ENSRNOG00000048363	1.558745682	0.032375129	0.173500012
Actl10	ENSRNOG00000036864	1.552966439	0.009922922	0.084182615
Fzd9	ENSRNOG00000001452	1.552468786	0.020539981	0.133131501
Zfp385a	ENSRNOG00000036833	1.550974025	0.00150561	0.023434529
Msrb2	ENSRNOG00000016873	1.550151684	8.29E-06	0.000387997
Ndufa10l1	ENSRNOG00000062245	1.549095044	0.002381705	0.032071702
Tspy26	ENSRNOG00000026770	1.548300699	0.040878539	0.196691674
Mfrp	ENSRNOG00000039107	1.54688449	0.04973931	0.217706658
Neu2	ENSRNOG00000016962	1.546723788	0.007960517	0.073648961
Veph1	ENSRNOG00000012427	1.546670788	0.025762302	0.152028743
Pcdhb8	ENSRNOG00000020073	1.544623086	0.047339782	0.211272929
Fads6	ENSRNOG00000021380	1.540156404	0.001295267	0.021087008
Alkbh2	ENSRNOG00000028584	1.537242312	0.018978077	0.126955102
Mpv17l	ENSRNOG00000003285	1.536853402	0.0028595	0.036667497
Celsr1	ENSRNOG00000021285	1.535387785	0.01049594	0.087167772
Slc35g2	ENSRNOG00000015370	1.528840629	0.035466184	NA
Sspo	ENSRNOG00000025848	1.526621545	0.03272289	0.174776356

Mttp	ENSRNOG00000010655	1.523708697	0.004582017	0.050737614
Asxl3	ENSRNOG00000015383	1.518958048	0.020705974	0.133470862
Csdc2	ENSRNOG00000005332	1.510382696	0.015286296	0.111917525
Adam1b	ENSRNOG00000037801	1.509772158	0.000152983	0.004102376
Lgr6	ENSRNOG00000005093	1.509166168	0.013693998	0.103972946
Cntfr	ENSRNOG00000047307	1.508335766	0.004735796	0.051899077
Atp6v1b1	ENSRNOG00000013573	1.508332045	0.021155752	0.135029336
Alx4	ENSRNOG00000000008	1.507297271	0.038152504	0.189451337
Fgfr2	ENSRNOG00000016374	1.507284924	5.54E-05	0.00181958
Kash5	ENSRNOG00000025378	1.507067565	0.000283657	0.006566671
Myom3	ENSRNOG00000032994	1.502766736	0.007715483	0.071851299
Plch2	ENSRNOG00000014226	1.499949929	0.038622608	0.190666315
Ltb	ENSRNOG00000070531	1.499677545	0.011035249	0.089839098
Pkd2l1	ENSRNOG00000012591	1.498686823	0.042535639	0.200517654
Ano2	ENSRNOG00000023561	1.497026322	0.034277327	0.179088112
Fzd10	ENSRNOG00000022784	1.496318945	0.021693562	0.136836313
Wscd1	ENSRNOG00000007869	1.494095302	0.001469556	0.023080318
Mfsd4b2	ENSRNOG00000042918	1.492658242	0.041987101	NA
Slc38a1	ENSRNOG00000005291	1.486696247	0.000815392	0.014823578
Smtnl1	ENSRNOG00000007516	1.485428489	7.17E-06	0.00034643
Acox2	ENSRNOG00000007378	1.484773459	0.000530708	0.010752187
Sez6	ENSRNOG00000009350	1.483697928	0.028404513	0.161153912
Pkd1l3	ENSRNOG00000052310	1.482272416	0.021342241	0.135719233
Ush1c	ENSRNOG00000021149	1.48209533	0.000767653	0.01418747
Dbndd1	ENSRNOG00000026974	1.479829265	0.020534342	0.133131501
Gpat2	ENSRNOG00000013906	1.476392054	0.038147164	0.189451337
Syngr4	ENSRNOG00000021093	1.47294303	0.041163662	0.197343617
Tmem74b	ENSRNOG00000064442	1.471957912	6.66E-05	0.002105645
Sc5d	ENSRNOG00000065944	1.468120227	6.72E-07	4.59E-05
Capn13	ENSRNOG00000032375	1.465009074	0.048469916	0.213926713
Gnao1	ENSRNOG00000019482	1.460751939	2.56E-06	0.000147449
Myrf	ENSRNOG00000028274	1.459990583	0.042165521	0.199486411
Foxp3	ENSRNOG00000011702	1.455855097	0.000160695	0.004256979
Tmem116	ENSRNOG00000027480	1.453390359	6.64E-07	4.56E-05
Bambi	ENSRNOG00000016066	1.452458119	0.000308212	0.00705132
Ehhadh	ENSRNOG00000001770	1.449886653	5.32E-05	0.001774227
Lrrn2	ENSRNOG00000009691	1.44901163	0.031828928	0.171471196
Nat8f4	ENSRNOG00000070875	1.448518569	0.033184384	0.176034289
Ddo	ENSRNOG00000000582	1.447946384	1.33E-06	8.49E-05
Nos2	ENSRNOG00000057443	1.446258581	0.010659513	0.088010182
L1cam	ENSRNOG00000061230	1.444444884	0.038605388	0.190666315
Ttll9	ENSRNOG00000008596	1.439128315	0.036109842	0.183373971
Klc3	ENSRNOG00000018101	1.438618365	0.031434313	0.170405916
Pim2	ENSRNOG00000008177	1.437199075	0.016163294	0.115126716
Ldhd	ENSRNOG00000019036	1.431587624	2.09E-07	1.68E-05
Srcin1	ENSRNOG00000011475	1.430237814	0.024735143	0.148563427
Epop	ENSRNOG00000048187	1.422876988	0.02499489	0.1492604
Ttc30a1	ENSRNOG00000052519	1.420627991	0.030959744	NA
Hspa1l	ENSRNOG00000047966	1.418045461	0.002769806	0.035812095
Bckdha	ENSRNOG00000020607	1.415802356	2.67E-06	0.000153588

B3gat1	ENSRNOG00000007142	1.4148761	0.045307027	0.206097366
Pcdh20	ENSRNOG00000013306	1.411710268	0.029865282	0.165823626
Mmp17	ENSRNOG00000023643	1.411377159	0.031904963	0.171762034
Sec14l5	ENSRNOG00000002917	1.409317686	0.000800413	0.014619499
Adhfe1	ENSRNOG00000007069	1.404261135	5.41E-11	1.07E-08
Krbox1	ENSRNOG00000063118	1.404171169	0.023842195	NA
Dusp14	ENSRNOG00000030091	1.40387814	6.37E-05	0.002026135
Lrrc71	ENSRNOG00000014937	1.400961878	0.019552505	0.129819618
Pitx2	ENSRNOG00000010681	1.398927156	0.015993163	0.114441917
Prr22	ENSRNOG00000047295	1.394612171	0.025449284	0.150588621
Lrrc74b	ENSRNOG00000001872	1.392754814	8.42E-05	0.002547199
Pm20d1	ENSRNOG00000039745	1.387726648	2.85E-07	2.21E-05
Mkrn3	ENSRNOG00000010172	1.386320712	0.031745453	0.171226848
Pdzd7	ENSRNOG00000032946	1.383128422	0.000205442	0.005134396
Gsta4	ENSRNOG00000030449	1.379315263	0.004513505	0.050337407
Kcnb1	ENSRNOG00000046949	1.376249938	4.96E-06	0.000254213
Pomc	ENSRNOG00000012686	1.372179012	0.030072125	0.166544602
Fam89b	ENSRNOG00000024239	1.371393705	8.01E-06	0.000377943
Epb41l4b	ENSRNOG00000056550	1.369515155	0.048840941	0.215016067
Aqp7	ENSRNOG00000009686	1.365759413	0.023909473	0.145284553
Topaz1	ENSRNOG00000025601	1.364022097	0.006682269	0.065190827
Dact2	ENSRNOG00000022921	1.362874445	0.000142605	0.003911595
Fer1l6	ENSRNOG00000025300	1.362502194	0.037892653	0.188676961
Ciart	ENSRNOG00000042717	1.361572072	0.027413351	0.157892793
C1qtnf12	ENSRNOG00000019864	1.360378829	0.048827811	0.215016067
Fuom	ENSRNOG00000018476	1.358848518	0.006942424	0.066849796
Prxl2a	ENSRNOG00000011140	1.357095032	3.27E-05	0.001199343
Kcnk4	ENSRNOG00000021140	1.355869473	0.035910574	0.182854236
Kcnh1	ENSRNOG00000003841	1.355728606	0.035275832	0.181445185
Crygd	ENSRNOG00000032219	1.354797219	0.023539876	0.144106591
Zfp536	ENSRNOG00000014163	1.354744098	0.002534767	0.033511771
Tenm2	ENSRNOG00000027341	1.353940638	0.028218061	0.160568808
Chst12	ENSRNOG00000001252	1.353199685	0.000214989	0.00528317
Diras3	ENSRNOG00000068695	1.352001669	0.003217979	0.03988553
Fam229a	ENSRNOG00000042787	1.348404647	0.035756148	0.182642627
Wnt9a	ENSRNOG00000003066	1.347735764	7.68E-06	0.000364788
Spa17	ENSRNOG00000010934	1.342940514	0.00011017	0.003155244
Acsl6	ENSRNOG00000026745	1.336531092	0.004228035	0.048152622
Fer1l5	ENSRNOG00000024201	1.330245374	0.039367582	0.192957112
Qrich2	ENSRNOG00000025254	1.329148563	0.047081915	0.210681663
Me3	ENSRNOG00000017311	1.325564935	0.000100904	0.002966201
Spns3	ENSRNOG00000015873	1.321315077	0.028083264	0.160152728
Smad9	ENSRNOG00000000091	1.320832992	0.042416179	0.200258395
Thnsl1	ENSRNOG00000033076	1.319489615	0.019480103	0.129479524
Lrrc73	ENSRNOG00000039856	1.316882413	0.04825587	0.213344626
Ptch2	ENSRNOG00000057616	1.315449222	0.044130909	0.204084167
Hpn	ENSRNOG00000021097	1.314255693	0.001047599	0.017896485
Crb1	ENSRNOG00000010903	1.311942007	0.023435709	0.143864594
Lhb	ENSRNOG00000047040	1.309868919	0.012098584	0.095683217
Osbp2	ENSRNOG00000019645	1.305856301	0.001270205	0.020784083

Bdh1	ENSRNOG00000001736	1.304034635	0.038279358	0.189873418
Syt14	ENSRNOG00000046139	1.303876874	0.001680227	0.025439066
Cmbl	ENSRNOG00000011260	1.303002006	0.005066324	0.054382245
Slc16a3	ENSRNOG00000036677	1.30067888	0.003930627	0.046031593
Fndc5	ENSRNOG00000030238	1.298852671	0.005525432	0.057621307
Svip	ENSRNOG00000037137	1.294900939	0.001780112	0.026413472
Gcat	ENSRNOG00000055408	1.291362602	0.000907406	0.01608349
Spata31d1	ENSRNOG00000027014	1.291199471	0.013078861	0.100925537
Ubald2	ENSRNOG00000066106	1.290871298	1.60E-05	0.000658062
Adam22	ENSRNOG00000042478	1.290471803	0.000309801	0.007066914
Timm23b	ENSRNOG00000031285	1.288146626	0.023218956	0.143339982
Lonrf2	ENSRNOG00000023312	1.287209085	0.001769885	0.026362155
Gucy2c	ENSRNOG00000009031	1.284004712	0.03574474	0.182642627
Cracd	ENSRNOG00000002139	1.283030447	0.041418856	0.197539122
Acss1	ENSRNOG00000007102	1.282739636	3.05E-06	0.000169226
Sdhaf3	ENSRNOG00000011283	1.282353125	0.001107754	0.018635583
Dgat2	ENSRNOG00000016573	1.282291124	7.75E-06	0.000366886
Osr2	ENSRNOG00000011136	1.275239067	0.010375407	0.086350877
Dnaja4	ENSRNOG00000012106	1.275164528	0.000219507	0.005342284
Tdrkh	ENSRNOG00000020860	1.275054107	0.048639151	NA
Tas1r1	ENSRNOG00000009708	1.273047994	0.021444916	0.136025567
Acsm5	ENSRNOG00000031211	1.266269081	0.034798266	0.180520548
Cryga	ENSRNOG00000046343	1.265116688	0.044486792	0.204618632
Nxnl1	ENSRNOG00000042658	1.261258194	0.022024467	0.138307617
Zdhhc23	ENSRNOG00000060348	1.260804018	0.047779392	0.212201518
Tmie	ENSRNOG00000027799	1.259057612	0.021458037	0.136025567
Smpdl3b	ENSRNOG00000042326	1.257301168	0.001100472	0.018571844
Alpl	ENSRNOG00000013954	1.256311571	0.033245193	0.176176909
Hs3st5	ENSRNOG00000000605	1.254698301	0.002839758	0.036474381
Clca5	ENSRNOG00000013334	1.251828407	0.020029891	0.131009948
Ybey	ENSRNOG00000021365	1.245863414	1.19E-05	0.000519442
Lgi2	ENSRNOG00000066201	1.245238219	0.006526055	0.06439261
Aifm3	ENSRNOG00000037957	1.243541774	0.037031335	0.186112323
Trpc7	ENSRNOG00000012727	1.24276808	0.040319632	0.195541157
Sap18	ENSRNOG00000020771	1.239945568	0.030226049	0.166816097
Zfp853	ENSRNOG00000030579	1.237683208	0.0097173	0.083230094
Frat1	ENSRNOG00000067076	1.23543324	7.46E-05	0.002297008
Gpr162	ENSRNOG00000016143	1.232013058	0.028037053	0.160020323
Ecsit	ENSRNOG00000014128	1.231848341	0.000179052	0.004618601
Aldh1a3	ENSRNOG00000052070	1.231071563	0.021061322	0.134808974
Acat1	ENSRNOG00000007862	1.230187615	0.000568729	0.011374577
Gstk1	ENSRNOG00000016484	1.230180576	0.002715683	0.035416976
Smco1	ENSRNOG00000024960	1.228567063	0.007298867	0.069002638
Stard10	ENSRNOG00000019491	1.227047401	0.000151211	0.004075896
Flywch2	ENSRNOG00000004461	1.226457367	0.006892997	0.066538352
Lef1	ENSRNOG00000010121	1.226311317	0.035913605	0.182854236
Cyp2j10	ENSRNOG00000042224	1.225337311	0.010997177	0.089723367
Sema7a	ENSRNOG00000007687	1.224709798	0.000352119	0.007814837
Hpdl	ENSRNOG00000018143	1.216927232	0.014606123	0.108296107
Cys1	ENSRNOG00000058891	1.216364057	0.003448075	0.041969539

Sh2b2	ENSRNOG00000001425	1.213059993	7.25E-05	0.002248565
Ccr9	ENSRNOG00000006311	1.208341483	0.009780701	0.083542691
Atp13a5	ENSRNOG00000024127	1.208127183	0.019742715	0.130318069
Neto2	ENSRNOG00000016245	1.205640324	0.047909198	0.212535681
Tmem177	ENSRNOG00000025484	1.205593517	0.000145328	0.00396533
Sall4	ENSRNOG00000050035	1.205199852	0.007971808	0.073665937
Klhl10	ENSRNOG00000016749	1.204232917	0.017201794	0.118842152
Hcn2	ENSRNOG00000008831	1.204074331	0.000772469	0.014242682
Nr0b2	ENSRNOG00000007229	1.202585069	0.032329962	0.173423302
Ivd	ENSRNOG00000009421	1.202524678	0.000395859	0.008613798
Abcd2	ENSRNOG00000015538	1.200806019	0.001970936	0.0283204
Dnajc22	ENSRNOG00000053498	1.197634731	0.008918126	0.078938089
Cspg4b	ENSRNOG00000039582	1.196349592	0.001560041	0.02416048
Fkbp4	ENSRNOG00000006444	1.195923358	0.000106227	0.003062283
Aco2	ENSRNOG00000024128	1.195357833	0.001814439	0.026820649
Wnk2	ENSRNOG00000016684	1.194721456	4.42E-06	0.00023241
Ccdc158	ENSRNOG00000002204	1.190513458	0.000441031	0.009367139
Zc2hc1c	ENSRNOG00000027115	1.189402325	0.000165225	0.00433367
Garem2	ENSRNOG00000048004	1.189188965	0.002248164	0.030751888
Asb18	ENSRNOG00000019557	1.188625675	0.002716969	0.035416976
Pc	ENSRNOG00000019372	1.187134836	3.18E-07	2.41E-05
Them6	ENSRNOG00000005929	1.184972896	0.013362374	0.102527019
Echs1	ENSRNOG00000064647	1.183116801	0.003398869	0.041663551
Adss1	ENSRNOG00000046763	1.182374329	0.000382132	0.00837358
Chchd10	ENSRNOG00000028356	1.17967747	1.14E-05	0.000505271
Nudt4	ENSRNOG00000009094	1.177959171	0.005885849	0.060171604
Cbarp	ENSRNOG00000024349	1.176073912	0.020610602	0.133131501
Fam151a	ENSRNOG00000007799	1.16845702	0.00124559	0.020449203
Rassf10	ENSRNOG00000064154	1.167875905	0.016773849	0.117191288
Epm2a	ENSRNOG00000040242	1.16783761	0.002020729	0.028810284
Ccrl2	ENSRNOG00000033234	1.164587221	0.020124818	0.131410169
Wfdc1	ENSRNOG00000015904	1.162289628	0.001068339	0.018139061
Peli3	ENSRNOG00000019950	1.157932011	0.033365382	0.176491006
Shc2	ENSRNOG00000008030	1.157853608	4.67E-05	0.001607118
Slc1a3	ENSRNOG00000016163	1.156664247	0.023613787	0.144332207
Ddit4l	ENSRNOG00000010047	1.154834736	0.008248691	0.075051231
Slc37a1	ENSRNOG00000001169	1.154610898	8.45E-07	5.65E-05
Wfikkn2	ENSRNOG00000002831	1.154155349	0.009446643	0.081765947
Bcat2	ENSRNOG00000020956	1.153762598	4.31E-08	4.20E-06
Sdha	ENSRNOG00000013331	1.150119148	0.001629583	0.024959086
Nr1d1	ENSRNOG00000009329	1.149079112	0.002326385	0.031544884
Tmem253	ENSRNOG00000039489	1.149021708	0.030018754	0.166415849
Eif4e3	ENSRNOG00000010301	1.148416439	0.000833164	0.015076298
Selenbp1	ENSRNOG00000047158	1.147812047	0.00029074	0.006700785
Eef2kmt	ENSRNOG00000002876	1.14650454	0.016848224	0.117447578
Tspan1	ENSRNOG00000023320	1.145191342	0.016438675	0.116046468
Mrpl42	ENSRNOG00000042740	1.143691096	0.001572139	0.024323659
Asb16	ENSRNOG00000036794	1.13942713	0.003594715	0.043116056
Slc45a3	ENSRNOG00000007591	1.139092344	0.030194584	0.166760586
Atp5f1b	ENSRNOG00000002840	1.138813955	0.012833754	0.09962625

Odf3	ENSRNOG00000012858	1.136287899	0.024702339	0.148423618
Ky	ENSRNOG00000008210	1.132449092	0.032326968	0.173423302
Tssk6	ENSRNOG00000020599	1.129892208	0.04587589	0.208077543
Polr2j	ENSRNOG00000001430	1.129562164	0.000140623	0.003864023
Fabp3	ENSRNOG00000012879	1.127193753	0.001698423	0.025566603
Iqcg	ENSRNOG00000026420	1.121416599	0.012684393	0.098808964
Adamts8	ENSRNOG00000005574	1.120209067	0.040314435	0.195541157
Smtnl2	ENSRNOG00000015157	1.11909888	0.002243467	0.030744173
Asb4	ENSRNOG00000009197	1.118510072	0.000792341	0.01450608
Sptbn2	ENSRNOG00000058842	1.116183667	0.035617393	0.182317684
Rfx2	ENSRNOG00000045846	1.114949569	0.001797334	0.02659303
Rhpn1	ENSRNOG00000007597	1.11415125	0.026002954	0.153051011
Esm1	ENSRNOG00000010797	1.111365593	0.031316001	0.170060404
Tnni1	ENSRNOG00000009073	1.110372897	0.035954139	0.18288132
Cbs	ENSRNOG00000029528	1.110334299	0.033459811	0.17671317
Hint2	ENSRNOG00000015866	1.109800032	0.000678457	0.013001674
Gatd3a	ENSRNOG00000001211	1.104874732	0.000502142	0.010313746
Trpm1	ENSRNOG00000015829	1.104542252	0.004325479	0.048897314
Prima1	ENSRNOG00000008915	1.10197658	0.047039445	0.210681663
Aldoc	ENSRNOG00000011452	1.099903002	0.039075869	0.191990552
Gls2	ENSRNOG00000031612	1.099197898	0.02718496	0.157093562
Itgad	ENSRNOG00000068399	1.097538196	0.01968049	0.130131458
Pink1	ENSRNOG00000015385	1.097245689	0.002084379	0.029309224
Hfe	ENSRNOG00000016967	1.096272413	0.004307202	0.048804516
Fgf12	ENSRNOG00000001931	1.095995251	0.046076608	0.208562914
Cfap126	ENSRNOG00000034221	1.093544586	0.038274006	0.189873418
Tmem248	ENSRNOG00000000893	1.093205297	0.00559111	0.057992791
Pebp1	ENSRNOG00000001136	1.091982472	0.00523436	0.055401928
Vdac1	ENSRNOG00000006375	1.091692879	0.005865115	0.059999013
Syngr1	ENSRNOG00000017108	1.091105755	0.001475834	0.023109046
Unc119	ENSRNOG00000011060	1.088093188	0.002631205	0.034565069
Ccbe1	ENSRNOG00000062986	1.08570145	0.000399578	0.008670502
Satb1	ENSRNOG00000012942	1.084417409	0.0001814	0.004663719
Espl1	ENSRNOG00000012835	1.07962903	3.20E-07	2.41E-05
Ldhb	ENSRNOG00000013000	1.078426718	0.030514493	0.167648975
Lrrc3b	ENSRNOG00000005857	1.077815265	0.007789196	0.072321621
Mlf1	ENSRNOG00000012827	1.076525832	0.010190788	0.085470934
Cpa1	ENSRNOG00000010725	1.076275843	0.000481802	0.010002647
Atp1b2	ENSRNOG00000011227	1.075358388	0.009559805	0.082424878
Acads	ENSRNOG00000001177	1.075341574	0.003002476	0.038000466
Glyctk	ENSRNOG00000046307	1.075047484	0.002646203	0.034703576
Sar1b	ENSRNOG00000004820	1.073417622	0.001129515	0.018942775
Aldh6a1	ENSRNOG00000011419	1.073329888	0.004142558	0.047631775
Hlf	ENSRNOG00000067479	1.073135032	0.008304608	0.075224296
Ppp1r26	ENSRNOG00000027193	1.071791759	2.31E-05	0.000887574
Astn1	ENSRNOG00000005667	1.071708589	0.031270752	0.16993314
Perm1	ENSRNOG00000020244	1.071440859	0.009877475	0.084009932
Apln	ENSRNOG00000003984	1.070329093	0.039940776	0.19434019
Lamtor1	ENSRNOG00000004319	1.067730037	0.005583937	0.057992791
Txnrd2	ENSRNOG00000001890	1.066984971	1.25E-05	0.000541543

Timm8b	ENSRNOG00000009888	1.064758148	0.019788148	0.130318069
Vwa3b	ENSRNOG00000023566	1.064616159	0.00682112	0.066090205
Esrp2	ENSRNOG00000023177	1.06023908	0.040122129	0.194857472
Zfp358	ENSRNOG00000000974	1.05923083	0.006699066	0.065224534
Pdk2	ENSRNOG00000004172	1.058638741	0.004514055	0.050337407
Tmcc2	ENSRNOG00000000033	1.058208376	0.001589724	0.024474203
Bmp6	ENSRNOG00000013717	1.057248162	5.42E-05	0.001791724
Apobec2	ENSRNOG00000012303	1.056764622	0.028888272	0.16289514
Tle5	ENSRNOG00000052025	1.056397753	0.002113564	0.02948605
Cox5b	ENSRNOG00000016660	1.053563891	0.000605822	0.011947735
Akr7a2	ENSRNOG00000017780	1.0531379	0.000792262	0.01450608
Mettl7a	ENSRNOG00000001376	1.052788719	0.000335696	0.007547111
Muc6	ENSRNOG00000056817	1.050885656	0.039371673	0.192957112
Zfp691	ENSRNOG00000007398	1.048980568	0.000935021	0.016423488
Exog	ENSRNOG00000014801	1.048676921	0.02405188	0.145640552
Acyp2	ENSRNOG00000042419	1.048497596	0.020248856	0.131943609
Lrrc14b	ENSRNOG00000028357	1.046242098	0.003787144	0.044767604
Llgl2	ENSRNOG00000004834	1.044590757	0.004533836	0.050455117
Ajm1	ENSRNOG00000033863	1.044327734	2.16E-05	0.000846475
Rhbdl3	ENSRNOG00000005515	1.043140841	0.022116994	0.138724271
Gstm7	ENSRNOG00000018937	1.043116393	0.000819278	0.01484226
Zfp365	ENSRNOG00000000638	1.043013458	0.027869032	0.15959397
Mdh1	ENSRNOG00000008103	1.04202447	0.010844957	0.089116258
Acadm	ENSRNOG00000009845	1.041670928	0.014751572	0.109115553
Wnt2b	ENSRNOG00000014385	1.037982729	0.00220002	0.030386796
Cdnf	ENSRNOG00000026493	1.035748702	0.001381078	0.021978755
Hjv	ENSRNOG00000021200	1.034700097	0.029598825	0.165464548
Pfkfb1	ENSRNOG00000000165	1.033014849	0.000995377	0.017250255
Pccb	ENSRNOG00000015869	1.032514336	0.000332577	0.007487798
Ndufa9	ENSRNOG00000061684	1.031741032	0.005187448	0.055167531
Aven	ENSRNOG00000006419	1.030647548	0.000217446	0.005315026
Ttc30a	ENSRNOG00000069321	1.030112411	0.009207648	0.080457179
Atp1a4	ENSRNOG00000032378	1.0300069	0.002773893	0.035835196
Hadh	ENSRNOG00000010697	1.02843032	0.010736704	0.088319875
Fhl3	ENSRNOG00000007541	1.026346401	0.005146679	0.054883818
Tmem143	ENSRNOG00000021096	1.026182048	0.001066295	0.01813632
Crat	ENSRNOG00000018145	1.024087733	0.001515129	0.023511666
Mapk11	ENSRNOG00000006984	1.023579308	0.046833484	0.210460249
Homer2	ENSRNOG00000061450	1.023100424	0.003413836	0.041689072
Plaat1	ENSRNOG00000001711	1.021333215	0.024822288	0.148914612
Suox	ENSRNOG00000005987	1.020422841	0.000303544	0.006954725
Lrrc20	ENSRNOG00000000560	1.019638265	0.004588298	0.050771087
Pfkm	ENSRNOG00000057988	1.019314167	0.0021052	0.029442563
Net1	ENSRNOG00000017765	1.018785954	0.008470071	0.076042901
Fdft1	ENSRNOG00000021314	1.017735801	0.002055968	0.029055663
Ndufb4	ENSRNOG00000002721	1.017626292	0.010218161	0.085498899
Cth	ENSRNOG00000010658	1.015421234	0.015845023	0.114025617
Tmem50b	ENSRNOG00000002028	1.014261184	0.009693904	0.083158921
Uqcrc1	ENSRNOG00000032134	1.014109534	0.005713939	0.058916724
Grhpr	ENSRNOG00000012794	1.014013021	0.001835118	0.026983494

Cystm1	ENSRNOG00000018775	1.013538233	0.001325787	0.02147169
Tas1r3	ENSRNOG00000019589	1.01236854	0.012823526	0.099596477
Fbxl18	ENSRNOG00000001117	1.003455501	0.000470171	0.00984579
Znf48	ENSRNOG00000017837	1.000505892	4.51E-06	0.000235113
Tuba4a	ENSRNOG00000003597	1.000322003	0.01668842	0.116831018
Fam131b	ENSRNOG00000017149	0.999399566	0.004076125	0.047107019
Phyh	ENSRNOG00000018044	0.999131673	0.008724793	0.077654165
Trpc2	ENSRNOG00000020188	0.998105691	0.030908436	0.168926504
Ephx2	ENSRNOG00000017286	0.997810888	0.016735403	0.117027637
Mn1	ENSRNOG00000027489	0.995881743	0.001792447	0.026548876
Zfp93	ENSRNOG00000012762	0.994962504	0.000901927	0.01604113
Hsd17b10	ENSRNOG00000003049	0.991177073	0.009925768	0.084182615
Zswim1	ENSRNOG00000015735	0.990691034	0.000895444	0.01596226
Tcap	ENSRNOG00000068106	0.987210263	0.003082283	0.038665036
Synm	ENSRNOG00000014030	0.987097584	0.001229973	0.020278285
Lzts3	ENSRNOG00000021231	0.986309447	0.019580019	0.129819618
Tmem147	ENSRNOG00000021006	0.986294976	0.005663741	0.058528796
Ube3d	ENSRNOG00000010802	0.985819104	0.001471291	0.023084304
Frs3	ENSRNOG00000014328	0.983920533	0.000482156	0.010002647
Mrpl16	ENSRNOG00000021005	0.983710183	0.027047779	0.15665591
Lgalsl	ENSRNOG00000005464	0.983338088	0.021057068	0.134808974
Pmpca	ENSRNOG00000026775	0.982947871	0.002029827	0.028886818
Cabcoco1	ENSRNOG00000000634	0.981525401	0.000688399	0.013111561
Calm3	ENSRNOG00000016770	0.978827118	0.007404535	0.069664701
Tmem141	ENSRNOG00000028254	0.97709476	0.004993671	0.053841794
Dglucy	ENSRNOG00000004442	0.974696364	6.81E-06	0.000333618
Phkg1	ENSRNOG00000000920	0.971644334	0.005286838	0.055692318
Mamdc4	ENSRNOG00000016584	0.969688793	0.036752123	0.185607154
Ppfia3	ENSRNOG00000020731	0.969631158	0.017191814	0.118832502
Pde4a	ENSRNOG00000020828	0.96945299	7.40E-05	0.002284485
Bhlhe41	ENSRNOG00000048961	0.968419225	0.025824054	0.152228057
Lrrc38	ENSRNOG00000015264	0.96811851	0.016300184	0.115539975
Kif1c	ENSRNOG00000031364	0.966976776	0.001880928	0.027439002
Osgin1	ENSRNOG00000014948	0.966584103	7.73E-05	0.002362501
Slc35g1	ENSRNOG00000029740	0.966393158	0.038484845	0.190407711
Asb12	ENSRNOG00000004197	0.965714323	0.048828434	0.215016067
Gfer	ENSRNOG00000013370	0.964154504	0.001973769	0.0283204
Fanca	ENSRNOG00000016706	0.963603896	0.011615256	0.093185212
Timm23	ENSRNOG00000019811	0.962842374	0.009745314	0.08336163
Pomgnt2	ENSRNOG00000019492	0.962226824	0.002535968	0.033511771
Tmem8b	ENSRNOG00000015664	0.961148089	0.002074153	0.029191781
Rabgef1	ENSRNOG00000000895	0.960458024	0.001340883	0.021626248
Nat14	ENSRNOG00000038622	0.960355054	0.001348386	0.021672562
Fkbpl	ENSRNOG00000000432	0.959581548	0.004681775	0.051512755
Tmem132l	ENSRNOG00000038406	0.958463912	0.011957132	0.094805152
Dhrs4	ENSRNOG00000018239	0.95810473	3.96E-05	0.001423923
Ndufs7	ENSRNOG00000024568	0.957267126	0.005007096	0.053911925
Mcee	ENSRNOG00000016327	0.957225628	0.003692107	0.043910712
Tlcd5	ENSRNOG00000021793	0.95494519	0.014565674	0.108150374
Mif	ENSRNOG00000006589	0.954729395	0.000439918	0.009363279

Mylk2	ENSRNOG00000008235	0.952720965	0.000626909	0.012193802
Ppara	ENSRNOG00000021463	0.952703156	0.000363364	0.008041494
Mrpl14	ENSRNOG00000019734	0.951997307	0.007106619	0.067961643
Ech1	ENSRNOG00000020308	0.950927493	0.001726624	0.025841304
B3gnt8	ENSRNOG00000068024	0.949173434	0.009843827	0.08385779
Acaa2	ENSRNOG00000013766	0.948511802	0.013492166	0.102992627
Zfp180	ENSRNOG00000029336	0.945593283	0.001146542	0.019145897
Foxo4	ENSRNOG00000033316	0.944134823	0.014303689	0.106786393
Gorasp1	ENSRNOG00000018047	0.942473384	0.000278271	0.006488621
Tuba8	ENSRNOG00000048169	0.940196013	0.008102297	0.074167911
Atp5f1e	ENSRNOG00000049912	0.938928115	0.03260643	0.174355661
Samd13	ENSRNOG00000016432	0.938306375	0.046879574	0.210552556
Sall2	ENSRNOG00000013287	0.938004698	0.015370384	0.112282298
Actn3	ENSRNOG00000019745	0.937673972	0.014509538	0.107805738
Pars2	ENSRNOG00000007327	0.935207519	0.006366547	0.063507944
Klf16	ENSRNOG00000033694	0.934236664	0.020544486	0.133131501
Clpp	ENSRNOG00000047052	0.933015747	0.000221049	0.005364407
Ntf4	ENSRNOG00000020783	0.932491214	0.015758234	0.113732773
Adam1a	ENSRNOG00000001347	0.931733248	0.001081585	0.018336342
Cyp26b1	ENSRNOG00000015076	0.931682217	0.046592786	0.209680995
Pigb	ENSRNOG00000059622	0.930986921	0.01338557	0.102581002
Tcea3	ENSRNOG00000011794	0.930748021	0.00081059	0.014753499
Gm35549	ENSRNOG00000062665	0.929660013	0.028419165	0.161153912
Ndufa11	ENSRNOG00000048320	0.929655051	0.007055245	0.067685168
Trarg1	ENSRNOG00000022239	0.929163229	0.028300843	0.160891089
Zfp579	ENSRNOG00000016608	0.925991158	0.020302471	0.132142294
Ilrun	ENSRNOG00000038883	0.924065501	0.017322361	0.119364169
Ankrd24	ENSRNOG00000006759	0.923961434	0.010215267	0.085498899
Clcn4	ENSRNOG00000003533	0.923435369	0.008029761	0.073851044
Slc39a5	ENSRNOG00000033361	0.923229379	0.005302356	0.055780357
Mypop	ENSRNOG00000014132	0.922095392	0.018566534	0.125195144
Sbk1	ENSRNOG00000067387	0.921869535	5.56E-05	0.0018235
Dhrs11	ENSRNOG00000027891	0.921637486	0.009452452	0.081770795
Mrpl54	ENSRNOG00000020464	0.9213503	0.016516952	0.116336808
Bckdhb	ENSRNOG00000009928	0.920328037	0.006231565	0.062556564
P2ry2	ENSRNOG00000019283	0.920288137	0.002817124	0.036273385
Nop10	ENSRNOG00000005184	0.91904265	0.006881587	0.06651062
Nfkbib	ENSRNOG00000020063	0.918289259	1.44E-05	0.000611605
C2cd4d	ENSRNOG00000020832	0.918210782	0.011223804	0.090934405
Bend3	ENSRNOG00000046505	0.918110288	0.000985668	0.017158324
St3gal6	ENSRNOG00000001653	0.91702922	0.01633513	0.115682422
Atp2a1	ENSRNOG00000047124	0.916561507	0.030086386	0.166544602
Fitm2	ENSRNOG00000027434	0.91628093	0.037892825	0.188676961
Pals2	ENSRNOG00000021579	0.915828373	0.000516501	0.010546641
Mxi1	ENSRNOG00000034078	0.914918147	0.005642585	0.058374155
Ptpn3	ENSRNOG00000011425	0.91490395	0.012943076	0.100175422
Rxrg	ENSRNOG00000004537	0.914648289	0.017176832	0.118781643
Wipf3	ENSRNOG00000009571	0.913942026	0.0043802	0.049281567
Sys1	ENSRNOG00000014480	0.913931581	0.001342371	0.021627856
Alpk3	ENSRNOG00000011659	0.913851665	0.000916707	0.016211462

Gys1	ENSRNOG00000020812	0.910952434	0.006192031	0.062374932
Nrap	ENSRNOG00000016714	0.909106379	0.000402303	0.008705394
Akr1b1	ENSRNOG00000009513	0.907610118	0.010030086	0.084698503
Nsun7	ENSRNOG00000025494	0.907403451	0.022281525	0.139248024
Klhl21	ENSRNOG00000003334	0.906859824	0.009095311	0.079743923
Atp1a2	ENSRNOG00000007290	0.906384735	0.005808483	0.059615391
Ndufa10	ENSRNOG00000016470	0.90528677	0.006688037	0.065206268
Rnf5	ENSRNOG00000000438	0.904955761	0.014190261	0.106568425
Gpd1	ENSRNOG00000056457	0.902119331	0.025430028	0.150566894
Per3	ENSRNOG00000018413	0.9020868	0.01190918	0.094569329
Clpb	ENSRNOG00000019693	0.901153006	0.000193839	0.004918587
Pygm	ENSRNOG00000021090	0.901044889	0.014924574	0.110149152
Ndufs6l1	ENSRNOG00000062531	0.900620586	0.012009258	0.09517001
Map3k10	ENSRNOG00000023521	0.900502234	0.001025536	0.017631971
Zfp106	ENSRNOG00000052583	0.899071159	0.02479679	0.148820725
Ndufb11	ENSRNOG00000008329	0.899045018	0.020598899	0.133131501
Dynll2	ENSRNOG00000008921	0.898972521	0.006191148	0.062374932
Coq9	ENSRNOG00000016190	0.898780075	0.012459036	0.097640321
Rida	ENSRNOG00000005437	0.896248112	0.028039505	0.160020323
Tusc2	ENSRNOG00000021528	0.894202747	0.024021251	0.145567199
Vgll2	ENSRNOG00000000405	0.892227065	0.048539681	0.214113315
Inka1	ENSRNOG00000019363	0.890461216	0.019965554	0.130759427
Rpusd1	ENSRNOG00000023639	0.890023529	0.008935506	0.079009749
Ckmt2	ENSRNOG00000055450	0.889930335	0.031289043	0.169973255
Narf	ENSRNOG00000036664	0.889404335	0.004190207	0.047967253
Zfp334	ENSRNOG00000019099	0.889395072	0.047065444	0.210681663
Chrdl2	ENSRNOG00000018394	0.888801617	0.009351978	0.081290979
Rab1b	ENSRNOG00000070897	0.888639209	0.008204001	0.074710083
Wdcp	ENSRNOG00000049989	0.887662342	0.013804104	0.104362751
Nek3	ENSRNOG00000012757	0.88725309	0.003014626	0.038123276
Atp5me	ENSRNOG00000000064	0.885744215	0.004010596	0.046630663
Shld1	ENSRNOG00000021268	0.884868979	0.003654285	0.043560647
Mdh2	ENSRNOG00000001440	0.884132628	0.013139401	0.101292363
Ndufv1	ENSRNOG00000018117	0.883407337	0.006618168	0.064890531
Ghitm	ENSRNOG00000013961	0.883172735	0.023489143	0.144007339
Prdx3	ENSRNOG00000010958	0.88284868	0.03898265	0.191713916
Oxa1l	ENSRNOG00000009713	0.882191592	0.000770177	0.014217241
Bap1	ENSRNOG00000019097	0.882189276	0.002637105	0.034613394
Nt5c1a	ENSRNOG00000068555	0.881119123	0.048535977	0.214113315
Rap1gap2	ENSRNOG00000002652	0.879594106	0.034078171	0.178406553
Efcab2	ENSRNOG00000042201	0.87803939	0.023685602	0.144600969
Asb11	ENSRNOG00000003452	0.877812248	0.021036771	0.134766815
Klhl22	ENSRNOG00000001878	0.875999221	0.000870504	0.015617769
Etfa	ENSRNOG00000015233	0.874270161	0.014972496	0.11039824
Ndufs8	ENSRNOG00000017446	0.874211239	0.023318761	0.143651291
Ddx23	ENSRNOG00000060154	0.873207497	1.38E-05	0.000595314
Aldh9a1	ENSRNOG00000004027	0.873089235	0.002977543	0.037807762
Gid4	ENSRNOG00000003847	0.873006121	0.044684689	0.204881533
Kyat3	ENSRNOG00000043426	0.872160843	0.005902709	0.060304402
Ddah1	ENSRNOG00000014613	0.870898723	0.034582658	0.180074202

Hps6	ENSRNOG00000018433	0.87089211	0.011274562	0.091203364
Idh3b	ENSRNOG00000007316	0.86868378	0.011135469	0.090548335
Kcna7	ENSRNOG00000020779	0.868329015	0.041341276	0.197380187
Gid4	ENSRNOG00000069998	0.8672809	0.024345842	0.146734318
Cox6b1	ENSRNOG00000024309	0.866555885	0.00617829	0.062374932
Clybl	ENSRNOG00000014075	0.866164784	0.031125932	0.169500878
Fam220a	ENSRNOG00000024605	0.865927429	0.006282196	0.062949055
Idh3g	ENSRNOG00000055572	0.865840496	0.005992685	0.060943884
Pla2g12a	ENSRNOG00000057470	0.865458451	0.003629438	0.04337182
Art1	ENSRNOG00000020251	0.865414992	0.024978692	0.1492604
Cyp27a1	ENSRNOG00000017188	0.865351346	0.003868559	0.045454114
Dusp8	ENSRNOG00000029394	0.864805642	0.006030836	0.061172154
Fn3k	ENSRNOG00000036659	0.864149787	0.024693444	0.148423618
Trappc6b	ENSRNOG00000004103	0.863145871	0.010814091	0.08890952
Ybx1	ENSRNOG00000023786	0.862858864	0.003777808	0.044691153
Mettl27	ENSRNOG00000046700	0.860565422	0.046025064	0.208390148
Dnajb12	ENSRNOG00000030408	0.860474044	0.00685111	0.066257164
Atp5pb	ENSRNOG00000064742	0.860194791	0.029973614	0.166300768
Eral1	ENSRNOG00000010673	0.85989926	0.007342007	0.069284355
Klhl33	ENSRNOG00000025868	0.859897312	0.01065788	0.088010182
Repin1	ENSRNOG00000008239	0.858914937	0.000615919	0.012041412
Magix	ENSRNOG00000010092	0.856665323	0.019961336	0.130759427
Mlycd	ENSRNOG00000014522	0.856153992	0.009844409	0.08385779
Mrps18b	ENSRNOG00000000804	0.855299185	0.001681787	0.025439066
Nxpe2	ENSRNOG00000027847	0.855249571	0.043177829	0.202015187
Ubxn10	ENSRNOG00000027731	0.855156682	0.018660335	0.125414757
Smim12	ENSRNOG00000014352	0.85505232	0.000195534	0.004937273
Fen1	ENSRNOG00000020531	0.854705968	0.000991023	0.017213082
Arhgap44	ENSRNOG00000003603	0.85398074	0.001236403	0.020341248
Dcun1d2	ENSRNOG00000019473	0.853320246	0.024959637	0.1492604
D2hgdh	ENSRNOG00000019012	0.852768505	0.000242315	0.005790277
Dbt	ENSRNOG00000015029	0.852561078	0.012883928	0.099816805
Mrps36	ENSRNOG00000061213	0.851923319	0.03948589	0.193273695
Sucla2	ENSRNOG00000017481	0.851909547	0.02666154	0.155284783
Map2k6	ENSRNOG00000004437	0.851872515	0.03002535	0.166415849
Eml1	ENSRNOG00000043143	0.851345949	0.006435702	0.063865082
Snrpn	ENSRNOG00000054391	0.850488631	0.028424323	0.161153912
Gcnt7	ENSRNOG00000063099	0.850305394	0.037217381	0.186746151
Ndufa8	ENSRNOG00000005668	0.849986731	0.020702745	0.133470862
Por	ENSRNOG00000001442	0.848980081	4.34E-08	4.20E-06
Acsl1	ENSRNOG00000010633	0.84878903	0.012997526	0.10044715
Fundc2	ENSRNOG00000024066	0.84827802	0.03562099	0.182317684
Klhl36	ENSRNOG00000016422	0.848076896	0.047099116	0.210681663
Zfp13	ENSRNOG00000003455	0.847357627	0.02766011	0.158844273
Timm17a	ENSRNOG00000007040	0.847349765	0.028027556	0.160020323
Dgcr6	ENSRNOG00000001880	0.845115078	0.002032089	0.028886818
Kctd15	ENSRNOG00000021137	0.844568687	0.000614157	0.012040824
Mpc2	ENSRNOG00000003150	0.844283951	0.030737754	0.168269221
Mri1	ENSRNOG00000026211	0.84414312	6.66E-06	0.0003296
Twf2	ENSRNOG00000048915	0.844118761	0.010300186	0.085862435

Coil	ENSRNOG00000000244	0.843442335	0.000118458	0.003355587
Thrb	ENSRNOG00000006649	0.842986336	0.000481467	0.010002647
Ubxn2a	ENSRNOG00000065514	0.842182412	0.046969037	0.210681663
Cyb5rl	ENSRNOG00000009148	0.840230192	0.00684342	0.066223903
Coq2	ENSRNOG00000002194	0.840168513	0.018745541	0.125777573
Coq7	ENSRNOG00000017012	0.839385243	0.006338822	0.063347559
Slc25a4	ENSRNOG00000010830	0.839099567	0.026196221	0.153781883
Uqcr10	ENSRNOG00000059061	0.838774431	0.028720575	0.162360873
Tpi1	ENSRNOG00000015290	0.837725358	0.029702067	0.165553927
Entpd6	ENSRNOG00000007427	0.836918047	0.001816167	0.026820739
Ndufb3	ENSRNOG00000011825	0.835483887	0.024448046	0.147236395
Asb1	ENSRNOG00000020322	0.835421751	0.000236852	0.005669255
Sumf2	ENSRNOG00000000922	0.835062298	0.037433816	0.187355176
Mipep	ENSRNOG00000013876	0.83482536	0.015312447	0.111951163
Cyp4f18	ENSRNOG00000015751	0.833897144	0.015590303	0.113253339
Dnah11	ENSRNOG00000005451	0.833849955	0.000635269	0.01232564
Ttc38	ENSRNOG00000015916	0.833809041	0.000285383	0.006596826
Tab2	ENSRNOG00000016054	0.833718777	0.016605777	0.116487167
Rcor2	ENSRNOG00000060849	0.832926871	0.005487303	0.057300394
Dmrt2	ENSRNOG00000016301	0.832825785	0.002423632	0.032392834
Uqcc2	ENSRNOG00000025909	0.83233638	0.022815039	0.141447792
Bean1	ENSRNOG00000066314	0.831123311	0.048869923	0.215082883
Dusp29	ENSRNOG00000013034	0.830757311	0.047708162	0.212127047
Ddx49	ENSRNOG00000022368	0.830205708	0.000614407	0.012040824
Cited2	ENSRNOG00000056940	0.829435589	0.017057686	0.118463246
Pgm1	ENSRNOG00000009889	0.827448312	0.016668734	0.116771074
Otub2	ENSRNOG00000009117	0.827160065	0.000200656	0.005026068
Mmut	ENSRNOG00000050843	0.827060169	0.017605016	0.120459444
Ccdc124	ENSRNOG00000018932	0.826886089	0.003411476	0.041689072
Lyrm9	ENSRNOG00000037204	0.826753228	0.019757879	0.130318069
Hdhd3	ENSRNOG00000064638	0.826251239	0.006675937	0.06516986
Nhlrc1	ENSRNOG00000026286	0.82558473	0.006282784	0.062949055
Sdhd	ENSRNOG00000022980	0.825227054	0.043370283	0.202489965
Lmod3	ENSRNOG00000032443	0.824526117	0.016536755	0.116369757
Mmd	ENSRNOG00000002436	0.824154035	0.015768844	0.113740086
Myocd	ENSRNOG00000003669	0.823822968	0.014480093	0.10773632
Pold2	ENSRNOG00000014098	0.823090063	0.009198754	0.08044573
Fam78a	ENSRNOG00000023352	0.822093124	0.027608462	0.158664637
Suclg1	ENSRNOG00000005587	0.821698305	0.030693732	0.168222669
Polr2i	ENSRNOG00000020799	0.821246063	0.000648786	0.012525501
Mrps35	ENSRNOG00000001842	0.82098458	0.004562809	0.050663409
Chmp4c	ENSRNOG00000010238	0.820557758	0.034291305	0.179101084
Fam104a	ENSRNOG00000002851	0.819917546	0.007109074	0.067961643
Ccna1	ENSRNOG00000014052	0.818268647	0.003325918	0.040898034
Myh4	ENSRNOG00000049695	0.81800419	0.023290223	0.143546679
Ttc30b	ENSRNOG00000059460	0.817891035	0.034894134	0.180734912
Lias	ENSRNOG00000002759	0.816700342	0.045076651	0.205776494
Mcrip2	ENSRNOG00000020029	0.816004894	0.002523078	0.033426495
Scn4b	ENSRNOG00000026679	0.815760919	0.030069189	0.166544602
Mrpl2	ENSRNOG00000018057	0.815532826	0.022900237	0.141884421

Xrcc2	ENSRNOG00000007493	0.814237534	0.008121734	0.074302178
Chchd2l1	ENSRNOG00000065713	0.813196821	0.025171372	0.149740348
Ddt	ENSRNOG00000068887	0.812560735	0.002905015	0.037098467
Myoz1	ENSRNOG00000040122	0.811214072	0.028414084	0.161153912
Hyi	ENSRNOG00000037518	0.811076797	0.003450869	0.041970762
Rps6ka2	ENSRNOG00000013194	0.809640314	0.020609131	0.133131501
Usp49	ENSRNOG00000013866	0.808877582	0.000168482	0.0043969
Il15ra	ENSRNOG00000018706	0.808721364	0.014827851	0.10953908
Cc2d2b	ENSRNOG00000070964	0.808046865	0.028026121	0.160020323
Acot13	ENSRNOG00000018415	0.807239551	0.012467907	0.097640321
Bcl7a	ENSRNOG00000056017	0.806983802	0.029380232	0.164656122
Ogdh	ENSRNOG00000005130	0.80687542	0.020527516	0.133131501
C1qbp	ENSRNOG00000006949	0.806614658	0.048939855	0.215269041
Vtcn1	ENSRNOG00000015279	0.805592246	0.010113568	0.084988885
Actr3b	ENSRNOG00000031855	0.805508831	0.009049019	0.079608435
Mfsd13a	ENSRNOG00000019674	0.805386119	0.009201174	0.08044573
Serinc2	ENSRNOG00000012989	0.804988976	0.035885457	0.182854236
Prkaa2	ENSRNOG00000007706	0.804170951	0.043431661	0.202533755
Mvd	ENSRNOG00000013376	0.804059657	0.001667868	0.025321547
Carnmt1	ENSRNOG00000012872	0.803857509	0.042253869	0.199792193
St3gal3	ENSRNOG00000019843	0.803452523	0.001826794	0.026903853
Ptges2	ENSRNOG00000014050	0.803198322	0.009037918	0.079608435
Tbc1d10c	ENSRNOG00000021510	0.801433821	0.012130233	0.095884846
Rnf150	ENSRNOG00000003479	0.801283317	0.00453888	0.050475192
Mvk	ENSRNOG00000049604	0.800128106	0.001879217	0.027439002
Uqcrc2	ENSRNOG00000036742	0.799409026	0.03636042	0.184149313
Grik2	ENSRNOG00000000368	0.799025319	0.021539476	0.136135959
Nmnat3	ENSRNOG00000013585	0.79899158	0.000746466	0.013944763
Eepd1	ENSRNOG00000006931	0.797878497	0.007497848	0.070117932
Timm13	ENSRNOG00000019682	0.79722735	0.011229696	0.090934858
Ubl7	ENSRNOG00000007442	0.797079909	0.006425514	0.063865082
Bola3	ENSRNOG00000021866	0.79679211	0.023221445	0.143339982
Rpl8	ENSRNOG00000032635	0.795888329	0.044747851	0.204995198
Tvp23a	ENSRNOG00000025545	0.795812797	0.000602029	0.01190307
Lurap1	ENSRNOG00000062943	0.795617164	0.03425588	0.179068218
Slc25a38	ENSRNOG00000018552	0.794381527	0.000417973	0.008994493
Tm2d3	ENSRNOG00000011290	0.794309376	0.019837038	0.130391307
Mrpl22	ENSRNOG00000027039	0.793847282	0.003350702	0.041170302
Gramd1c	ENSRNOG00000053406	0.793455499	0.036267475	0.183951975
Podxl2	ENSRNOG00000016255	0.793059902	0.010721852	0.088291753
Gsk3a	ENSRNOG00000020417	0.793022743	0.004018303	0.046650646
Hist2h3c2	ENSRNOG00000063016	0.791540407	0.008484685	0.076103281
Rpl3l	ENSRNOG00000014641	0.791248874	0.022117528	0.138724271
Tsfm	ENSRNOG00000048843	0.790394445	0.004517979	0.050337407
Psmb5	ENSRNOG00000013386	0.789869418	0.033282957	0.176317057
Cd1d1	ENSRNOG00000016451	0.788051179	0.043923592	0.203790819
Mecr	ENSRNOG00000028047	0.787842372	0.008453099	0.076022086
Tmem106c	ENSRNOG00000053269	0.787240796	0.004723478	0.0518252
Iftap	ENSRNOG00000037567	0.787173335	0.026422616	0.154702878
Chrna10	ENSRNOG00000020293	0.786852638	0.037389258	0.187247415

Dnajc28	ENSRNOG00000002026	0.786795711	0.011768801	0.093975914
Rai2	ENSRNOG00000006670	0.786676218	0.041800298	0.198672559
Mea1	ENSRNOG00000017144	0.783408358	0.006959656	0.066932988
Abcb9	ENSRNOG00000001082	0.783181715	0.003123736	0.038934239
Mvb12b	ENSRNOG00000017172	0.782690833	0.008006893	0.073765051
Cand2	ENSRNOG00000010473	0.781182719	0.016045062	0.11461989
Dolk	ENSRNOG00000016989	0.781047605	0.011390153	0.091875364
Gde1	ENSRNOG00000050445	0.780674287	0.010023499	0.084688782
Mtfr1	ENSRNOG00000021359	0.779368238	0.010864146	0.089132912
Mtg1	ENSRNOG00000018860	0.77898098	0.023051948	0.142576163
Tufm	ENSRNOG00000018604	0.778257839	0.016281748	0.115539975
Ube2s	ENSRNOG00000016930	0.778211695	0.00755886	0.070603739
Mrps18a	ENSRNOG00000019511	0.77683295	0.001586897	0.024474203
P2rx6	ENSRNOG00000001873	0.77644156	0.016724108	0.117001169
Macrod1	ENSRNOG00000021174	0.776353202	0.007234016	0.068681276
Angel1	ENSRNOG00000010567	0.77622215	0.001212142	0.020090603
Slc25a29	ENSRNOG00000004351	0.776105868	0.004506908	0.050337407
Rad23a	ENSRNOG00000003026	0.775520351	0.037756251	0.188364201
Hp	ENSRNOG00000014964	0.775139821	0.020608001	0.133131501
Slc25a20	ENSRNOG00000020288	0.774112072	0.030597759	0.167856722
Cuedc1	ENSRNOG00000010257	0.774039221	0.002090113	0.029363349
Trim68	ENSRNOG00000047229	0.773469513	0.003912871	0.045905525
Irf2bp1	ENSRNOG00000014252	0.773294275	0.002095321	0.029383527
Ankrd53	ENSRNOG00000021402	0.772902666	0.027249012	0.157236893
Cryab	ENSRNOG00000010524	0.772413569	0.013330885	0.1024138
Sirt3	ENSRNOG00000013828	0.771390885	0.01147329	0.092298091
Prkar2a	ENSRNOG00000020284	0.768878411	0.008922335	0.078938089
Hist1h4m	ENSRNOG00000067648	0.768747089	0.04416721	0.204142982
Mrpl28	ENSRNOG00000042720	0.768068147	0.022960888	0.1421258
Idh3a	ENSRNOG00000010277	0.76692862	0.043422865	0.202533755
Gmpr	ENSRNOG00000017250	0.766637147	0.015945886	0.114328996
Exoc3l4	ENSRNOG00000010111	0.766306855	0.008189563	0.074703392
Fam53a	ENSRNOG00000017548	0.766292589	0.001577286	0.024354918
Tubg1	ENSRNOG00000020213	0.766058872	4.65E-05	0.001601095
Mrpl58	ENSRNOG00000032780	0.765862614	0.019732509	0.130318069
Abcb7	ENSRNOG00000002790	0.765839805	0.032273993	0.173269747
Habp4	ENSRNOG00000019027	0.765756172	0.014011098	0.10556101
Chchd3	ENSRNOG00000013211	0.765315781	0.017389678	0.119405545
Phkb	ENSRNOG00000024101	0.764766526	0.041759788	0.198540586
Napa	ENSRNOG00000001494	0.764575612	0.008134443	0.074336483
Dnaja3	ENSRNOG00000003855	0.763563099	0.007481625	0.070098805
Dnajc4	ENSRNOG00000043060	0.761664743	0.004078778	0.047107019
Gadd45gip	ENSRNOG00000003011	0.761543574	0.040686284	0.196312266
Hoxc9	ENSRNOG00000016199	0.761414236	0.018076608	0.122829032
Lamtor2	ENSRNOG00000019908	0.761109451	0.027146447	0.156993927
Lsm10	ENSRNOG00000068296	0.760009565	0.0005563	0.011197877
Gnpat	ENSRNOG00000019205	0.759288388	0.022450211	0.139986424
Ndufa12	ENSRNOG00000007407	0.758394243	0.039886674	0.19426514
Prpf19	ENSRNOG00000020897	0.75737103	0.019686768	0.130131458
Eif1b	ENSRNOG00000018848	0.754035447	0.005291764	0.05570654

Gps2	ENSRNOG00000016360	0.75395116	0.001343784	0.021628267
Lmo4	ENSRNOG00000009205	0.753639373	0.006211623	0.062466165
Eno3	ENSRNOG00000004078	0.75319242	0.040994541	0.196947067
Dph2	ENSRNOG00000019735	0.75211273	0.023275077	0.143546679
Thap4	ENSRNOG00000018351	0.751313177	0.005729364	0.059036701
Cox6b1	ENSRNOG00000066942	0.751240559	0.003733546	0.044268376
Zfand3	ENSRNOG00000059659	0.750076967	0.029417226	0.164804162
Chac1	ENSRNOG00000014387	0.749916375	0.038316176	0.189935101
Mfn2	ENSRNOG00000046424	0.749318572	0.025214291	0.149881211
Rph3al	ENSRNOG00000061429	0.748459916	0.024255663	0.146360665
Agtpbp1	ENSRNOG00000018651	0.747346884	0.007171947	0.068425554
Rtn2	ENSRNOG00000016603	0.747339807	0.028155612	0.160330566
Ppcs	ENSRNOG00000008572	0.747154245	0.036677068	0.185408408
Rtcb-ps1	ENSRNOG00000005546	0.745778724	0.004100089	0.047248063
Tas1r2	ENSRNOG00000061876	0.745573692	0.000841233	0.015204657
Kcnj11	ENSRNOG00000021128	0.745293085	0.035780366	0.182642627
Fdxr	ENSRNOG00000058497	0.745098074	0.049475805	0.217191939
Sdc3	ENSRNOG00000011927	0.745051841	0.031595469	0.170737524
Hmgcl	ENSRNOG00000009422	0.745031486	0.000191751	0.004889483
Cfap410	ENSRNOG00000001215	0.744578381	0.031054433	0.169170651
Ubqln2	ENSRNOG00000003099	0.744165588	0.015309384	0.111951163
Cdpf1	ENSRNOG00000032846	0.743116811	0.002425673	0.032392834
Atmin	ENSRNOG00000011422	0.742745789	0.033158606	0.175957454
Tars3	ENSRNOG00000024460	0.742555564	0.002174711	0.030144122
Prdx5	ENSRNOG00000021125	0.741764377	0.014367374	0.106999853
Tmub1	ENSRNOG00000013383	0.740808512	0.044534829	0.204618632
Pdzk1	ENSRNOG00000000096	0.73812354	0.024970321	0.1492604
Psmf1	ENSRNOG00000009640	0.738041971	0.006837752	0.066210174
Ndufv3	ENSRNOG00000001182	0.737947363	0.007763895	0.072172727
Fam216a	ENSRNOG00000031731	0.737750711	0.004022288	0.046662132
Fyco1	ENSRNOG00000006336	0.736719436	0.006628944	0.064905582
Mul1	ENSRNOG00000016010	0.735956957	0.013118921	0.101184549
Alg3	ENSRNOG00000001712	0.735703865	0.010947566	0.089487448
Dda1	ENSRNOG00000039417	0.734288438	0.001509496	0.023470411
Endog	ENSRNOG00000016033	0.733787984	0.029486313	0.165072496
Thop1	ENSRNOG00000019924	0.732684503	0.005479607	0.057282684
Exosc5	ENSRNOG00000020635	0.732473201	0.004333242	0.04892167
Mtg2	ENSRNOG00000059919	0.732259722	0.018901965	0.126553749
Plpbp	ENSRNOG00000013751	0.731838317	0.020946025	0.134427183
Wfikkn1	ENSRNOG00000021578	0.731756923	0.03471039	0.180322735
Snrnp35	ENSRNOG00000001060	0.731106184	0.000250033	0.005938272
Tmem234	ENSRNOG00000049326	0.73102049	0.009791332	0.083542691
Znrd2	ENSRNOG00000012736	0.73080214	0.02366277	0.144518208
Ccdc91	ENSRNOG00000001847	0.730562386	0.012660054	0.098769978
Mrps15	ENSRNOG00000008279	0.730190859	0.04121894	0.197343617
Coq8a	ENSRNOG00000043201	0.730160829	0.026059729	0.153211541
Sema6c	ENSRNOG00000021101	0.729188084	0.031588261	0.170737524
Fiz1	ENSRNOG00000016574	0.728458943	0.020850759	0.134126682
Lmtk2	ENSRNOG00000025155	0.728220036	0.009950046	0.084296744
Twnk	ENSRNOG00000014935	0.728153336	0.029640182	0.165553927

Utp23	ENSRNOG00000063314	0.728123593	0.028855613	0.162791512
Chchd7	ENSRNOG00000031769	0.727453462	0.034418356	0.179525247
Plaat3	ENSRNOG00000021206	0.726430566	0.027781881	0.159326477
Cops7a	ENSRNOG00000016778	0.726345645	0.021784536	0.137077168
Gpx4	ENSRNOG00000013604	0.725818782	0.049511001	0.217229341
Pex10	ENSRNOG00000024012	0.724963836	0.000865587	0.015554614
Nr1d2	ENSRNOG00000046912	0.724477241	0.031809935	0.171428153
Fzd7	ENSRNOG00000016119	0.72439999	0.041832192	0.198685187
Sphk2	ENSRNOG00000021032	0.723553931	0.011880576	0.094438453
Plcl2	ENSRNOG00000013368	0.723388934	0.019434008	0.12928345
Zfp954	ENSRNOG00000022742	0.723076503	0.046021055	0.208390148
Dmac2	ENSRNOG00000020596	0.722868651	0.017418929	0.119553709
Mrpl27	ENSRNOG00000003724	0.722321122	0.042593457	0.200517654
Slc2a8	ENSRNOG00000022274	0.721996318	0.000963069	0.016859121
Frat2	ENSRNOG00000045543	0.721521379	0.022946825	0.142095202
Mtm1	ENSRNOG00000002516	0.721030106	0.00103834	0.017816458
Acot8	ENSRNOG00000015187	0.720578441	0.020978698	0.134450066
Atp9a	ENSRNOG00000049484	0.72030001	0.011839824	0.094271116
Sdr39u1	ENSRNOG00000020544	0.719295276	0.020388427	0.13257583
Amacr	ENSRNOG00000018662	0.716337179	0.010021808	0.084688782
Timm50	ENSRNOG00000037638	0.715967633	0.01246726	0.097640321
Traf4	ENSRNOG00000013169	0.714589548	0.017048741	0.118463246
Ethe1	ENSRNOG00000019982	0.713775484	0.008298307	0.075224296
Kif5a	ENSRNOG00000005299	0.713340578	0.041315867	0.197380187
Snx3	ENSRNOG00000046705	0.712909089	0.029521468	0.165163151
Polr3d	ENSRNOG00000010028	0.712638514	0.021455321	0.136025567
Csnk2b	ENSRNOG00000000847	0.711169655	0.020021569	0.131009948
Ece1	ENSRNOG00000014241	0.711054001	0.000247581	0.005891059
Gaa	ENSRNOG00000047656	0.710847292	0.003098443	0.038805264
Gdf1	ENSRNOG00000070518	0.710655712	0.004769739	0.052149152
Tmem42	ENSRNOG00000032027	0.710454629	0.023834668	0.145112984
Gcdh	ENSRNOG00000003307	0.70915697	0.024239769	0.146321423
Riox2	ENSRNOG00000001680	0.709023303	0.021285829	0.135526445
Dtd1	ENSRNOG00000008746	0.708404335	0.004760982	0.052089956
Glud1	ENSRNOG00000057367	0.707964085	0.018570345	0.125195144
Sod1	ENSRNOG00000002115	0.707867156	0.009523873	0.082251633
Dhps	ENSRNOG00000004219	0.707142643	0.010082953	0.084947786
Ghr	ENSRNOG00000015654	0.705429624	0.033932433	0.177882673
Camk2g	ENSRNOG00000009783	0.70503903	0.006174599	0.062374932
Phc1	ENSRNOG00000015191	0.704137056	0.000719993	0.013603908
Eef1a2	ENSRNOG00000011624	0.703519047	0.029358935	0.16459597
Wnt11	ENSRNOG00000015982	0.703129024	0.015008309	0.110609962
Znf768	ENSRNOG00000029490	0.702576636	0.009602846	0.082658748
Poldip2	ENSRNOG00000009252	0.70143291	0.010999463	0.089723367
Slc25a28	ENSRNOG00000016751	0.701405582	0.011090915	0.090233134
Ppm1a	ENSRNOG00000005916	0.701043383	0.039446008	0.193139159
Scn1b	ENSRNOG00000021102	0.700545918	0.04597252	0.208333876
Agtrap	ENSRNOG00000008619	0.700468057	0.007329241	0.069205807
Pdk1	ENSRNOG00000001517	0.700119077	0.049606357	0.217505158
Zfp574	ENSRNOG00000055761	0.69990239	0.000219723	0.005342284

Tango2	ENSRNOG00000030245	0.69970555	0.00521721	0.055373135
Gstt2	ENSRNOG00000052415	0.698593022	0.003535172	0.042696114
Cgrrf1	ENSRNOG00000010178	0.697949252	0.022681677	0.140960961
Isoc2a	ENSRNOG00000050348	0.697538216	0.004449613	0.04983823
Mok	ENSRNOG00000007850	0.697473141	0.044124597	0.204084167
Cmya5	ENSRNOG00000023803	0.697283309	0.028783116	0.162655402
Rfng	ENSRNOG00000050298	0.696549742	0.010860004	0.089132912
Emc9	ENSRNOG00000019162	0.69650109	0.033092996	0.175848869
Micos13	ENSRNOG00000046002	0.696004601	0.035024331	0.180988087
Tmem238	ENSRNOG00000070914	0.694724293	0.029146945	0.163820129
Entrep3	ENSRNOG00000020518	0.694204141	0.018604289	0.125315528
Clptm1	ENSRNOG00000018255	0.693544005	0.018399477	0.124582289
Cbx7	ENSRNOG00000016875	0.693197354	0.004327946	0.048897314
Casp7	ENSRNOG00000056216	0.692371339	0.01563876	0.113403432
Zfp667	ENSRNOG00000033906	0.692241528	0.027571385	0.158510027
Ccdc6	ENSRNOG00000024019	0.691583255	0.029653318	0.165553927
Tmem201	ENSRNOG00000023152	0.691334362	8.25E-05	0.002505064
Zfp879	ENSRNOG00000030517	0.691125207	0.011626434	0.093226888
Uaca	ENSRNOG00000012868	0.690014269	0.002550011	0.033652153
Dis3l	ENSRNOG00000010537	0.689999096	0.005476947	0.057282684
Tomm34	ENSRNOG00000029799	0.689350429	0.00655412	0.064546898
Fech	ENSRNOG00000018053	0.68889649	0.042107006	0.19927921
Ntmt1	ENSRNOG00000024809	0.687776077	0.002062616	0.029055663
Unc45b	ENSRNOG00000009466	0.686628394	0.020542711	0.133131501
Mier2	ENSRNOG00000000175	0.68600497	0.030160631	0.16663214
Susd4	ENSRNOG00000003562	0.685098445	0.033331916	0.176456422
Pygo1	ENSRNOG00000066086	0.684715905	0.013963752	0.105302643
Hsp90ab1	ENSRNOG00000019834	0.684166181	0.019843247	0.130391307
Sephs2	ENSRNOG00000048397	0.683991916	0.042030745	0.19909909
Zfp362	ENSRNOG00000043364	0.683834562	0.039920526	0.194302343
Ndufa13	ENSRNOG00000020602	0.682522729	0.043276245	0.202183377
Cwc15	ENSRNOG00000008490	0.68227216	0.015624453	0.113403432
Oma1	ENSRNOG00000007214	0.682049809	0.030960476	0.169031608
Pdzrn3	ENSRNOG00000057556	0.680469154	0.038916053	0.191628354
Banf1	ENSRNOG00000020460	0.680193346	0.039758593	0.194059797
Slc25a42	ENSRNOG00000020345	0.67913974	0.026776219	0.155603692
Smdt1	ENSRNOG00000070907	0.678721628	0.047775595	0.212201518
Syngr2	ENSRNOG00000050547	0.678721409	0.041254696	0.197343617
Polr2c	ENSRNOG00000015720	0.678646894	0.021740481	0.137076769
Dpcd	ENSRNOG00000017241	0.677807634	0.025018842	0.149288993
Rab3a	ENSRNOG00000019433	0.677766066	0.007401027	0.069664701
Ube2g1	ENSRNOG00000010041	0.677410757	0.020606182	0.133131501
Metap1d	ENSRNOG00000061587	0.677137397	0.009039923	0.079608435
Map10	ENSRNOG00000028652	0.676635495	0.030585763	0.167856722
Fam222b	ENSRNOG00000029570	0.676586766	0.007081083	0.067861739
Dynlrb1	ENSRNOG00000025715	0.676342409	0.036353397	0.184149313
Hoxc10	ENSRNOG00000016149	0.675901949	0.045665436	0.207424737
Nudt8	ENSRNOG00000017955	0.675233335	0.033493546	0.176755387
Mocs2	ENSRNOG00000056325	0.675181177	0.035014093	0.180988087
Sdhb	ENSRNOG00000007967	0.675012436	0.025047573	0.149346034

Mmachc	ENSRNOG00000017233	0.674891942	0.031227088	0.16975507
Thap3	ENSRNOG00000026840	0.674526778	0.031164046	0.169530672
Zfp777	ENSRNOG00000033639	0.674449637	0.009636007	0.082806943
Snf8	ENSRNOG00000006500	0.674312438	0.03846066	0.190407711
Acad9	ENSRNOG00000014178	0.673212228	0.021504917	0.136031913
Myh14	ENSRNOG00000020014	0.672540962	0.00426357	0.048479639
Rfx8	ENSRNOG00000058243	0.67046559	0.010610314	0.087912392
Zyg11b	ENSRNOG00000010859	0.66925697	0.040434221	0.195856293
Vsig10l	ENSRNOG00000023411	0.667129093	0.045678708	0.207424737
Fam234b	ENSRNOG00000008443	0.66550903	0.006751985	0.065583492
Dctn3	ENSRNOG00000014208	0.665438655	0.007550462	0.070567607
Mrps11	ENSRNOG00000018531	0.665195803	0.020605562	0.133131501
Ntpcr	ENSRNOG00000019922	0.665003636	0.008095946	0.074153344
Qdpr	ENSRNOG00000003253	0.664747294	0.035789983	0.182642627
Mavs	ENSRNOG00000025295	0.664572895	0.013528407	0.103067274
Echdc1	ENSRNOG00000011622	0.664486651	0.020804585	0.133940264
Masp2	ENSRNOG00000011258	0.663801328	0.040628365	0.196154298
Aldh5a1	ENSRNOG00000023538	0.662449845	0.013190864	0.101538368
Phb1	ENSRNOG00000046799	0.66025811	0.048431781	0.21381897
Get3	ENSRNOG00000003747	0.660084649	0.004324006	0.048897314
Mybpc2	ENSRNOG00000019627	0.659508648	0.047767201	0.212201518
Ret	ENSRNOG00000014751	0.65840931	0.040408241	0.195856293
Psmc5	ENSRNOG00000010038	0.658314944	0.020916285	0.134378094
Tma7	ENSRNOG00000047225	0.657891836	0.045351748	0.206240584
Rmnd5a	ENSRNOG00000028422	0.657630224	0.039051853	0.191933083
Ndrg2	ENSRNOG00000010389	0.657355242	0.020361822	0.132458116
Zfp91	ENSRNOG00000012524	0.657227202	0.039020304	0.191838541
Slc22a5	ENSRNOG00000008432	0.657187771	0.002015469	0.028781861
Bola1	ENSRNOG00000021185	0.655981859	0.029839948	0.165801137
Ubiad1	ENSRNOG00000009575	0.655845192	0.045094024	0.205789364
Zfp467	ENSRNOG00000007707	0.655646959	0.008269882	0.075171738
Zfp341	ENSRNOG00000017086	0.655562641	0.008619558	0.076964648
Ogfod1	ENSRNOG00000019288	0.653709333	0.019940468	0.130759427
Vps18	ENSRNOG00000013689	0.653494628	0.004799567	0.052401722
Ncdn	ENSRNOG00000011751	0.653414022	0.008380616	0.075619381
Echdc3	ENSRNOG00000048114	0.652969846	0.014648829	0.108422211
Pick1	ENSRNOG00000011507	0.651831691	0.00498465	0.053819025
Dele1	ENSRNOG00000019276	0.651762349	0.020298006	0.132142294
Zmynd19	ENSRNOG00000007752	0.651629341	0.001491136	0.023278458
Polr2f	ENSRNOG00000011214	0.651423734	0.001527238	0.023675995
Isoc2b	ENSRNOG00000016829	0.650941635	0.025580932	0.151252721
Cbr2	ENSRNOG00000069492	0.650154547	0.043415386	0.202533755
Rbm38	ENSRNOG00000006420	0.649663847	0.04329164	0.202183377
Adcy9	ENSRNOG00000049768	0.649199267	0.024052191	0.145640552
Hoxa3	ENSRNOG00000006281	0.64908745	0.011774133	0.093975914
Pias4	ENSRNOG00000020230	0.649055656	0.000666581	0.012789819
Ssc4d	ENSRNOG00000001435	0.648836265	0.033353654	0.176491006
Cyp2u1	ENSRNOG00000011053	0.648493558	0.017532466	0.120068491
Fancf	ENSRNOG00000023968	0.647976668	0.01761463	0.120472314
Stoml2	ENSRNOG00000009535	0.646001252	0.026543418	0.154996014

Alkbh5	ENSRNOG00000028341	0.645837528	0.009035609	0.079608435
Eci1	ENSRNOG00000008843	0.642651919	0.012696762	0.098808964
Sart1	ENSRNOG00000020475	0.642600262	1.27E-06	8.12E-05
Rpusd4	ENSRNOG00000011567	0.641795085	0.002692385	0.035220285
Nsmce1	ENSRNOG00000015218	0.641566408	0.006495394	0.064248909
Ptpn11	ENSRNOG00000030124	0.641424924	0.025751941	0.152028743
Slc35f6	ENSRNOG00000009459	0.64107614	0.006811	0.066061576
Alad	ENSRNOG00000015206	0.640401442	0.007179126	0.068452129
Rpp25l	ENSRNOG00000064027	0.639764873	0.015908761	0.114220506
Nfatc2	ENSRNOG00000012175	0.63897236	0.005263942	0.055597963
Rab4a	ENSRNOG00000017467	0.63775361	0.023198905	0.143314411
Acot2	ENSRNOG00000010134	0.637163416	0.01206282	0.095500362
Fhip2b	ENSRNOG00000012014	0.636745749	0.005712056	0.058916724
Efna1	ENSRNOG00000020573	0.636544467	0.008461302	0.076024852
Borcs6	ENSRNOG00000006513	0.636448276	0.01424172	0.106602701
Pnrc1	ENSRNOG00000007793	0.635959114	0.033029576	0.1756889
Snx15	ENSRNOG00000021007	0.63442559	0.000159128	0.004223537
Apba3	ENSRNOG00000020466	0.63438661	0.002760737	0.035783923
Mfsd6	ENSRNOG00000012663	0.634315865	0.011475058	0.092298091
Atp6v1fnb	ENSRNOG00000063021	0.632284237	0.041938836	0.198881217
Hadha	ENSRNOG00000024629	0.631948826	0.026493679	0.154893461
Gemin7	ENSRNOG00000049772	0.631189561	0.044599345	0.204618632
Fbxo6	ENSRNOG00000009217	0.630523949	0.017360324	0.119405545
Dnajc21	ENSRNOG00000017876	0.630127146	0.010992445	0.089723367
Aars2	ENSRNOG00000025808	0.627760179	0.007920518	0.07336603
Fbxo40	ENSRNOG00000002459	0.62747827	0.027321826	0.157482075
Zfp3	ENSRNOG00000005009	0.627038314	0.010192875	0.085470934
Bud23	ENSRNOG00000033700	0.626876233	0.001135334	0.019019889
Scrn3	ENSRNOG00000018657	0.626771948	0.026817665	0.155641845
Eif6	ENSRNOG00000049497	0.625471703	0.006127117	0.062063105
Rangrf	ENSRNOG00000004980	0.625459714	0.04245594	0.200382775
Mfap1a	ENSRNOG00000065756	0.623469449	0.017062357	0.118463246
Mex3d	ENSRNOG00000030830	0.622934437	0.034849706	0.180684999
Rxra	ENSRNOG00000009446	0.6229302	0.00225432	0.030782041
Mettl21a	ENSRNOG00000014212	0.622766436	0.006886643	0.066518227
Fbxw5	ENSRNOG00000028674	0.622555046	0.019966411	0.130759427
Slc25a35	ENSRNOG00000004668	0.622472281	0.01088276	0.089182821
Pecr	ENSRNOG00000055295	0.621777709	0.036070949	0.183236187
Vamp5	ENSRNOG00000012659	0.621774997	0.046242671	0.208778427
Nt5m	ENSRNOG00000003332	0.620936763	0.01325066	0.10194829
Mrpl30	ENSRNOG00000049330	0.620865749	0.035543969	0.182102939
Cox18	ENSRNOG00000003052	0.620723376	0.036899198	0.185653992
Sptb	ENSRNOG00000006911	0.620477217	0.022141306	0.138761684
Anapc13	ENSRNOG00000008459	0.620415701	0.019704107	0.130190835
Pex14	ENSRNOG00000013498	0.619920785	0.006367099	0.063507944
Lyar	ENSRNOG00000005374	0.619873769	0.00806148	0.074011702
Ube3b	ENSRNOG00000047219	0.619868159	0.041181672	0.197343617
Cdc37	ENSRNOG00000033426	0.619702977	0.015715768	0.113567565
Brms1	ENSRNOG00000020117	0.619072052	0.003579589	0.043065635
Fbxo21	ENSRNOG00000001129	0.618787304	0.010944498	0.089487448

Ubqln4	ENSRNOG00000019933	0.618187302	0.026615566	0.155133005
Mrps10	ENSRNOG00000022609	0.616655383	0.036865132	0.185653992
Mrps7	ENSRNOG00000003797	0.615523976	0.026742112	0.155541632
Tmem63b	ENSRNOG00000019743	0.615328492	4.24E-05	0.001494069
Tmem52	ENSRNOG00000016618	0.615164872	0.022986548	0.142228125
Klhl26	ENSRNOG00000020088	0.614288562	0.037577167	0.187865853
Paqr7	ENSRNOG00000022054	0.613938868	0.026500436	0.154893461
Ogfr	ENSRNOG00000009355	0.613878591	0.015492958	0.112794526
Rab5if	ENSRNOG00000020317	0.613746813	0.023885618	0.145252898
Acvr2b	ENSRNOG00000014477	0.612408513	0.039116988	0.19213199
Nudt6	ENSRNOG00000017420	0.612246833	0.028681632	0.162207238
Bud13	ENSRNOG00000018665	0.611487554	0.044444267	0.204561798
Cep131	ENSRNOG00000004430	0.611351907	0.000459731	0.0096792
Srm	ENSRNOG00000011078	0.611072695	0.000393295	0.008581974
Mmab	ENSRNOG00000049426	0.610737544	0.007154843	0.068338326
Pdcd2l	ENSRNOG00000021119	0.609991773	0.018515985	0.125099323
Taf5l	ENSRNOG00000018027	0.608714997	0.031363944	0.170202105
Alkbh6	ENSRNOG00000025216	0.608670423	0.016187317	0.115211698
Wdr18	ENSRNOG00000012379	0.606729235	0.035710247	0.182594569
Kif21a	ENSRNOG00000014844	0.606510561	0.007356799	0.069381921
Nampt	ENSRNOG00000009754	0.605658405	0.041303203	0.197380187
Tomm7	ENSRNOG00000043114	0.604667654	0.043703875	0.203255634
Atg9a	ENSRNOG00000018975	0.601881538	0.026493787	0.154893461
Mcm2	ENSRNOG00000016316	0.601622715	0.024701483	0.148423618
Klhdc3	ENSRNOG00000017495	0.600779593	0.038633507	0.190666315
Psma7	ENSRNOG00000056853	0.600614699	0.03277984	0.174960573
Gfus	ENSRNOG00000009020	0.600559823	0.002438878	0.032532292
Zfp623	ENSRNOG00000009302	0.599589423	0.026633352	0.155178616
Ttc39a	ENSRNOG00000047573	0.598441501	0.011192837	0.090777931
Acyp1	ENSRNOG00000006744	0.598043826	0.049693229	0.217706658
Polr1b	ENSRNOG00000018349	0.597211258	0.018669032	0.125414757
C5h1orf174	ENSRNOG00000025034	0.596787707	0.04596963	0.208333876
Mturn	ENSRNOG00000010205	0.596341988	0.033592028	0.176838333
Cdkn1b	ENSRNOG00000007249	0.596113039	0.004165855	0.047793829
Nudc	ENSRNOG00000051720	0.596044973	0.015878681	0.114109713
Epas1	ENSRNOG00000021318	0.595461209	0.03153162	0.170636555
Kdm8	ENSRNOG00000015180	0.595308189	0.025513982	0.150914138
Tceanc2	ENSRNOG00000009398	0.595111335	0.020954011	0.134427183
Klhl25	ENSRNOG00000010959	0.592501485	0.038635017	0.190666315
Ahsa1	ENSRNOG00000048981	0.592218931	0.019247572	0.128317149
Thnsl2	ENSRNOG00000006508	0.591923042	0.021605603	0.136391938
Axin2	ENSRNOG00000055010	0.591314487	0.02721824	0.15717575
Jrk	ENSRNOG00000027238	0.591231022	0.015642104	0.113403432
Bysl	ENSRNOG00000049121	0.590887439	0.000918546	0.01622553
Mbd3	ENSRNOG00000028956	0.590605062	0.025770726	0.152028743
Tomm40	ENSRNOG00000018556	0.589752561	0.016252458	0.115464338
Mthfd1	ENSRNOG00000005602	0.589590371	0.006598717	0.064875554
Gpr180	ENSRNOG00000009766	0.589171302	0.00457099	0.050687562
Adrm1	ENSRNOG00000055984	0.5877776	0.005908085	0.060319763
Ahcy	ENSRNOG00000017777	0.5868452	0.042884162	0.201184957

Romo1	ENSRNOG00000045555	0.586618528	0.040325657	0.195541157
Ythdf1	ENSRNOG00000027015	0.586180213	0.023518151	0.144086823
Gtpbp3	ENSRNOG00000023403	0.586120491	0.019865256	0.130425912
Abcf2	ENSRNOG00000010609	0.584144276	0.015230645	0.111720082
Polr2e	ENSRNOG00000013545	0.584108184	0.031572512	0.170737524
Zfp518b	ENSRNOG00000028534	0.583845075	0.027451688	0.158055175
Ppp5c	ENSRNOG00000016907	0.58339853	0.004901863	0.053146152
Rbm42	ENSRNOG00000024278	0.582822168	0.01234658	0.096955502
Mepce	ENSRNOG00000001387	0.582246799	0.03041994	0.167411751
Acad8	ENSRNOG00000048164	0.580679211	0.046159482	0.20871032
Atat1	ENSRNOG00000000809	0.579972476	0.022453535	0.139986424
Tmed4	ENSRNOG00000005016	0.579314333	0.030158886	0.16663214
Tysnd1	ENSRNOG00000024839	0.579304732	0.008856307	0.078577029
Dagla	ENSRNOG00000027264	0.578528615	0.002380117	0.032071702
Prune1	ENSRNOG00000021120	0.577864321	0.036282697	0.183951975
Nrdc	ENSRNOG00000007111	0.57718536	0.027126689	0.156937917
Pcbp1	ENSRNOG00000017708	0.576649129	0.022736722	0.141130726
Spata2	ENSRNOG00000009207	0.576526249	0.007970024	0.073665937
Zfp787	ENSRNOG00000015456	0.576242006	0.036884604	0.185653992
Paxx	ENSRNOG00000015294	0.576131407	0.02309003	0.142755028
Zfp524	ENSRNOG00000016565	0.575899853	0.042812447	0.2010487
Usp10	ENSRNOG00000016509	0.575776176	0.001301166	0.021160923
Pdap1	ENSRNOG00000000990	0.575044075	0.028419613	0.161153912
Suclg2	ENSRNOG00000005686	0.574995865	0.013600394	0.103417027
Bpnt1	ENSRNOG00000002378	0.574779627	0.028216516	0.160568808
Sars2	ENSRNOG00000019962	0.574343486	0.010471793	0.087060053
Skic2	ENSRNOG00000000421	0.574263325	0.001992524	0.028506445
Epn1	ENSRNOG00000015753	0.573815914	0.046530034	0.209459097
Naxe	ENSRNOG00000019201	0.573559855	0.039603834	0.193790117
Myg1	ENSRNOG00000013343	0.573551426	0.008837706	0.078456673
Acadvl	ENSRNOG00000018114	0.572393498	0.02974799	0.165565884
Ntng1	ENSRNOG00000031136	0.572267331	0.046499917	0.209459097
Jsrp1	ENSRNOG00000032951	0.57110844	0.048187103	0.2131882
Zfp133	ENSRNOG00000027955	0.570648386	0.009899711	0.084053131
Rpl7a	ENSRNOG00000070974	0.569835327	0.038945747	0.191713916
Vkorc1l1	ENSRNOG00000000901	0.569610162	0.007404725	0.069664701
Yif1b	ENSRNOG00000055286	0.569491897	0.043616452	0.203030868
Nprl3	ENSRNOG00000020541	0.56909063	0.001730998	0.025881915
Pih1d1	ENSRNOG00000020634	0.569077213	0.033992292	0.178076635
Mccc1	ENSRNOG00000013293	0.567188968	0.003096067	0.038805264
Ppif	ENSRNOG00000010558	0.566602845	0.013762821	0.104292194
Agbl5	ENSRNOG00000008612	0.563944093	0.044881003	0.205238046
Trap1	ENSRNOG00000005418	0.561813296	0.009879622	0.084009932
Cdk2ap2	ENSRNOG00000018391	0.561522318	0.023738654	0.144698054
Plec	ENSRNOG00000023781	0.561511693	0.02695143	0.156214019
Ybx2	ENSRNOG00000017117	0.559279941	0.039823357	0.194193396
Dhx32	ENSRNOG00000018119	0.558852528	0.025397104	0.150451285
Arl6ip4	ENSRNOG00000001080	0.558797104	0.02379103	0.14490393
Eif2b1	ENSRNOG00000001039	0.558771458	0.005191477	0.055172726
Scn4a	ENSRNOG00000012134	0.558451721	0.043204254	0.202017492

Park7	ENSRNOG00000018289	0.558358883	0.049006127	0.215438898
Stk11	ENSRNOG00000014287	0.557466921	0.022408519	0.139830986
Bsdc1	ENSRNOG00000008338	0.55616925	0.003052244	0.038505232
Art5	ENSRNOG00000020242	0.555862765	0.042416733	0.200258395
Ccnk	ENSRNOG00000006985	0.555790858	0.019790279	0.130318069
Eftud2	ENSRNOG00000002818	0.553770952	0.003992537	0.04652485
Flywch1	ENSRNOG00000004333	0.553562506	0.003884647	0.045608736
Bcl7b	ENSRNOG00000032705	0.552415142	0.002813615	0.036258171
Kat8	ENSRNOG00000019485	0.551993427	0.045459617	0.206670799
Ints15	ENSRNOG00000024567	0.550525558	0.003104021	0.03884389
Pold4	ENSRNOG00000018765	0.550373303	0.014763516	0.109115553
Dnmt3a	ENSRNOG00000026649	0.5484183	0.015474554	0.112765929
Bag6	ENSRNOG00000000851	0.548337834	0.030624377	0.167943611
Trpt1	ENSRNOG00000023023	0.547097332	0.027938397	0.159846315
Lonp1	ENSRNOG00000046502	0.546793306	0.000796483	0.014564788
Mto1	ENSRNOG00000037659	0.546411208	0.024016953	0.145567199
Fxyd1	ENSRNOG00000021079	0.545917234	0.020642012	0.133223922
Imp4	ENSRNOG00000013285	0.543920382	0.023110731	0.142826331
Zfp496	ENSRNOG00000003129	0.54366128	0.024962024	0.1492604
Ears2	ENSRNOG00000025353	0.543034444	0.045142018	0.205887777
Ppm1g	ENSRNOG00000026905	0.542120255	0.037925544	0.188695521
Tepsin	ENSRNOG00000028161	0.541708052	0.012074446	0.095540817
Eml2	ENSRNOG00000030127	0.541490935	0.020957871	0.134427183
Cpox	ENSRNOG00000001654	0.541301895	0.019960691	0.130759427
Ccdc86	ENSRNOG00000020925	0.540860591	0.022748602	0.141148236
Abcb10	ENSRNOG00000017993	0.540127947	0.025165281	0.149740348
Slc25a39	ENSRNOG00000020994	0.538942712	0.014577393	0.108150374
Ulk1	ENSRNOG00000037505	0.538873759	0.016145164	0.115122042
Shmt2	ENSRNOG00000008106	0.538318114	0.005182402	0.055151522
Sars1	ENSRNOG00000020255	0.538199463	0.030395901	0.167397718
Polg	ENSRNOG00000032293	0.537699435	0.010237748	0.08557088
Rrp36	ENSRNOG00000017836	0.537543437	0.01134643	0.091641978
Stard3	ENSRNOG00000042044	0.537079217	0.021473062	0.136025567
Fbxo31	ENSRNOG00000042274	0.536541058	0.002425176	0.032392834
Lyrm4	ENSRNOG00000047970	0.536222763	0.044406216	0.204490707
Keap1	ENSRNOG00000020878	0.536212662	0.012745824	0.099091786
Prkce	ENSRNOG00000015603	0.535987824	0.009006761	0.079459422
Fxr2	ENSRNOG00000011876	0.535050623	0.008727948	0.077654165
Echdc2	ENSRNOG00000029333	0.535049489	0.048367337	0.213716139
Cbfa2t3	ENSRNOG00000014723	0.535016054	0.011511653	0.092496932
Hdhd5	ENSRNOG00000011338	0.533713222	0.016067618	0.114726624
Dcaf4	ENSRNOG00000008399	0.530317932	0.013823006	0.104443471
Tom1	ENSRNOG00000014093	0.52971603	0.014629006	0.108378466
Mapkapk5	ENSRNOG00000001345	0.528822855	0.034262025	0.179068218
Psmd7	ENSRNOG00000014097	0.527937928	0.024960929	0.1492604
Ficd	ENSRNOG00000059799	0.527570722	0.036146437	0.183499997
Dcaf5	ENSRNOG00000004556	0.527415861	0.018602951	0.125315528
Fut10	ENSRNOG00000050857	0.525417732	0.036843155	0.185653992
Vps33a	ENSRNOG00000056889	0.525230334	0.019584546	0.129819618
Exoc3	ENSRNOG00000039776	0.524612529	0.042489409	0.200480008

Itpk1	ENSRNOG00000008051	0.523357086	0.00845012	0.076022086
Babam1	ENSRNOG00000017071	0.520191439	0.046120202	0.208639007
Gart	ENSRNOG00000028292	0.519604587	0.016481827	0.116245754
Imp3	ENSRNOG00000017460	0.519561191	0.049373107	0.21692978
Rnf130	ENSRNOG00000046330	0.518439707	0.046523078	0.209459097
Acy1	ENSRNOG00000011189	0.518220101	0.014914302	0.110125507
Ptcd1	ENSRNOG00000000987	0.514872819	0.031189012	0.169607261
Adrb2	ENSRNOG00000019217	0.514509276	0.048322916	0.213580435
Tp53i11	ENSRNOG00000008738	0.514448451	0.038587154	0.190666315
Gjc1	ENSRNOG00000048838	0.514198708	0.014279156	0.106786393
Psmc3ip	ENSRNOG00000020022	0.51410892	0.044601083	0.204618632
Grwd1	ENSRNOG00000021058	0.513602285	0.008204682	0.074710083
Hmbs	ENSRNOG00000010390	0.512701745	0.042816405	0.2010487
Abhd11	ENSRNOG00000018910	0.512113442	0.014347022	0.106954028
R3hdm2	ENSRNOG00000007801	0.5112403	0.027007416	0.156480308
Pcca	ENSRNOG00000057042	0.510023507	0.028944712	0.16308191
Cox10	ENSRNOG00000024972	0.509678558	0.022706731	0.141000744
Zfp523	ENSRNOG00000000501	0.50933095	0.019617258	0.129892428
Rrp15	ENSRNOG00000002450	0.509245709	0.038780539	0.191192463
Polrmt	ENSRNOG00000024879	0.508009213	0.014011564	0.10556101
Aldh2	ENSRNOG00000037815	0.507711373	0.035859503	0.182854236
Chaf1a	ENSRNOG00000046479	0.507027508	0.044209141	0.204142982
Zcchc14	ENSRNOG00000018226	0.506931907	0.000898554	0.015999403
Acot7	ENSRNOG00000010580	0.506740937	0.013344271	0.102466114
Map7d1	ENSRNOG00000010237	0.50571311	0.042547386	0.200517654
Bbs1	ENSRNOG00000019832	0.505131628	0.003951524	0.04615186
Pram1	ENSRNOG00000008378	0.505124676	0.028447923	0.161229043
Dnaaf5	ENSRNOG00000021312	0.502363623	0.013789108	0.104339147
Ptdss2	ENSRNOG00000015912	0.501982856	0.03465829	0.180172227
Ighmbp2	ENSRNOG00000013456	0.501812812	0.013963065	0.105302643
Doc2g	ENSRNOG00000018029	0.501145683	0.049690971	0.217706658
E2f3	ENSRNOG00000029273	0.501108134	0.009537139	0.082320572
Tut1	ENSRNOG00000020047	0.501036166	0.009374659	0.081414265
C4h2orf81	ENSRNOG00000010048	0.500884763	0.00507172	0.054382245
Lig3	ENSRNOG00000021815	0.500272005	0.011342134	0.091641978
Trappc6a	ENSRNOG00000017468	0.499953562	0.045757811	0.207723396
Atxn1	ENSRNOG00000016998	0.499816901	0.019566352	0.129819618
Acsf3	ENSRNOG00000015077	0.496368027	0.038449213	0.190407711
Trpc4ap	ENSRNOG00000019152	0.495519678	0.044571335	0.204618632
Smox	ENSRNOG00000021255	0.494448801	0.018909957	0.126553749
Rnf126	ENSRNOG00000009028	0.493631472	0.014243202	0.106602701
Tfip11	ENSRNOG00000000663	0.492826349	0.003416735	0.041689072
Vps51	ENSRNOG00000020996	0.490898116	0.036862639	0.185653992
Ccdc51	ENSRNOG00000020688	0.490677727	0.028952796	0.16308191
Patz1	ENSRNOG00000018709	0.490434335	0.014210395	0.106568425
Eefsec	ENSRNOG00000012954	0.489875694	0.022206693	0.138947902
Klc2	ENSRNOG00000020299	0.487445964	0.017384865	0.119405545
Ryr1	ENSRNOG00000020557	0.487441201	0.049910222	0.218192621
Sirt2	ENSRNOG00000020102	0.486048896	0.03535427	0.181690679
Thap7	ENSRNOG00000037967	0.482570996	0.035099792	0.181018762

Zkscan7	ENSRNOG00000004104	0.482042627	0.033080375	0.175841775
Mettl13	ENSRNOG00000003042	0.479638587	0.007036356	0.067587193
Atg2a	ENSRNOG00000060707	0.478503245	0.004821955	0.052535705
Arih2	ENSRNOG00000031827	0.47738482	0.034935069	0.180849499
Vars1	ENSRNOG00000000867	0.477351138	0.014610938	0.108296107
Zfp830	ENSRNOG00000007578	0.475804997	0.044176358	0.204142982
Ppp2r5d	ENSRNOG00000016849	0.475622538	0.033040339	0.1756889
Kifbp	ENSRNOG00000000395	0.475212497	0.039622742	0.193821765
Dpp9	ENSRNOG00000050748	0.474727522	0.011758876	0.093958108
Psmd3	ENSRNOG00000028103	0.472695364	0.026569303	0.154996014
Ppp1r8	ENSRNOG00000059550	0.47120715	0.03775163	0.188364201
Zswim9	ENSRNOG00000014186	0.47083785	0.020304792	0.132142294
Wbp2	ENSRNOG00000007971	0.468383417	0.016584302	0.116487167
Rdm1	ENSRNOG00000020751	0.468349327	0.025612832	0.151326479
Aarsd1	ENSRNOG00000020658	0.468325785	0.020703989	0.133470862
Zfp775	ENSRNOG00000008362	0.467690465	0.042902037	0.201204026
Vps28	ENSRNOG00000014633	0.4676694	0.040088219	0.194753491
Stk40	ENSRNOG00000025679	0.467467763	0.047217865	0.211088481
Furin	ENSRNOG00000011352	0.467272516	0.009066511	0.07971571
Eif3g	ENSRNOG00000020619	0.466204418	0.044916988	0.205307064
Map3k4	ENSRNOG00000017419	0.465107334	0.035455611	0.182009365
Srp68	ENSRNOG00000009351	0.464672978	0.023588799	0.14429299
Nup210	ENSRNOG00000005390	0.464339969	0.033561334	0.176829752
Snrpc	ENSRNOG00000000493	0.461570276	0.038313517	0.189935101
Letm1	ENSRNOG00000016427	0.458810335	0.044922451	0.205307064
Nudt9	ENSRNOG00000002186	0.457484051	0.029821904	0.165759994
Ap2a1	ENSRNOG00000026243	0.457192888	0.023597054	0.14429299
Ccdc149	ENSRNOG00000024454	0.456153234	0.024088953	0.14574986
Zdhhc1	ENSRNOG00000017045	0.455606024	0.044987471	0.205530449
Fdps	ENSRNOG00000043377	0.452500482	0.009787855	0.083542691
Ubald1	ENSRNOG00000027368	0.452150473	0.046995827	0.210681663
Mon1a	ENSRNOG00000018502	0.448985334	0.035786144	0.182642627
Brap	ENSRNOG00000046497	0.446956589	0.042077499	0.199200073
Dync2i2	ENSRNOG00000015636	0.446378054	0.040512192	0.196018619
Bcorl1	ENSRNOG00000005076	0.444977278	0.015864994	0.114063964
Dnmbp	ENSRNOG00000050742	0.440512535	0.040734598	0.196423717
Ccdc136	ENSRNOG00000007100	0.438061968	0.042502631	0.200481681
Zfp641	ENSRNOG00000032447	0.437826968	0.046504084	0.209459097
Map2k7	ENSRNOG00000001047	0.437770292	0.034724836	0.180337649
Pip4k2c	ENSRNOG00000005138	0.437304108	0.049745552	0.217706658
Neu1	ENSRNOG00000032942	0.437168868	0.029677205	0.165553927
Atxn7l1	ENSRNOG00000010415	0.435268665	0.039288729	0.192793198
Sik3	ENSRNOG00000045931	0.433479424	0.024414138	0.147089045
Gtpbp1	ENSRNOG00000014634	0.433090475	0.016287225	0.115539975
Eif2b4	ENSRNOG00000005301	0.432923745	0.02106931	0.134808974
Fzr1	ENSRNOG00000004169	0.432524994	0.042865264	0.201178246
Ddrgk1	ENSRNOG00000021232	0.431885251	0.021217825	0.135203971
Gstp1	ENSRNOG00000018237	0.426917706	0.045199914	0.205978747
Arvcf	ENSRNOG00000001888	0.425566379	0.0160833	0.11473343
Tcof1	ENSRNOG00000026108	0.422446326	0.043962182	0.203897751

Mcm7	ENSRNOG00000001349	0.419617853	0.03685502	0.185653992
Taf4	ENSRNOG00000054497	0.414652402	0.00692175	0.06673321
Abcf1	ENSRNOG00000000799	0.412935903	0.039393897	0.192973853
Eif3d	ENSRNOG00000005804	0.410131316	0.041946321	0.198881217
Acaa1a	ENSRNOG00000032908	0.409712316	0.037086855	0.186331246
Ppme1	ENSRNOG00000017227	0.409641307	0.04082919	0.196691674
Pfdn2	ENSRNOG00000066932	0.408806919	0.043065885	0.201613578
Ern1	ENSRNOG00000012864	0.407123254	0.021402879	0.135882992
Hsf1	ENSRNOG00000021732	0.404951511	0.039744087	0.194049786
Farsa	ENSRNOG00000003149	0.404914662	0.037932887	0.188695521
Gga1	ENSRNOG00000008897	0.397488527	0.033372433	0.176491006
Pim3	ENSRNOG00000029698	0.396035968	0.03679318	0.185653992
Slfnl1	ENSRNOG00000032183	0.394781483	0.040077658	0.194753491
Elac2	ENSRNOG00000003424	0.393944299	0.022625667	0.140721715
Pacs2	ENSRNOG00000014744	0.392023873	0.038087813	0.189345284
Bcor	ENSRNOG00000034240	0.391379312	0.037382183	0.187247415
Naa60	ENSRNOG00000007280	0.389985121	0.037364543	0.187247415
Mllt6	ENSRNOG00000053285	0.388110983	0.042652043	0.200700341
Tyw1	ENSRNOG00000024352	0.375810372	0.040064819	0.194753491
Ndst1	ENSRNOG00000019014	0.375547275	0.020210806	0.131845633
Cep104	ENSRNOG00000025000	0.370610174	0.047701637	0.212127047
Ston2	ENSRNOG00000004458	0.368444461	0.044842604	0.205182898
Csnk1e	ENSRNOG00000013076	0.358708734	0.035734144	0.182642627
Relt	ENSRNOG00000025075	0.352025729	0.030304922	0.167014747
Casz1	ENSRNOG00000013474	0.345998647	0.017104247	0.118542781
Ipo4	ENSRNOG00000019553	0.342971732	0.04044093	0.195856293
Nolc1	ENSRNOG00000018704	0.32507087	0.035515144	0.182087617

Table S2. Differentially expressed genes (DEGs) down-regulated in DMDmdx vs WT rat skeletal muscl

GeneName	GeneStableID	log2FoldChange	pvalue	padj
Ankrd1	ENSRNOG00000018598	-5,573111	5.26E-34	2.05E-30
Myh3	ENSRNOG00000046276	-4,404011	1.18E-19	1.08E-16
Igf2	ENSRNOG00000020369	-4,380468	7.84E-46	1.22E-41
Lilrb4	ENSRNOG00000027811	-4,349634	1.72E-12	4.88E-10
Myh8	ENSRNOG00000068010	-4,316277	7.78E-24	1.10E-20
Spp1	ENSRNOG00000043451	-4,162578	3.16E-09	4.25E-07
Igfn1	ENSRNOG00000026087	-4,053071	0.000206296	0.005142543
Cd28	ENSRNOG00000010283	-4,034134	4.70E-22	5.23E-19
Rn5-8s	ENSRNOG00000070263	-3,619791	0.000485204	0.010025837
S100a4	ENSRNOG00000011821	-3,605824	1.50E-41	1.17E-37
Cdkn1a	ENSRNOG00000000521	-3,597172	1.82E-22	2.36E-19
Gpnmb	ENSRNOG00000008816	-3,467488	6.69E-15	2.89E-12
Timp1	ENSRNOG00000010208	-3,416434	2.69E-35	1.40E-31
Ly49si4	ENSRNOG00000053661	-3,364632	0.003254857	NA
Mt2A	ENSRNOG00000043098	-3,314023	0.004170079	0.047807091
Dlk1	ENSRNOG00000019584	-3,288725	3.31E-10	5.79E-08
Atp1b4	ENSRNOG00000007059	-3,243781	8.20E-10	1.25E-07
Snora2l1	ENSRNOG00000063116	-3,220082	0.000403884	NA
Sema3a	ENSRNOG00000023337	-3,204471	3.13E-14	1.13E-11
Thbs4	ENSRNOG00000012471	-3,201852	6.78E-19	5.03E-16
Fgl2	ENSRNOG00000012881	-3,037596	7.44E-15	3.13E-12
Slitrk4	ENSRNOG00000062783	-3,022500	2.32E-05	0.000890533
Tlr7	ENSRNOG00000004249	-2,969888	4.62E-09	5.94E-07
Pde7a	ENSRNOG00000013048	-2,943924	2.80E-28	7.27E-25
Clrn1	ENSRNOG00000042477	-2,931857	0.006995797	0.067239059
Dclk1	ENSRNOG00000032922	-2,930277	1.65E-31	5.14E-28
Slfn4	ENSRNOG00000057092	-2,893261	0.001210838	0.020090374
Snora33	ENSRNOG00000054517	-2,852785	0.019783965	NA
Cd163	ENSRNOG00000010253	-2,781317	1.12E-17	7.25E-15
Snord118	ENSRNOG00000056054	-2,757384	0.008722928	0.077654165
Bicd1	ENSRNOG00000036911	-2,712493	4.74E-11	9.77E-09
Lilrb3	ENSRNOG00000046683	-2,711310	4.65E-06	0.000240913
Actc1	ENSRNOG00000008536	-2,706752	5.71E-08	5.39E-06
Mt1	ENSRNOG00000025764	-2,673661	0.001115795	0.018732855
Myh15	ENSRNOG00000061038	-2,662596	2.88E-07	2.21E-05
Bgn	ENSRNOG00000055962	-2,624871	7.22E-24	1.10E-20
Gbp3	ENSRNOG00000036885	-2,611912	0.005222791	0.055373135
Ifitm1	ENSRNOG00000004273	-2,592175	6.59E-13	1.97E-10
Postn	ENSRNOG00000012660	-2,576962	6.81E-12	1.71E-09
Snora22c	ENSRNOG00000051585	-2,565292	0.003589965	0.043116056
Osmr	ENSRNOG00000033192	-2,521457	1.45E-09	2.11E-07
Apoe	ENSRNOG00000018454	-2,516247	6.58E-28	1.46E-24
C1qb	ENSRNOG00000012749	-2,497821	1.48E-14	5.93E-12
Rrad	ENSRNOG00000011901	-2,485406	0.004545973	0.050518019
Rgs1	ENSRNOG00000003895	-2,456832	0.011214624	NA
Tceal7	ENSRNOG00000037645	-2,436309	0.002426344	0.032392834
Ctxn3	ENSRNOG00000022957	-2,431027	3.27E-06	0.000178769

Hpgd	ENSRNOG00000010610	-2,419762	1.82E-05	0.000732655
Mrc1	ENSRNOG00000018251	-2,408695	1.52E-16	8.75E-14
Ms4a6a	ENSRNOG00000020991	-2,403754	3.80E-05	0.001374086
Ms4a4a	ENSRNOG00000050024	-2,396855	5.10E-11	1.03E-08
Ctss	ENSRNOG00000021157	-2,389606	2.73E-10	4.88E-08
Thbs1	ENSRNOG00000045829	-2,379435	5.17E-07	3.68E-05
Sat1	ENSRNOG00000003809	-2,373240	1.03E-06	6.73E-05
Sln	ENSRNOG00000009047	-2,371899	0.003538096	0.042698321
C4a	ENSRNOG00000066573	-2,364430	3.85E-07	2.85E-05
Serpina3a	ENSRNOG00000029949	-2,354673	0.011576221	0.092967794
Capn6	ENSRNOG00000004882	-2,345246	1.97E-09	2.79E-07
Snora4	ENSRNOG00000056026	-2,330347	0.00221854	0.030534322
Lyz2	ENSRNOG00000005825	-2,307116	1.66E-18	1.18E-15
Lgals3	ENSRNOG00000010645	-2,306992	1.89E-16	1.02E-13
Tnnt2	ENSRNOG00000033734	-2,295850	8.90E-05	0.002666038
Retnlg	ENSRNOG00000001943	-2,286853	0.019038372	NA
Klk1	ENSRNOG00000068378	-2,284391	0.000349033	0.007766196
Klrd1	ENSRNOG00000060246	-2,260117	0.00280048	NA
Sfrp4	ENSRNOG00000054957	-2,228917	1.63E-08	1.78E-06
Musk	ENSRNOG00000033567	-2,228832	1.04E-15	5.05E-13
Cdkn2a	ENSRNOG00000059837	-2,228689	0.015167575	0.111414813
Ly49si2	ENSRNOG00000064635	-2,225862	3.65E-07	2.71E-05
Mpeg1	ENSRNOG00000063244	-2,225340	1.53E-13	5.07E-11
Cdh26	ENSRNOG00000053332	-2,223713	0.000167464	0.004377653
Cfb	ENSRNOG00000051235	-2,216573	6.98E-14	2.42E-11
Cd53	ENSRNOG00000017874	-2,209387	5.39E-08	5.12E-06
Clic2	ENSRNOG00000000728	-2,207338	7.20E-17	4.32E-14
C1qc	ENSRNOG00000012804	-2,205465	2.05E-11	4.50E-09
Mnda	ENSRNOG00000003486	-2,201423	5.95E-06	0.000296024
Clec7a	ENSRNOG00000054251	-2,186706	0.004736129	NA
Ly49si3	ENSRNOG00000064957	-2,185545	0.030714245	NA
Col3a1	ENSRNOG00000003357	-2,181469	1.65E-08	1.79E-06
C1s	ENSRNOG00000011971	-2,178811	7.34E-16	3.69E-13
Pdpn	ENSRNOG00000014961	-2,168914	2.02E-08	2.14E-06
C4b	ENSRNOG00000070397	-2,156716	4.70E-10	7.71E-08
Cfh	ENSRNOG00000030715	-2,156477	1.54E-11	3.60E-09
Rgs18	ENSRNOG00000003959	-2,155803	9.70E-05	0.002863664
Prrg4	ENSRNOG00000022710	-2,141659	0.002041362	0.028913108
Tubb6	ENSRNOG00000018371	-2,132115	1.98E-19	1.63E-16
Ppp1r14c	ENSRNOG00000016368	-2,115041	1.78E-06	0.000109096
Col1a1	ENSRNOG00000003897	-2,091850	1.16E-05	0.000513754
Aldh1a2	ENSRNOG00000055049	-2,086474	1.50E-06	9.27E-05
Map1a	ENSRNOG00000014230	-2,085631	7.34E-14	2.49E-11
S100a10	ENSRNOG00000023226	-2,075752	2.11E-12	5.86E-10
Slfn13	ENSRNOG00000021412	-2,071714	8.90E-11	1.71E-08
Msr1	ENSRNOG00000012779	-2,066859	3.20E-05	0.001177016
C7	ENSRNOG00000061379	-2,065433	3.95E-11	8.44E-09
Mgp	ENSRNOG00000005695	-2,050472	2.75E-27	5.36E-24
Col1a2	ENSRNOG00000011292	-2,038410	3.16E-07	2.41E-05
Ccl7	ENSRNOG00000000239	-2,033701	0.000343606	0.007669609

Mfap4	ENSRNOG00000045683	-2,028586	4.09E-11	8.62E-09
Casq2	ENSRNOG00000016243	-1,999976	1.22E-09	1.81E-07
Cilp	ENSRNOG00000029911	-1,997632	4.81E-07	3.46E-05
Ms4a6bl	ENSRNOG00000050395	-1,990304	1.87E-06	0.000113084
Cybb	ENSRNOG00000003622	-1,983866	9.43E-09	1.12E-06
Myl4	ENSRNOG00000050675	-1,977488	7.55E-05	0.002316214
Vcan	ENSRNOG00000029212	-1,973732	6.36E-10	1.02E-07
Hdx	ENSRNOG00000037799	-1,963576	0.001068784	0.018139061
Sgk1	ENSRNOG00000011815	-1,946619	1.67E-07	1.38E-05
Stab1	ENSRNOG00000018434	-1,945451	4.35E-09	5.65E-07
Srpx2	ENSRNOG00000003715	-1,944606	5.02E-05	0.001691108
Col8a1	ENSRNOG00000039668	-1,941528	1.32E-10	2.51E-08
Plod2	ENSRNOG00000030183	-1,935441	1.32E-07	1.13E-05
Scd2	ENSRNOG00000046005	-1,933036	1.18E-05	0.000517784
Lpar4	ENSRNOG00000002428	-1,931348	0.000102855	0.002995279
Apobec1	ENSRNOG00000015411	-1,923984	5.72E-12	1.46E-09
Aoah	ENSRNOG00000054964	-1,920427	0.000742639	0.013923359
F13a1	ENSRNOG00000015957	-1,917129	4.99E-07	3.57E-05
Enc1	ENSRNOG00000016541	-1,912373	6.70E-05	0.002112082
Ptk2b	ENSRNOG00000027839	-1,909138	4.12E-07	3.00E-05
Vcam1	ENSRNOG00000014333	-1,908831	1.55E-11	3.60E-09
Dynap	ENSRNOG00000063474	-1,901414	0.002217217	0.030534322
Clec4a3	ENSRNOG00000010018	-1,900729	2.74E-07	2.13E-05
Ccr2	ENSRNOG00000063127	-1,895896	1.71E-05	0.000699421
Cd44	ENSRNOG00000006094	-1,893983	1.90E-08	2.04E-06
Smarca1	ENSRNOG00000003762	-1,888043	7.47E-06	0.000358406
Spon1	ENSRNOG00000058003	-1,886939	9.10E-08	8.15E-06
Clec10a	ENSRNOG00000018715	-1,871969	1.64E-08	1.79E-06
Adamts9	ENSRNOG00000023257	-1,871067	0.001219013	0.020161601
Tspo	ENSRNOG00000010549	-1,871039	2.00E-14	7.77E-12
Col14a1	ENSRNOG00000026415	-1,865251	1.44E-07	1.22E-05
Gpx8	ENSRNOG00000010461	-1,853385	1.41E-05	0.000601267
Tlr8	ENSRNOG00000045992	-1,843474	1.40E-05	0.000598994
Or2b4	ENSRNOG00000065474	-1,841535	0.001273232	0.020793452
Tmsb10	ENSRNOG00000064090	-1,840370	1.73E-19	1.50E-16
Ctla2a	ENSRNOG00000039971	-1,836381	1.72E-06	0.000105934
Myc	ENSRNOG00000004500	-1,834397	0.000336588	0.007556257
Nox4	ENSRNOG00000013925	-1,829662	6.13E-08	5.76E-06
Ccr5	ENSRNOG00000049115	-1,826427	2.25E-05	0.000866548
Snora52	ENSRNOG00000065361	-1,825993	0.042043389	0.19909909
Cebpd	ENSRNOG00000050869	-1,823720	7.14E-06	0.00034643
Pvr	ENSRNOG00000019202	-1,821697	2.11E-07	1.69E-05
Chi3l1	ENSRNOG00000053272	-1,819776	0.000506713	0.010387624
C1qa	ENSRNOG00000012807	-1,815583	7.17E-09	8.87E-07
Lum	ENSRNOG00000004610	-1,814644	2.11E-05	0.000833206
Myof	ENSRNOG00000016117	-1,799910	7.81E-10	1.22E-07
Pltp	ENSRNOG00000016488	-1,798532	7.94E-08	7.28E-06
Anxa1	ENSRNOG00000017469	-1,797868	1.94E-10	3.56E-08
Mymk	ENSRNOG00000006142	-1,797393	0.007994761	0.073735667
Abca1	ENSRNOG00000018126	-1,791629	1.01E-17	6.87E-15

Anxa2	ENSRNOG00000010362	-1,779101	5.76E-27	9.97E-24
Kcne5	ENSRNOG00000019176	-1,771399	0.023339988	0.143651291
Chl1	ENSRNOG00000045771	-1,770151	0.002887009	0.036959407
Igsf1	ENSRNOG00000007600	-1,769490	0.005766139	0.059297985
Antxr2	ENSRNOG00000000081	-1,768127	5.08E-16	2.64E-13
Emp1	ENSRNOG00000008676	-1,750505	4.25E-10	7.13E-08
Galnt15	ENSRNOG00000019718	-1,743019	3.88E-11	8.39E-09
Col5a2	ENSRNOG00000003736	-1,734540	7.58E-13	2.23E-10
Atf3	ENSRNOG00000003745	-1,720655	0.002388947	0.032111965
Folr2	ENSRNOG00000019890	-1,720257	2.39E-06	0.000138743
Nexmif	ENSRNOG00000021589	-1,716326	0.001309754	0.02127838
Mmrn1	ENSRNOG00000024986	-1,713805	0.007998285	0.073735667
Ncam1	ENSRNOG00000031890	-1,710234	1.59E-05	0.000655823
Fcgr2b	ENSRNOG00000046452	-1,710161	0.013045325	0.10076657
Cenpf	ENSRNOG00000003388	-1,710079	0.012317556	0.096776361
Aebp1	ENSRNOG00000013720	-1,709613	8.35E-08	7.56E-06
Bnc2	ENSRNOG00000006553	-1,709162	2.71E-06	0.000155292
Bcl2a1	ENSRNOG00000047606	-1,705697	0.005585156	0.057992791
Col6a1	ENSRNOG00000001249	-1,702640	1.09E-21	1.13E-18
Pcdh11x	ENSRNOG00000069840	-1,695227	0.009703653	0.083158921
Xdh	ENSRNOG00000007081	-1,695186	2.08E-08	2.19E-06
Piezo1	ENSRNOG00000056786	-1,692515	9.95E-07	6.54E-05
Chrne	ENSRNOG00000003777	-1,688342	0.001000438	0.017318697
Plekho2	ENSRNOG00000029242	-1,685522	1.00E-05	0.00045916
Gulp1	ENSRNOG00000003242	-1,685446	2.33E-06	0.00013611
Fgf7	ENSRNOG00000009425	-1,683815	1.44E-05	0.000611605
U3	ENSRNOG00000068470	-1,678700	0.031875881	NA
Vim	ENSRNOG00000018087	-1,677323	2.79E-19	2.18E-16
Eda2r	ENSRNOG00000013018	-1,670803	1.60E-10	3.01E-08
Cdkn2b	ENSRNOG00000006735	-1,668748	5.54E-07	3.89E-05
Ogn	ENSRNOG00000029792	-1,668264	1.05E-08	1.22E-06
Pf4	ENSRNOG00000028015	-1,667425	1.16E-06	7.49E-05
Vsir	ENSRNOG00000000569	-1,667385	4.82E-13	1.47E-10
Sfrp1	ENSRNOG00000017783	-1,666776	4.12E-06	0.000219756
Sbno2	ENSRNOG00000013987	-1,665772	0.004502772	0.050337407
Gria3	ENSRNOG00000007682	-1,664631	0.00045524	0.00959762
Errfi1	ENSRNOG00000058186	-1,662999	0.000121032	0.003416084
Pirb	ENSRNOG00000058422	-1,662417	0.003070516	0.038665036
Lrp1	ENSRNOG00000025053	-1,656436	3.26E-12	8.46E-10
Ctsk	ENSRNOG00000021155	-1,651051	3.29E-10	5.79E-08
Snord97	ENSRNOG00000069135	-1,646595	2.49E-05	0.000948177
Rab31	ENSRNOG00000042189	-1,642412	3.33E-09	4.43E-07
Adam19	ENSRNOG00000042980	-1,633779	2.72E-22	3.26E-19
Enpp3	ENSRNOG00000013791	-1,633014	8.26E-12	2.01E-09
Ncoa7	ENSRNOG00000014004	-1,627480	1.21E-05	0.000526635
Pdgfra	ENSRNOG00000002244	-1,627462	7.59E-12	1.88E-09
Ccl6	ENSRNOG00000030021	-1,623076	0.000208818	0.005171683
Apbb1ip	ENSRNOG00000017803	-1,621799	1.57E-08	1.74E-06
Cpne8	ENSRNOG00000026128	-1,621433	1.47E-05	0.000616455
Cxcl9	ENSRNOG00000022242	-1,619893	0.000245711	0.005862449

Clec12a	ENSRNOG00000054860	-1,608427	0.006130624	0.062063105
Sp140	ENSRNOG00000022800	-1,608349	5.92E-05	0.001909212
Col6a3	ENSRNOG00000019648	-1,600796	2.04E-11	4.50E-09
Klf4	ENSRNOG00000016299	-1,600381	0.000288017	0.006647862
Fos	ENSRNOG00000008015	-1,597517	0.001680014	0.025439066
Cfap54	ENSRNOG00000024494	-1,596423	0.03502128	0.180988087
Clec4a1	ENSRNOG00000042139	-1,594557	0.000425936	0.009115488
Chrna1	ENSRNOG00000018286	-1,594089	0.003481721	0.042184706
Col19a1	ENSRNOG00000012759	-1,589228	0.0001303	0.003644652
Sned1	ENSRNOG00000023548	-1,588470	0.000318859	0.007241735
Srgap2	ENSRNOG00000006733	-1,585629	7.66E-09	9.32E-07
Herc6	ENSRNOG00000023969	-1,584047	0.003030452	0.038261299
Ccl2	ENSRNOG00000007159	-1,581807	0.003122505	0.038934239
Gria4	ENSRNOG00000006957	-1,574152	6.95E-06	0.000338982
Fyb1	ENSRNOG00000013886	-1,571739	0.001340608	0.021626248
Map2	ENSRNOG00000011841	-1,566238	0.001090068	0.018420013
Ptx3	ENSRNOG00000012280	-1,564007	0.006346058	0.06337922
Tiam2	ENSRNOG00000016728	-1,563716	0.000919586	0.01622553
Slc10a6	ENSRNOG00000002057	-1,560118	0.005578779	0.057992791
Fcgr1a	ENSRNOG00000021199	-1,555156	0.000764695	0.014161273
Fap	ENSRNOG00000005679	-1,554876	5.24E-11	1.05E-08
Ccnl1	ENSRNOG00000011586	-1,554599	0.000845159	0.015257919
Tc2n	ENSRNOG00000004754	-1,546648	0.000106084	0.003062283
Smoc2	ENSRNOG00000014166	-1,546325	4.77E-11	9.77E-09
Runx1	ENSRNOG00000001704	-1,545538	6.55E-05	0.002073656
Gatm	ENSRNOG00000000168	-1,544446	1.18E-07	1.02E-05
Mt3	ENSRNOG00000018958	-1,539576	0.016293535	0.115539975
Bid	ENSRNOG00000012439	-1,536919	6.31E-06	0.00031292
Mki67	ENSRNOG00000028137	-1,536299	0.000207945	0.00516711
Fhl2	ENSRNOG00000016866	-1,534343	3.81E-10	6.46E-08
Hgf	ENSRNOG00000007027	-1,534171	0.001203626	0.019991989
Clu	ENSRNOG00000016460	-1,532822	1.04E-08	1.22E-06
Aspn	ENSRNOG00000015410	-1,530299	8.81E-06	0.00040981
Pcolce	ENSRNOG00000025001	-1,525749	2.67E-07	2.09E-05
Vat1	ENSRNOG00000020684	-1,525611	8.46E-15	3.47E-12
Hsd11b1	ENSRNOG00000005861	-1,521403	4.71E-08	4.53E-06
Csf2rb	ENSRNOG00000000187	-1,513250	0.001688323	0.025488445
Gda	ENSRNOG00000018282	-1,510050	1.68E-10	3.12E-08
Fermt3	ENSRNOG00000021161	-1,508795	0.001611879	0.024790793
Dnm1	ENSRNOG00000033835	-1,505304	6.35E-05	0.002024498
Osbpl3	ENSRNOG00000010011	-1,504424	7.19E-08	6.66E-06
Plekhh2	ENSRNOG00000005124	-1,501856	4.39E-05	0.001531317
Cd300le	ENSRNOG00000042825	-1,501451	0.000615982	0.012041412
Cdk14	ENSRNOG00000007151	-1,496099	3.76E-10	6.44E-08
Col6a2	ENSRNOG00000001254	-1,491332	5.92E-15	2.64E-12
Pi15	ENSRNOG00000017686	-1,490626	0.000331652	0.007479318
Samd9	ENSRNOG00000052444	-1,490516	0.001669142	0.025321547
Bmp1	ENSRNOG00000010890	-1,486574	3.88E-07	2.85E-05
Abca9	ENSRNOG00000059326	-1,483042	1.65E-11	3.76E-09
Igsf10	ENSRNOG00000013917	-1,482219	0.007690191	0.071658602

Tnfaip6	ENSRNOG00000050792	-1,476424	0.011435533	0.092122859
Plau	ENSRNOG00000010516	-1,471858	3.99E-07	2.92E-05
Tnip2	ENSRNOG00000013805	-1,470847	0.001935336	0.028022801
Ldlr	ENSRNOG00000009946	-1,468079	6.99E-10	1.11E-07
Atp10a	ENSRNOG00000056228	-1,467386	0.000476434	0.00994068
Zfp36	ENSRNOG00000058388	-1,466518	0.000527809	0.010735341
Il1r1	ENSRNOG00000014504	-1,466192	1.18E-08	1.36E-06
Snora73bl1	ENSRNOG00000052439	-1,465236	0.001323349	0.021454503
Fam171b	ENSRNOG00000004783	-1,463705	2.51E-05	0.000953923
C1r	ENSRNOG00000011796	-1,463122	8.74E-09	1.05E-06
Tmeff2	ENSRNOG00000016623	-1,460782	5.08E-05	0.001705257
Nes	ENSRNOG00000018681	-1,459200	0.000194768	0.00492612
Klf5	ENSRNOG00000008785	-1,459159	0.000500045	0.010305167
Ms4a7	ENSRNOG00000020953	-1,458405	0.043592948	0.203030868
Diaph2	ENSRNOG00000057826	-1,458104	8.99E-05	0.002681968
Vav3	ENSRNOG00000020485	-1,456521	1.09E-07	9.62E-06
Ptprc	ENSRNOG00000000655	-1,455800	1.12E-05	0.000500817
Lama2	ENSRNOG00000011134	-1,451093	3.02E-12	7.97E-10
Mmp2	ENSRNOG00000016695	-1,448278	1.53E-07	1.29E-05
Npnt	ENSRNOG00000052873	-1,442849	7.62E-06	0.000362923
Atp8b1	ENSRNOG00000024952	-1,441261	0.000178847	0.004618601
Ccdc88a	ENSRNOG00000004057	-1,440651	2.29E-06	0.000135194
Csgalnact1	ENSRNOG00000013024	-1,436042	2.78E-06	0.000156966
Gxylt2	ENSRNOG00000005621	-1,429702	0.001352244	0.021672562
Gadd45a	ENSRNOG00000005615	-1,427141	1.22E-06	7.79E-05
Zfp182	ENSRNOG00000047132	-1,426070	5.38E-05	0.001785968
Anxa5	ENSRNOG00000014453	-1,425304	7.94E-21	7.73E-18
Rn18s	ENSRNOG00000068307	-1,423315	0.012225641	0.096362942
Lyve1	ENSRNOG00000063592	-1,421749	0.001927068	0.027981095
Ryr3	ENSRNOG00000006645	-1,420238	5.82E-05	0.001892418
Me2	ENSRNOG00000015582	-1,419349	1.22E-07	1.05E-05
Tcim	ENSRNOG00000047977	-1,416603	0.001353492	0.021672562
Oasl2	ENSRNOG00000028814	-1,416161	0.011034867	0.089839098
Tuba1c	ENSRNOG00000021438	-1,413955	8.58E-09	1.04E-06
Egr1	ENSRNOG00000019422	-1,411876	0.000247666	0.005891059
Hmcn2	ENSRNOG00000064166	-1,410835	3.79E-06	0.000203397
Top2a	ENSRNOG00000053047	-1,409353	0.01175984	0.093958108
Pcare	ENSRNOG00000008942	-1,407038	0.00185184	0.027116221
Ecm2	ENSRNOG00000031716	-1,402629	3.19E-07	2.41E-05
Tyrobp	ENSRNOG00000020845	-1,399800	0.002203221	0.030399341
Fibin	ENSRNOG00000004699	-1,398564	0.009570604	0.082435464
Igf1	ENSRNOG00000004517	-1,395836	4.26E-07	3.09E-05
Tec	ENSRNOG00000002278	-1,393276	0.000106335	0.003062283
Tent5a	ENSRNOG00000010240	-1,393061	0.000273148	0.006399466
Fcer1g	ENSRNOG00000024159	-1,389758	0.00910659	0.079753047
Snx10	ENSRNOG00000011944	-1,387182	1.98E-05	0.000787612
Chrnd	ENSRNOG00000019527	-1,386662	0.003171672	0.039437077
Gpx3	ENSRNOG00000052564	-1,386520	9.01E-07	5.97E-05
Col5a1	ENSRNOG00000008749	-1,385885	1.88E-09	2.71E-07
Chrd	ENSRNOG00000001750	-1,382434	0.000192751	0.0048991

Col5a3	ENSRNOG00000020525	-1,382357	3.53E-10	6.12E-08
Mill1	ENSRNOG00000017699	-1,382277	0.015973927	0.114372565
Tceal5	ENSRNOG00000046280	-1,380239	0.016045313	0.11461989
Ftl1	ENSRNOG00000064385	-1,375826	2.17E-13	7.06E-11
Nucb2	ENSRNOG00000020456	-1,374107	2.53E-06	0.000146817
Serping1	ENSRNOG00000007457	-1,371219	2.33E-06	0.00013611
Scn7a	ENSRNOG00000029342	-1,370717	2.00E-10	3.62E-08
Nfkbiz	ENSRNOG00000031163	-1,369624	0.00370954	0.044084383
Slc9a9	ENSRNOG00000008554	-1,365246	5.29E-05	0.001773247
Otud1	ENSRNOG00000016950	-1,361413	0.00220483	0.030399341
Ripk1	ENSRNOG00000017787	-1,361229	0.000462626	0.009727011
Pros1	ENSRNOG00000048723	-1,361114	3.53E-08	3.52E-06
Utrn	ENSRNOG00000011058	-1,359737	1.04E-05	0.000468089
Colec12	ENSRNOG00000016366	-1,359591	1.41E-05	0.000601409
Cyp1b1	ENSRNOG00000040287	-1,358616	0.012673556	0.09877639
Ccdc80	ENSRNOG00000002052	-1,355498	2.42E-05	0.000922965
Cd200	ENSRNOG00000002141	-1,354839	2.26E-09	3.17E-07
Ddit4	ENSRNOG00000057078	-1,352603	0.00032161	0.007293578
Snora5c	ENSRNOG00000057537	-1,348219	0.044047979	0.204001135
Col6a6	ENSRNOG00000023007	-1,347527	0.000103707	0.003014465
Scn3b	ENSRNOG00000057221	-1,346218	0.004223533	0.048136535
Syk	ENSRNOG00000012160	-1,343846	0.001837204	0.026983494
Rp2	ENSRNOG00000025428	-1,343049	1.76E-05	0.000713633
Myf5	ENSRNOG00000004768	-1,337319	0.006911305	0.066673762
Plcb1	ENSRNOG00000004810	-1,336805	3.22E-06	0.000177077
Ip6k3	ENSRNOG00000025883	-1,335216	5.38E-05	0.001785968
Irak3	ENSRNOG00000004226	-1,334686	4.74E-06	0.000244702
Lvrn	ENSRNOG00000003950	-1,332453	0.000102218	0.002987896
Xirp1	ENSRNOG00000037085	-1,329396	0.002240372	0.030744173
Zbtb16	ENSRNOG00000029980	-1,326853	0.000818411	0.01484226
Selenop	ENSRNOG00000053086	-1,325585	4.06E-05	0.00144938
Lcp2	ENSRNOG00000005620	-1,324788	0.002104303	0.029442563
Cp	ENSRNOG00000011913	-1,320854	9.19E-07	6.07E-05
Soat1	ENSRNOG00000004111	-1,318829	9.18E-05	0.002734091
Elmo1	ENSRNOG00000059705	-1,313985	5.41E-06	0.000273651
Apold1	ENSRNOG00000007830	-1,312706	0.006609077	0.064883061
Sh3bgrl	ENSRNOG00000069303	-1,311335	4.60E-06	0.00023899
Nav3	ENSRNOG00000052157	-1,311269	5.17E-06	0.000263874
Resf1	ENSRNOG00000036913	-1,307586	2.25E-06	0.000133096
Rarres1	ENSRNOG00000037853	-1,306860	0.003414987	0.041689072
Mx1	ENSRNOG00000001959	-1,305750	0.041363691	0.197380187
Itgbl1	ENSRNOG00000004516	-1,304304	3.54E-05	0.00129223
Bclaf3	ENSRNOG00000005135	-1,303921	0.000903636	0.016053192
Slfn2	ENSRNOG00000037113	-1,302851	9.88E-06	0.000452882
Tgfb1	ENSRNOG00000020652	-1,300569	2.03E-06	0.000122249
Stom	ENSRNOG00000019147	-1,300080	1.04E-08	1.22E-06
Axl	ENSRNOG00000020716	-1,299321	7.35E-10	1.16E-07
Scpep1	ENSRNOG00000002358	-1,297370	2.34E-14	8.89E-12
Arhgap4	ENSRNOG00000058545	-1,296848	0.000434728	0.009278167
Dcn	ENSRNOG00000004554	-1,292229	0.000151629	0.004080097

H19	ENSRNOG00000062678	-1,291969	0.011503141	0.092476232
Adamts15	ENSRNOG00000009892	-1,289453	1.05E-06	6.86E-05
Ptbp3	ENSRNOG00000016334	-1,286647	9.32E-08	8.25E-06
Samd9l	ENSRNOG00000063679	-1,286048	0.000178257	0.004618601
Abi3bp	ENSRNOG00000001627	-1,283565	3.58E-15	1.64E-12
Naaladl2	ENSRNOG00000065021	-1,281561	0.000213063	0.005252403
Gsap	ENSRNOG00000028801	-1,281318	0.000549042	0.011074855
Litaf	ENSRNOG00000002520	-1,280160	4.79E-05	0.001633764
Lpar1	ENSRNOG00000013656	-1,278841	3.80E-05	0.001374086
Ctsb	ENSRNOG00000010331	-1,278696	1.10E-05	0.000491584
Il17ra	ENSRNOG00000011153	-1,277227	7.04E-05	0.002196044
Ier5	ENSRNOG00000059406	-1,277009	1.45E-06	9.09E-05
Cpq	ENSRNOG00000005931	-1,276440	4.09E-05	0.00145136
Adamts12	ENSRNOG00000018865	-1,275690	4.90E-08	4.68E-06
Sik1	ENSRNOG00000001189	-1,275641	0.000686878	0.013098612
Dse	ENSRNOG00000000824	-1,271093	4.07E-05	0.00144938
Mxra8	ENSRNOG00000019244	-1,270527	7.43E-11	1.45E-08
Cdk6	ENSRNOG00000009258	-1,269393	0.000330981	0.007479318
Casp12	ENSRNOG00000033434	-1,269292	7.50E-05	0.002306172
Slc25a24	ENSRNOG00000020470	-1,264208	3.64E-09	4.81E-07
Col11a1	ENSRNOG00000023148	-1,263466	0.012198539	0.096278239
Aif1	ENSRNOG00000000853	-1,260977	0.000317978	0.007232254
Ppic	ENSRNOG00000017416	-1,260159	0.000153561	0.004110796
Ikbke	ENSRNOG00000025100	-1,258053	0.010637371	0.087983603
Ahr	ENSRNOG00000004342	-1,257848	3.93E-09	5.15E-07
Ednrb	ENSRNOG00000010997	-1,256120	0.000682621	0.013057378
Efemp1	ENSRNOG00000003553	-1,254405	0.000738649	0.013865239
Mlkl	ENSRNOG00000042353	-1,253774	0.001423658	0.022541256
Ehd4	ENSRNOG00000007584	-1,253611	1.28E-09	1.89E-07
Tp53inp1	ENSRNOG00000007964	-1,252728	1.03E-05	0.000467111
Rpl3	ENSRNOG00000016896	-1,251465	8.13E-10	1.25E-07
Myo1f	ENSRNOG00000008409	-1,250919	0.00104681	0.017896485
Cd82	ENSRNOG00000000047	-1,250449	0.000150999	0.004075896
Fchsd2	ENSRNOG00000019319	-1,250172	1.84E-06	0.000111951
Boc	ENSRNOG00000002041	-1,250006	4.55E-07	3.28E-05
Btk	ENSRNOG00000052407	-1,249812	0.040188512	0.195119046
Myo1b	ENSRNOG00000048152	-1,249361	0.00010514	0.003050436
Blnk	ENSRNOG00000013967	-1,248890	0.040205576	0.195141082
Il18	ENSRNOG00000009848	-1,248604	0.01128236	0.091219084
Atp8b4	ENSRNOG00000031598	-1,247218	0.027488279	0.158148963
Sf3a3	ENSRNOG00000007629	-1,244394	0.000580515	0.011565765
Lox	ENSRNOG00000014426	-1,242734	0.000374044	0.008235764
Fmnl1	ENSRNOG00000003207	-1,242621	4.28E-06	0.000226987
Ccl21	ENSRNOG00000034290	-1,242329	0.003602282	0.043116056
Cotl1	ENSRNOG00000016257	-1,241382	0.000737508	0.013860517
Snx29	ENSRNOG00000002294	-1,239613	0.001724371	0.025841052
Ccdc141	ENSRNOG00000012580	-1,238652	1.11E-09	1.66E-07
Lcp1	ENSRNOG00000010319	-1,238079	3.41E-06	0.000184903
Rab27b	ENSRNOG00000012176	-1,236929	0.037885557	0.188676961
Itgam	ENSRNOG00000019728	-1,235672	0.006189738	0.062374932

Ltbp1	ENSRNOG00000033090	-1,235447	5.88E-07	4.09E-05
Grhl1	ENSRNOG00000054989	-1,233464	0.036783872	0.185653992
Loxl1	ENSRNOG00000008680	-1,233390	1.13E-05	0.000504129
Snord17	ENSRNOG00000052573	-1,233157	0.00491397	0.053203373
Lgmn	ENSRNOG00000007089	-1,231823	3.60E-08	3.57E-06
CXHXorf65	ENSRNOG00000003954	-1,231667	0.00326911	0.040358747
Thbs2	ENSRNOG00000010529	-1,230225	0.000162768	0.004283652
Aig1	ENSRNOG00000010753	-1,229945	5.88E-05	0.00190317
Prdm5	ENSRNOG00000023679	-1,228628	4.00E-05	0.001437038
Ifi47	ENSRNOG00000002470	-1,227832	0.003550181	0.042791047
Pi4k2b	ENSRNOG00000003924	-1,227304	5.53E-05	0.00181958
Mtmr11	ENSRNOG00000021176	-1,227095	1.58E-05	0.00065543
Bmper	ENSRNOG00000015357	-1,226504	2.88E-07	2.21E-05
Rnu105c	ENSRNOG00000052528	-1,226383	0.025952695	0.152812923
Serp1	ENSRNOG00000011763	-1,225465	1.82E-06	0.000111246
Adap2	ENSRNOG00000037148	-1,225213	0.000403738	0.008724332
Rubcnl	ENSRNOG00000022760	-1,224803	0.020531163	0.133131501
Arhgap26	ENSRNOG00000013920	-1,224436	0.005450423	0.057068275
Cyth4	ENSRNOG00000007679	-1,223575	0.00122107	0.020174197
Rab32	ENSRNOG00000014258	-1,222993	0.000166621	0.004362939
Eln	ENSRNOG00000001469	-1,222968	2.90E-13	9.22E-11
Igf2bp2	ENSRNOG00000025946	-1,220533	0.045212006	0.205978747
Ap1s2	ENSRNOG00000038686	-1,218852	0.002364334	0.03192056
Sytl2	ENSRNOG00000030776	-1,217678	2.67E-07	2.09E-05
Ms4a6b	ENSRNOG00000047349	-1,216173	0.005563145	0.057898332
Gabrr2	ENSRNOG00000007490	-1,214993	0.020977728	0.134450066
Man2a1	ENSRNOG00000015439	-1,212895	1.32E-12	3.82E-10
Ripk3	ENSRNOG00000020465	-1,212247	0.012433667	0.097541056
Abcg3l3	ENSRNOG00000069246	-1,209763	3.04E-05	0.001124115
Eci3	ENSRNOG00000029549	-1,208811	3.91E-05	0.001411454
Nr4a2	ENSRNOG00000005600	-1,206497	0.011153057	0.090644039
Fat1	ENSRNOG00000030954	-1,206363	1.17E-11	2.81E-09
Nrbp2	ENSRNOG00000029535	-1,206318	1.77E-05	0.000714933
Rpgr	ENSRNOG00000003013	-1,202384	4.78E-05	0.001631568
Dmd	ENSRNOG00000046366	-1,202055	0.001744389	0.026057128
Prpf39	ENSRNOG00000004521	-1,201901	0.001235048	0.020340429
Klhl5	ENSRNOG00000008421	-1,201563	0.000146327	0.003971746
Fes	ENSRNOG00000011683	-1,201342	0.012840235	0.099626922
Per1	ENSRNOG00000007387	-1,201014	0.029755101	0.165565884
Erbb3	ENSRNOG00000004964	-1,200584	0.016839883	0.117441978
Plagl1	ENSRNOG00000025587	-1,200095	0.010131236	0.08509146
Gja1	ENSRNOG00000000805	-1,199551	2.90E-06	0.000162354
Parp9	ENSRNOG00000023463	-1,198157	0.000572046	0.011426252
Csf1r	ENSRNOG00000018414	-1,197543	5.70E-06	0.000285373
Rab13	ENSRNOG00000016733	-1,194354	0.015932599	0.114286323
Klra2	ENSRNOG00000031515	-1,192223	0.000530646	0.010752187
Zfp945	ENSRNOG00000061533	-1,191747	2.22E-05	0.000859866
Gask1b	ENSRNOG00000010183	-1,191646	4.40E-05	0.001531317
Man1a1	ENSRNOG00000000800	-1,190932	1.66E-11	3.76E-09
Pak1	ENSRNOG00000029784	-1,190567	0.004205673	0.048064247

Calcrl	ENSRNOG00000054695	-1,188890	3.08E-06	0.000170092
Dpt	ENSRNOG00000002947	-1,187511	5.89E-06	0.000294101
Abcg1	ENSRNOG00000001158	-1,186645	0.004027588	0.046688858
Pkd2	ENSRNOG00000002146	-1,186559	1.48E-08	1.67E-06
Ptpn12	ENSRNOG00000013135	-1,186182	2.34E-05	0.000895759
Marcks	ENSRNOG00000000579	-1,185881	3.88E-06	0.000207784
Klf2	ENSRNOG00000067705	-1,185789	0.002344663	0.031704113
Zfc3h1	ENSRNOG00000053060	-1,184606	0.000224413	0.005420717
Gabre	ENSRNOG00000061182	-1,183820	0.016569751	0.116487167
Lmo2	ENSRNOG00000009401	-1,181204	3.52E-06	0.000189512
Ifitm3	ENSRNOG00000015078	-1,181168	3.46E-08	3.48E-06
Itm2c	ENSRNOG00000017359	-1,181109	2.53E-07	2.00E-05
Cers6	ENSRNOG00000024595	-1,180049	0.003208553	0.039800361
Adamts2	ENSRNOG00000061484	-1,179705	7.29E-07	4.90E-05
Adamts1	ENSRNOG00000001607	-1,179219	0.001329026	0.021501784
Rrbp1	ENSRNOG00000005958	-1,175749	2.75E-05	0.0010336
Cep112	ENSRNOG00000024557	-1,173773	0.004873854	0.052916132
Twf1	ENSRNOG00000022507	-1,172442	2.23E-06	0.000133096
Dnm3	ENSRNOG00000026490	-1,172198	0.000508098	0.010392917
Fmnl2	ENSRNOG00000055567	-1,171537	0.00175853	0.026218083
Arhgef2	ENSRNOG00000020027	-1,170453	3.10E-08	3.16E-06
Plxdc2	ENSRNOG00000000142	-1,169963	8.80E-08	7.93E-06
Arap2	ENSRNOG00000056826	-1,167751	0.002400236	0.032182168
Sh3pxd2b	ENSRNOG00000004063	-1,167678	1.46E-05	0.000616455
Aff2	ENSRNOG00000052572	-1,166112	0.017214427	0.118842152
Stxbp5	ENSRNOG00000013351	-1,165956	4.14E-05	0.001463091
Ifi27l2b	ENSRNOG00000026605	-1,165242	0.004390809	0.049356997
Hs6st2	ENSRNOG00000030880	-1,164595	0.019166331	0.127939777
Peg3	ENSRNOG00000014791	-1,164225	1.44E-08	1.63E-06
Mctp2	ENSRNOG00000009932	-1,163436	0.021772326	0.137077168
Gvin1	ENSRNOG00000045560	-1,163303	0.000279452	0.006488621
Nckap1l	ENSRNOG00000036829	-1,163244	0.001377183	0.021939171
Plp2	ENSRNOG00000039496	-1,163194	0.000590055	0.011696009
Adamts10	ENSRNOG00000008857	-1,162900	0.001497975	0.023361818
F3	ENSRNOG00000011800	-1,162292	0.000655416	0.012637842
Medag	ENSRNOG00000000906	-1,161838	0.000146085	0.003971746
Wsb1	ENSRNOG00000012929	-1,161379	2.00E-06	0.00012082
Oas1k	ENSRNOG00000062846	-1,160732	0.01429412	0.106786393
Sdcbp	ENSRNOG00000009683	-1,160623	1.90E-05	0.000762034
B2m	ENSRNOG00000017123	-1,160205	0.000262516	0.006206363
Fam114a1	ENSRNOG00000026504	-1,160117	1.42E-06	8.91E-05
Klhl2	ENSRNOG00000029441	-1,159137	0.00026579	0.006255309
Nectin3	ENSRNOG00000002176	-1,159014	2.33E-06	0.00013611
Dock11	ENSRNOG00000013321	-1,158917	0.000163464	0.00429472
Klf6	ENSRNOG00000016885	-1,158486	0.000146884	0.003979917
Slc43a3	ENSRNOG00000061768	-1,156835	0.006252491	0.062726216
Rgl1	ENSRNOG00000002347	-1,154946	2.21E-05	0.000855414
Sp110	ENSRNOG00000033747	-1,153936	0.000929865	0.016351353
Adamtsl2	ENSRNOG00000027742	-1,153555	0.001930825	0.027983486
Rgs10	ENSRNOG00000042592	-1,153426	4.32E-05	0.001512063

Macf1	ENSRNOG00000016047	-1,152049	4.34E-05	0.001514624
Scara5	ENSRNOG00000014398	-1,151767	5.66E-05	0.001844376
Snrnp70	ENSRNOG00000020763	-1,150860	0.000190326	0.004861109
Pdk3	ENSRNOG00000012513	-1,150728	0.004266084	0.048479639
Far1	ENSRNOG00000013176	-1,148656	4.74E-05	0.001624554
Chml	ENSRNOG00000067369	-1,148511	0.000374257	0.008235764
S100a11	ENSRNOG00000010105	-1,148279	1.40E-05	0.000598994
Ptpre	ENSRNOG00000015717	-1,148115	4.49E-05	0.001555217
Cntln	ENSRNOG00000043151	-1,148105	0.004708219	0.051730646
Tcaf1	ENSRNOG00000017911	-1,147647	6.40E-05	0.002029754
Snora28l1	ENSRNOG00000055908	-1,143552	0.049002033	0.215438898
Fam13b	ENSRNOG00000020384	-1,143446	4.84E-05	0.001647043
P3h3	ENSRNOG00000071218	-1,142295	5.58E-06	0.000281557
Spred1	ENSRNOG00000070996	-1,142066	8.59E-10	1.30E-07
Gch1	ENSRNOG00000011039	-1,141570	0.033434936	0.176649188
Zdhhc15	ENSRNOG00000002751	-1,141442	0.023877209	0.145252898
Otulinl	ENSRNOG00000012109	-1,139434	0.019664494	0.130131458
Plxna1	ENSRNOG00000017003	-1,138433	1.55E-07	1.30E-05
Lsp1	ENSRNOG00000020300	-1,138378	0.000419946	0.009012064
Iqgap2	ENSRNOG00000025406	-1,138239	0.002894631	0.036996183
Spata6l	ENSRNOG00000015137	-1,138108	0.044762109	0.204995198
Casp3	ENSRNOG00000010475	-1,137544	2.61E-05	0.000987794
Sertad4	ENSRNOG00000060773	-1,136020	1.02E-05	0.00046191
Arhgap10	ENSRNOG00000013152	-1,134696	1.89E-05	0.000757416
Gpr34	ENSRNOG00000039759	-1,133323	0.010105258	0.08496488
Ckap4	ENSRNOG00000008016	-1,132516	0.000614371	0.012040824
Fosb	ENSRNOG00000046667	-1,130512	0.014311296	0.106786393
Il4r	ENSRNOG00000015441	-1,130245	0.000751025	0.014013146
Coq8b	ENSRNOG00000020848	-1,129029	0.009078995	0.07973064
Iqgap1	ENSRNOG00000012002	-1,128083	0.000114775	0.003257187
Pla2r1	ENSRNOG00000008129	-1,128082	0.011188404	0.090777931
Antxr1	ENSRNOG00000008678	-1,127904	8.87E-07	5.91E-05
Fn1	ENSRNOG00000014288	-1,127325	2.12E-05	0.000837268
Slc51a	ENSRNOG00000001765	-1,127141	0.008456115	0.076022086
Anpep	ENSRNOG00000014610	-1,126243	0.000591499	0.011709724
Taf1d	ENSRNOG00000010921	-1,125780	3.48E-06	0.000188092
Arhgap23	ENSRNOG00000022771	-1,123534	0.009748709	0.08336163
Col18a1	ENSRNOG00000001229	-1,119426	1.75E-05	0.000708233
Pmp22	ENSRNOG00000003338	-1,118194	0.035268323	0.181445185
Card6	ENSRNOG00000001271	-1,118183	0.000196407	0.004937273
Vopp1	ENSRNOG00000006646	-1,118037	0.01167224	0.093497945
Ubxn2b	ENSRNOG00000071045	-1,116812	0.005108332	0.054624446
Cald1	ENSRNOG00000010233	-1,114787	3.46E-08	3.48E-06
Fbln2	ENSRNOG00000007338	-1,113413	1.57E-05	0.000653505
Alcam	ENSRNOG00000001989	-1,113364	0.001480321	0.023156025
Sp100	ENSRNOG00000022769	-1,113125	1.17E-05	0.000514194
Msn	ENSRNOG00000030118	-1,112416	3.62E-07	2.70E-05
Rftn2	ENSRNOG00000015594	-1,112182	0.00122684	0.020248053
Fkbp10	ENSRNOG00000015941	-1,110718	3.65E-05	0.001326468
Tm4sf1	ENSRNOG00000015812	-1,110073	1.16E-07	1.01E-05

Hmox1	ENSRNOG00000014117	-1,109843	0.023528474	0.144093406
Emp3	ENSRNOG00000021104	-1,107801	0.000453252	0.00956866
Fancb	ENSRNOG00000003346	-1,106574	0.01190573	0.094569329
Dab2	ENSRNOG00000028930	-1,106241	9.28E-08	8.25E-06
Cspp1	ENSRNOG00000021718	-1,105092	0.001444215	0.022728153
Pnp	ENSRNOG00000009982	-1,104179	5.16E-09	6.58E-07
Spata6	ENSRNOG00000007568	-1,103387	0.007662391	0.071442285
Tlr4	ENSRNOG00000010522	-1,103325	0.001773328	0.026363029
Cit	ENSRNOG00000001143	-1,103202	0.002663504	0.034901085
Casp1	ENSRNOG00000007372	-1,102784	0.000587535	0.011660885
Bicc1	ENSRNOG00000000614	-1,102377	4.32E-10	7.15E-08
Ttc9	ENSRNOG00000007254	-1,101185	0.004520028	0.050337407
Arpc1b	ENSRNOG00000000991	-1,101099	7.24E-07	4.88E-05
Id1	ENSRNOG00000021750	-1,100838	0.001054474	0.017994206
Tspan13	ENSRNOG00000005046	-1,096790	0.006535415	0.064419487
Kctd12	ENSRNOG00000066973	-1,096652	1.47E-06	9.17E-05
S100a6	ENSRNOG00000011647	-1,096565	0.000279193	0.006488621
Snora23	ENSRNOG00000057923	-1,095745	0.042696341	0.200848126
Tgfbr3	ENSRNOG00000002093	-1,095643	8.00E-08	7.29E-06
Dock10	ENSRNOG00000053200	-1,092944	3.19E-05	0.00117442
Cyria	ENSRNOG00000052758	-1,092679	1.52E-05	0.000635358
Dennd2c	ENSRNOG00000018716	-1,091712	1.90E-07	1.55E-05
Casp8	ENSRNOG00000012331	-1,091649	1.14E-05	0.000505271
Scarb1	ENSRNOG00000000981	-1,090261	0.001163606	0.019368563
Abhd2	ENSRNOG00000017120	-1,086975	8.84E-05	0.002655841
Heph	ENSRNOG00000012294	-1,086836	0.001877978	0.027439002
Leng8	ENSRNOG00000018642	-1,086333	0.013170226	0.101456721
Mat2a	ENSRNOG00000013520	-1,086280	0.000374826	0.008236649
Epb41l1	ENSRNOG00000057817	-1,085978	7.24E-07	4.88E-05
Plxna3	ENSRNOG00000060464	-1,085448	0.00196282	0.028315502
Pgrmc1	ENSRNOG00000012786	-1,082600	0.002162805	0.030005784
Ube2q2l	ENSRNOG00000023261	-1,082419	0.028859445	0.162791512
Trem2	ENSRNOG00000013578	-1,082005	0.048760427	0.214904513
Hbegf	ENSRNOG00000018646	-1,080580	0.014212705	0.106568425
Gdf11	ENSRNOG00000007610	-1,079406	0.000905929	0.016075601
Epb41l2	ENSRNOG00000012346	-1,077781	0.000397148	0.00862981
Mxra7	ENSRNOG00000042915	-1,077641	0.000152772	0.004102376
Tbc1d8b	ENSRNOG00000054011	-1,076963	3.31E-06	0.000180157
Cyp7b1	ENSRNOG00000009730	-1,076304	0.002697387	0.035226559
Nrp2	ENSRNOG00000031232	-1,073992	0.000161757	0.004264247
Plvap	ENSRNOG00000017676	-1,073185	0.004184725	0.04793972
Ctps2	ENSRNOG00000004257	-1,072008	4.64E-05	0.001601095
Dtx4	ENSRNOG00000021086	-1,071458	6.04E-07	4.18E-05
Tpm4	ENSRNOG00000015496	-1,070197	3.56E-07	2.66E-05
Cfap47	ENSRNOG00000030048	-1,070037	0.000444835	0.009429286
Laptm5	ENSRNOG00000011054	-1,069943	0.001023018	0.017611739
Gk	ENSRNOG00000034116	-1,069257	0.000526547	0.010723656
Arpc5	ENSRNOG00000028062	-1,068375	1.66E-05	0.00067711
Spidr	ENSRNOG00000037851	-1,066027	0.004720035	0.051823924
Morc4	ENSRNOG00000060846	-1,065381	2.94E-05	0.001099

Zfp52	ENSRNOG00000042826	-1,065048	0.006633624	0.064905582
Ccdc18	ENSRNOG00000024545	-1,063451	0.008039493	0.073896934
Lyn	ENSRNOG00000008180	-1,061884	0.000342259	0.007650488
Adamts4	ENSRNOG00000003538	-1,061538	0.023883232	0.145252898
Stk17b	ENSRNOG00000012502	-1,061372	0.001441489	0.022723366
Ugdh	ENSRNOG00000002643	-1,061108	7.05E-05	0.002196044
Septin10	ENSRNOG00000049507	-1,059388	0.000194404	0.004924893
Itgb3bp	ENSRNOG00000009116	-1,059240	0.017518824	0.120027829
Sptan1	ENSRNOG00000015396	-1,058851	0.000432337	0.009239789
Ell2	ENSRNOG00000027089	-1,058674	2.16E-05	0.000846475
Cd37	ENSRNOG00000020699	-1,058044	0.017150936	0.118709323
Btg2	ENSRNOG00000003300	-1,057100	0.002396711	0.032162575
Has2	ENSRNOG00000004854	-1,057040	0.025283598	0.150064176
Ccn2	ENSRNOG00000015036	-1,055517	0.007363787	0.069405811
Ppp1r12a	ENSRNOG00000004925	-1,054787	0.003417005	0.041689072
Enox1	ENSRNOG00000047817	-1,053055	0.034693752	0.180296417
Ly96	ENSRNOG00000067966	-1,052692	0.0409636	0.196918509
Olfml2b	ENSRNOG00000003018	-1,049794	2.64E-08	2.70E-06
Rn45s	ENSRNOG00000065988	-1,049773	0.035116449	0.181018762
Zfp40	ENSRNOG00000011675	-1,049317	0.002013559	0.028780958
Myo9b	ENSRNOG00000016256	-1,048343	0.001426917	0.022569909
Plac8	ENSRNOG00000002217	-1,047707	0.005243989	0.05546595
Nid1	ENSRNOG00000002461	-1,047612	0.000101954	0.002987896
Igfbp6	ENSRNOG00000010977	-1,046996	0.001290857	0.021037194
Nid2	ENSRNOG00000000341	-1,045770	6.19E-05	0.001988424
Renbp	ENSRNOG00000054765	-1,044938	9.24E-05	0.002746939
Raph1	ENSRNOG00000014722	-1,043716	7.19E-05	0.002235237
Pik3c2a	ENSRNOG00000020479	-1,042829	0.000521107	0.010626764
Atp11c	ENSRNOG00000003472	-1,042126	2.75E-06	0.000156966
Atp10d	ENSRNOG00000002312	-1,040923	0.000395366	0.008613798
Cysltr1	ENSRNOG00000037845	-1,039710	0.002576909	0.033937655
Dpysl3	ENSRNOG00000018992	-1,037856	7.40E-05	0.002284485
Rcn1	ENSRNOG00000013452	-1,037009	0.000278078	0.006488621
Fcgrt	ENSRNOG00000020583	-1,036841	7.56E-06	0.000361134
Chp2	ENSRNOG00000019112	-1,036255	0.004576512	0.050712704
Fyn	ENSRNOG00000000596	-1,035642	8.00E-05	0.002439847
Zfp36l2	ENSRNOG00000005067	-1,035203	1.57E-08	1.74E-06
Grn	ENSRNOG00000021031	-1,034439	2.24E-06	0.000133096
Arfip2	ENSRNOG00000018440	-1,033763	0.002061586	0.029055663
Fras1	ENSRNOG00000002053	-1,033412	0.002129289	0.02961993
Ccdc138	ENSRNOG00000028581	-1,030569	0.03785082	0.188648678
Zfp136	ENSRNOG00000016417	-1,026880	0.001483372	0.023180473
Tppp2	ENSRNOG00000025473	-1,026643	0.023395854	0.143676548
Ugcg	ENSRNOG00000015644	-1,026526	2.77E-06	0.000156966
Pls3	ENSRNOG00000027520	-1,026362	5.57E-05	0.0018235
Gbp7	ENSRNOG00000029191	-1,025163	0.004380935	0.049281567
Rock1	ENSRNOG00000031092	-1,024969	0.001045125	0.017893467
G6pd	ENSRNOG00000056728	-1,023983	0.000107641	0.003094182
Adgrl4	ENSRNOG00000033940	-1,022795	0.000341888	0.007650488
Frmd4b	ENSRNOG00000007764	-1,022692	0.010729214	0.08830489

Tbxas1	ENSRNOG00000007918	-1,022203	0.029690166	0.165553927
Dgkh	ENSRNOG00000010065	-1,021556	0.000881369	0.015783602
Olfml1	ENSRNOG00000056562	-1,019902	0.001473988	0.023103361
Serpinh1	ENSRNOG00000016831	-1,017233	0.000283481	0.006566671
Rhoj	ENSRNOG00000021919	-1,016627	4.04E-08	3.96E-06
Mgst2	ENSRNOG00000061857	-1,015796	0.023366946	0.143651291
Nav1	ENSRNOG00000008425	-1,015091	0.000222115	0.005373534
Taok3	ENSRNOG00000001138	-1,014583	0.031872401	0.171646046
Mmp14	ENSRNOG00000010947	-1,014148	4.05E-05	0.00144938
Ssc5d	ENSRNOG00000016687	-1,013418	0.000389394	0.008508781
Mfap5	ENSRNOG00000015505	-1,013206	0.000625131	0.012174428
Gfpt2	ENSRNOG00000002810	-1,013191	0.021400428	0.135882992
Tlr13	ENSRNOG00000061403	-1,012156	0.006602924	0.064875554
Tmed3	ENSRNOG00000013889	-1,011163	0.006613268	0.064883321
Adamts20	ENSRNOG00000033397	-1,010916	0.003464551	0.04210212
Reps2	ENSRNOG00000061508	-1,009704	0.004131285	0.047537233
Ninj1	ENSRNOG00000016587	-1,009572	0.00037128	0.008201204
Gcnt1	ENSRNOG00000062686	-1,008840	0.001138866	0.019058568
Actn1	ENSRNOG00000056756	-1,006177	0.002755717	0.03577839
Arrdc3	ENSRNOG00000045649	-1,005309	0.000384328	0.008409868
Col16a1	ENSRNOG00000031475	-1,003846	0.008274681	0.075171738
Tmsb4x	ENSRNOG00000047931	-1,003663	0.001851093	0.027116221
Npr1	ENSRNOG00000014684	-1,002404	0.002177597	0.030146336
Erbb2	ENSRNOG00000006450	-1,002015	0.006630372	0.064905582
Lima1	ENSRNOG00000059801	-1,001641	1.57E-05	0.000652692
Ucp2	ENSRNOG00000017854	-1,001530	7.38E-05	0.002284485
Plcb4	ENSRNOG00000033119	-1,001281	0.000849931	0.015326296
Ywhaz	ENSRNOG00000008195	-1,000022	2.18E-05	0.000848872
Ackr1	ENSRNOG00000069330	-0,998311	0.007722324	0.071872048
Thbd	ENSRNOG00000004687	-0,997868	0.000664473	0.0127651
Serpinf1	ENSRNOG00000003172	-0,997324	0.0007311	0.013756685
Abca8	ENSRNOG00000004040	-0,995262	1.91E-08	2.04E-06
Irf8	ENSRNOG00000017869	-0,993517	0.016251612	0.115464338
Casp4	ENSRNOG00000033697	-0,991581	0.007413958	0.069667949
Snora81	ENSRNOG00000060539	-0,991456	0.035134835	0.181018762
Arrdc1	ENSRNOG00000007622	-0,990663	0.002987168	0.037844417
Arhgap9	ENSRNOG00000006946	-0,990548	0.03719972	0.186746151
Clic1	ENSRNOG00000029682	-0,989990	6.76E-06	0.00033337
Gnrh1	ENSRNOG00000066847	-0,989952	0.002339122	0.031662486
Adcy7	ENSRNOG00000014776	-0,988041	0.005856236	0.05997984
Cspg4	ENSRNOG00000017208	-0,987910	0.000236886	0.005669255
Adgre1	ENSRNOG00000046254	-0,987872	0.002243654	0.030744173
Atl3	ENSRNOG00000021203	-0,987058	2.07E-07	1.68E-05
Abca8a	ENSRNOG00000004147	-0,986384	0.000130148	0.003644652
Abcg3l2	ENSRNOG00000064381	-0,985406	0.011866451	0.094374326
Plat	ENSRNOG00000019018	-0,985232	0.000871107	0.015617769
F8	ENSRNOG00000031197	-0,984744	0.001058639	0.018025794
Fkbp7	ENSRNOG00000011758	-0,984647	0.004873169	0.052916132
Cplane1	ENSRNOG00000042118	-0,983733	0.000448712	0.009498542
Dock2	ENSRNOG00000006932	-0,983414	0.029200394	0.164002214

Fabp5	ENSRNOG00000049075	-0,983403	0.022400243	0.139830986
Mapk8ip3	ENSRNOG00000033568	-0,982528	0.007462076	0.069993466
Rtl5	ENSRNOG00000045779	-0,982234	0.001334175	0.021562698
Igfbp4	ENSRNOG00000010635	-0,981830	3.11E-05	0.001149307
Angptl1	ENSRNOG00000004712	-0,981599	0.016692288	0.116831018
Ifi30	ENSRNOG00000019387	-0,980734	8.63E-05	0.002601642
Aox1	ENSRNOG00000015354	-0,980565	0.000466863	0.009789668
Tcirg1	ENSRNOG00000017220	-0,980278	0.008310964	0.075238131
Pnisr	ENSRNOG00000008782	-0,978689	0.013513135	0.103067274
Arhgap18	ENSRNOG00000011245	-0,977312	0.021130034	0.135023861
Txndc9	ENSRNOG00000018593	-0,977021	0.023357377	0.143651291
Plekhg3	ENSRNOG00000006570	-0,976283	0.002604534	0.034272498
Plcg1	ENSRNOG00000051490	-0,974154	0.002512198	0.03331068
Egfr	ENSRNOG00000004332	-0,974098	1.28E-05	0.000554487
Sec24d	ENSRNOG00000014872	-0,973399	6.22E-05	0.001994407
Dipk2a	ENSRNOG00000008519	-0,972434	0.000765327	0.014161273
Usp46	ENSRNOG00000002106	-0,971683	2.99E-05	0.001112453
Ankrd44	ENSRNOG00000021384	-0,971405	0.001107368	0.018635583
Tmem47	ENSRNOG00000030418	-0,969911	0.010927524	0.089417449
Mmp19	ENSRNOG00000006778	-0,969888	0.012669913	0.09877639
Ctsh	ENSRNOG00000014064	-0,968943	0.000708472	0.013428208
Fam171a2	ENSRNOG00000021041	-0,968943	0.013173745	0.101456721
Il6r	ENSRNOG00000020811	-0,968537	0.001365301	0.021794455
Pde4b	ENSRNOG00000005905	-0,967631	0.036281794	0.183951975
Ogfrl1	ENSRNOG00000014142	-0,966596	0.000300499	0.006895102
Carmil1	ENSRNOG00000016576	-0,965894	0.004161633	0.047780581
Ptprk	ENSRNOG00000047605	-0,964950	0.001395924	0.022169724
Kif13b	ENSRNOG00000013089	-0,964033	0.001108805	0.018635583
Cdkl2	ENSRNOG00000002506	-0,963393	0.034806401	0.180520548
Nf1	ENSRNOG00000013780	-0,963150	0.00014383	0.003938271
Fstl1	ENSRNOG00000002746	-0,962729	0.000161208	0.004256979
Myef2	ENSRNOG00000005258	-0,962642	0.000970068	0.016962583
Cd14	ENSRNOG00000017819	-0,962399	0.015824259	0.113928817
Clcn5	ENSRNOG00000002862	-0,962110	4.94E-06	0.000254143
Zdbf2	ENSRNOG00000024114	-0,961478	0.016620618	0.116538806
Prpf4b	ENSRNOG00000016705	-0,961448	0.002745788	0.035679207
Arhgef25	ENSRNOG00000005034	-0,960359	0.000278203	0.006488621
Nxf1	ENSRNOG00000019069	-0,959947	0.00376394	0.04456093
Dock8	ENSRNOG00000015894	-0,959454	0.009741885	0.08336163
Tnfrsf26	ENSRNOG00000043486	-0,958370	0.000558117	0.011219954
Dysf	ENSRNOG00000032788	-0,958207	1.09E-05	0.000487285
Shank3	ENSRNOG00000052296	-0,957493	0.004017605	0.046650646
Uap1	ENSRNOG00000002926	-0,957273	0.006214541	0.062466165
Parp14	ENSRNOG00000023334	-0,956380	0.001659125	0.025218704
Tgif1	ENSRNOG00000015906	-0,956072	0.03506058	0.181018762
Cdk12	ENSRNOG00000006000	-0,956018	0.003265795	0.040349796
Cst7	ENSRNOG00000006767	-0,955747	0.027314063	0.157482075
Tm6sf1	ENSRNOG00000019662	-0,954529	0.000854601	0.015392706
Ccn1	ENSRNOG00000014350	-0,954318	0.017245394	0.118939018
Slc28a2	ENSRNOG00000028668	-0,954177	0.004988524	0.053823551

Slfn5	ENSRNOG00000021719	-0,953801	0.000484715	0.010025837
Nin	ENSRNOG00000005540	-0,952599	0.000818958	0.01484226
Pabpn1	ENSRNOG00000042195	-0,952152	0.000140604	0.003864023
Slc40a1	ENSRNOG00000003872	-0,951180	0.028499459	0.161403697
Itga7	ENSRNOG00000007905	-0,950896	0.000155668	0.00414583
Dynll1	ENSRNOG00000011222	-0,950339	0.035949808	0.18288132
Cd38	ENSRNOG00000003069	-0,950246	5.41E-05	0.001791724
Mdfic	ENSRNOG00000053787	-0,949931	0.000604133	0.011929527
Rasa2	ENSRNOG00000011909	-0,949907	0.00296071	0.037624685
Edem1	ENSRNOG00000055365	-0,949289	2.76E-09	3.81E-07
Dsel	ENSRNOG00000032307	-0,948966	0.003472757	0.042138285
Plpp1	ENSRNOG00000009980	-0,947000	0.002335028	0.031634551
Mfap3l	ENSRNOG00000011775	-0,946208	0.010264546	0.085748862
Zfp692	ENSRNOG00000002682	-0,945596	0.010721951	0.088291753
Pla2g4a	ENSRNOG00000002657	-0,944251	0.008086337	0.074136157
Zfp709l1	ENSRNOG00000066372	-0,944236	0.005828371	0.059780128
Lamb1	ENSRNOG00000005678	-0,943078	0.000129244	0.003634694
Svep1	ENSRNOG00000033110	-0,937558	0.000988836	0.017194268
Klf3	ENSRNOG00000002163	-0,936645	1.50E-07	1.27E-05
Pkp4	ENSRNOG00000005504	-0,936455	1.42E-06	8.91E-05
Hacd4	ENSRNOG00000005772	-0,935805	0.009817245	0.083717941
Lmo7	ENSRNOG00000060775	-0,935712	0.000549478	0.011074855
Ccna2	ENSRNOG00000015423	-0,935275	0.021565818	0.136195962
Golgb1	ENSRNOG00000030314	-0,935248	0.000501491	0.010313746
Cblb	ENSRNOG00000001982	-0,934730	0.002609206	0.034305004
Uap1l1	ENSRNOG00000012960	-0,934176	0.002500171	0.033207731
Slc44a2	ENSRNOG00000031824	-0,933766	0.000196477	0.004937273
Pik3r1	ENSRNOG00000018903	-0,932875	0.007762608	0.072172727
Mertk	ENSRNOG00000017319	-0,932068	0.014645235	0.108422211
Ifitm10	ENSRNOG00000020164	-0,932043	0.000252948	0.005998371
Knl1	ENSRNOG00000060100	-0,931184	0.01575255	0.113732773
Tmbim1	ENSRNOG00000014797	-0,930777	0.000219795	0.005342284
Adam33	ENSRNOG00000021242	-0,928573	0.005071636	0.054382245
Tasor	ENSRNOG00000014750	-0,928399	6.32E-05	0.0020199
Gbp2	ENSRNOG00000031743	-0,928027	0.005170194	0.055059209
Ppp1r18	ENSRNOG00000000818	-0,927223	0.001903309	0.027739522
Map7d3	ENSRNOG00000027831	-0,926208	0.035100714	0.181018762
Rasgrp2	ENSRNOG00000021098	-0,924677	0.017216094	0.118842152
Hdgfl3	ENSRNOG00000019740	-0,922725	0.000149675	0.004048496
Steap4	ENSRNOG00000008602	-0,922673	0.000113831	0.003236277
Lrch1	ENSRNOG00000009412	-0,922520	0.002038187	0.028913108
Npas2	ENSRNOG00000013408	-0,921487	0.003794201	0.044789263
Actg1	ENSRNOG00000036701	-0,921186	0.006441485	0.063865082
Scai	ENSRNOG00000025278	-0,920491	0.000992601	0.017221289
Tram2	ENSRNOG00000013046	-0,920027	0.008732355	0.077654165
Pnn	ENSRNOG00000004061	-0,918004	0.010438953	0.086833365
Slc8b1	ENSRNOG00000001383	-0,917701	0.008620238	0.076964648
Ttc21b	ENSRNOG00000005868	-0,917676	0.011032921	0.089839098
Abcg3l1	ENSRNOG00000069481	-0,917639	0.000686109	0.013098612
Rtl1	ENSRNOG00000048751	-0,917554	0.011203412	0.090816421

Mis18bp1	ENSRNOG00000023093	-0,916661	0.041186875	0.197343617
Ppp1r12c	ENSRNOG00000053828	-0,916344	0.003551272	0.042791047
Sparc	ENSRNOG00000012840	-0,914653	0.000983351	0.017137141
Ndrg1	ENSRNOG00000007393	-0,914619	6.82E-05	0.00214578
Clic4	ENSRNOG00000018109	-0,914289	0.00423954	0.048248381
Zim1	ENSRNOG00000015071	-0,913640	0.004218812	0.048117933
Maged2	ENSRNOG00000002449	-0,912503	0.003316827	0.040818456
Adamtsl1	ENSRNOG00000006956	-0,912255	0.000188608	0.004825156
Pgm3	ENSRNOG00000009515	-0,912111	0.002292428	0.031138648
Syne2	ENSRNOG00000005323	-0,911994	0.00053745	0.010874633
Zcchc7	ENSRNOG00000013078	-0,911769	0.017368151	0.119405545
Pld2	ENSRNOG00000019604	-0,911390	0.006210206	0.062466165
Atad2	ENSRNOG00000025604	-0,910717	0.003951642	0.04615186
Map3k8	ENSRNOG00000016378	-0,910058	0.012257069	0.096447035
Tmem176b	ENSRNOG00000008465	-0,908938	6.96E-06	0.000338982
Slc44a1	ENSRNOG00000055089	-0,907083	0.000111591	0.003185231
Hltf	ENSRNOG00000000082	-0,906777	5.00E-05	0.001689228
Isyna1	ENSRNOG00000019741	-0,906620	0.019095757	0.127632729
Cenpc	ENSRNOG00000021776	-0,906135	0.002506711	0.033266228
Rnd3	ENSRNOG00000004624	-0,905859	0.001084909	0.018372697
Tuba1a	ENSRNOG00000053468	-0,905427	0.000585264	0.011645488
Gdi1	ENSRNOG00000056870	-0,904909	0.006813925	0.066061576
Notch2	ENSRNOG00000018835	-0,903909	2.76E-09	3.81E-07
Dusp6	ENSRNOG00000023896	-0,903870	0.006037823	0.061203176
Haus6	ENSRNOG00000046028	-0,903300	0.003630093	0.04337182
Plin2	ENSRNOG00000007060	-0,903180	4.26E-05	0.00149867
Sptlc2	ENSRNOG00000012210	-0,903012	0.000268339	0.006305769
Enah	ENSRNOG00000031934	-0,902728	0.010083326	0.084947786
Nras	ENSRNOG00000023079	-0,901174	2.81E-05	0.001053428
Nktr	ENSRNOG00000049128	-0,901058	0.010530597	0.087362465
Cds2	ENSRNOG00000021265	-0,900444	0.003674216	0.043764739
Fnbp1	ENSRNOG00000008258	-0,900184	0.000312787	0.007124595
Zfp9	ENSRNOG00000006726	-0,898261	0.042744564	0.20101428
Fst	ENSRNOG00000011631	-0,897799	0.017840894	0.121752573
Kcnn3	ENSRNOG00000020706	-0,897260	0.003728956	0.044268376
Grk5	ENSRNOG00000011439	-0,896367	5.65E-05	0.001844264
Snx7	ENSRNOG00000017077	-0,896169	0.00068304	0.013057378
Niban2	ENSRNOG00000015845	-0,895725	8.09E-05	0.002462383
Ddr2	ENSRNOG00000002881	-0,895469	2.64E-05	0.000994691
Lclat1	ENSRNOG00000034026	-0,894756	0.000659946	0.01269377
Gabbr1	ENSRNOG00000000774	-0,894458	0.000420673	0.009015242
Epha7	ENSRNOG00000007030	-0,894397	0.000694097	0.013203946
Zcchc9	ENSRNOG00000051682	-0,892601	0.014205653	0.106568425
Myf6	ENSRNOG00000004878	-0,892338	0.001350717	0.021672562
Prcp	ENSRNOG00000010630	-0,891875	0.001973341	0.0283204
Mvp	ENSRNOG00000020182	-0,891728	3.01E-05	0.001116676
Srrt	ENSRNOG00000048277	-0,890118	0.01180606	0.094134295
Pip4k2a	ENSRNOG00000016670	-0,887881	0.004290811	0.048654169
Zc3h7a	ENSRNOG00000002420	-0,887551	0.000952826	0.016717371
Smarcd2	ENSRNOG00000010557	-0,887393	9.36E-06	0.000430264

Cep95	ENSRNOG00000014354	-0,887267	0.004004975	0.046630663
Cd151	ENSRNOG00000062573	-0,886976	5.37E-07	3.78E-05
Jmjd1c	ENSRNOG00000000648	-0,886084	0.008427548	0.075896644
Cmtm6	ENSRNOG00000010951	-0,885956	0.001387624	0.022060396
Sulf1	ENSRNOG00000009037	-0,884910	0.001837579	0.026983494
Nlrp3	ENSRNOG00000003170	-0,884700	0.047066146	0.210681663
Helb	ENSRNOG00000004189	-0,884670	0.005481931	0.057282684
Inf2	ENSRNOG00000028650	-0,884073	0.015517102	0.11291754
Fgd5	ENSRNOG00000010213	-0,883978	0.001016788	0.017542777
Btg3	ENSRNOG00000001555	-0,883713	0.005924092	0.060443582
Pdlim2	ENSRNOG00000008543	-0,883535	0.018127057	0.12305863
Cnn3	ENSRNOG00000011559	-0,882943	0.000136049	0.003764906
Col4a5	ENSRNOG00000018951	-0,882800	0.00048206	0.010002647
Unc93b1	ENSRNOG00000017703	-0,882664	0.0092301	0.080542711
Ebf1	ENSRNOG00000028845	-0,880877	0.00217874	0.030146336
Cd2ap	ENSRNOG00000011987	-0,879730	0.001040501	0.017833888
Omd	ENSRNOG00000039560	-0,878008	0.038549198	0.190605048
Tifa	ENSRNOG00000010941	-0,877506	0.003603152	0.043116056
Dync2h1	ENSRNOG00000032070	-0,877277	0.000196362	0.004937273
Myd88	ENSRNOG00000013634	-0,877182	0.021191842	0.135099801
C1galt1	ENSRNOG00000007804	-0,876305	0.00585465	0.05997984
Efhd2	ENSRNOG00000013783	-0,875682	0.000549222	0.011074855
Atm	ENSRNOG00000029773	-0,874449	0.001642323	0.025036585
Armcx4	ENSRNOG00000043128	-0,874357	0.000112235	0.003196748
Ptprj	ENSRNOG00000034025	-0,873964	0.000109101	0.003130378
Cyp39a1	ENSRNOG00000010519	-0,873241	0.006673945	0.06516986
Dipk2b	ENSRNOG00000004265	-0,872091	0.014472064	0.107728025
Trim25	ENSRNOG00000002341	-0,871638	0.004316561	0.048875019
Pld1	ENSRNOG00000028156	-0,870519	0.0102819	0.085801821
Ly6e	ENSRNOG00000007091	-0,870001	0.004010163	0.046630663
Tcp11l1	ENSRNOG00000042576	-0,869698	7.03E-07	4.78E-05
Zmym5	ENSRNOG00000029220	-0,869243	0.008054857	0.073994495
Cep170	ENSRNOG00000004127	-0,868641	0.003490627	0.042223582
Sirpa	ENSRNOG00000004763	-0,867895	0.000783976	0.014403706
Hectd2	ENSRNOG00000056753	-0,867775	0.001621616	0.024891401
Islr	ENSRNOG00000048924	-0,866335	0.014218831	0.106568425
Slc25a5	ENSRNOG00000039980	-0,865693	0.003017467	0.03812825
Otulin	ENSRNOG00000012017	-0,863842	0.001017886	0.017542777
Tmem164	ENSRNOG00000012787	-0,862605	0.00215392	0.029909157
Prrx1	ENSRNOG00000003720	-0,861980	0.000496698	0.010249742
Chrdl1	ENSRNOG00000004330	-0,861202	0.001370209	0.021850412
Abracl	ENSRNOG00000051450	-0,860830	0.016530034	0.116369757
Lama4	ENSRNOG00000000599	-0,860707	0.0005299	0.010752187
Golm1	ENSRNOG00000018400	-0,859012	0.004094015	0.047245143
Pabpc1	ENSRNOG00000008639	-0,858920	0.000210632	0.005200696
Trim47	ENSRNOG00000008215	-0,858501	0.000279326	0.006488621
Abr	ENSRNOG00000056837	-0,858244	2.92E-06	0.000163257
Thoc1	ENSRNOG00000015332	-0,857135	0.00891226	0.078938089
Zfp275	ENSRNOG00000061073	-0,856554	0.003602272	0.043116056
Snora53	ENSRNOG00000056599	-0,856318	0.027063262	0.156687334

Coq10b	ENSRNOG00000014456	-0,856174	0.023459404	0.143953334
Septin8	ENSRNOG00000007462	-0,856164	5.86E-05	0.001901134
Aplf	ENSRNOG00000043059	-0,855699	0.004840362	0.052662593
Eml4	ENSRNOG00000030294	-0,855319	0.000577713	0.011524664
Parva	ENSRNOG00000015713	-0,854618	0.000197596	0.004957394
Tusc3	ENSRNOG00000013061	-0,854507	0.010508833	0.087228352
Stat3	ENSRNOG00000019742	-0,854257	0.001349362	0.021672562
Anxa7	ENSRNOG00000007136	-0,854110	0.000236449	0.005669255
Lfng	ENSRNOG00000001250	-0,854032	0.001721032	0.025836179
Atrnl1	ENSRNOG00000017406	-0,852849	0.000349429	0.007766196
Crip1	ENSRNOG00000027990	-0,851948	0.000299165	0.00687462
Paxbp1	ENSRNOG00000002036	-0,851426	0.010608951	0.087912392
Washc4	ENSRNOG00000008331	-0,851010	0.000131483	0.003668087
Usp54	ENSRNOG00000027012	-0,850832	0.009571615	0.082435464
Clec3b	ENSRNOG00000004540	-0,850787	0.008364896	0.075550775
Col4a1	ENSRNOG00000016281	-0,850322	0.000183535	0.004703076
Trafd1	ENSRNOG00000001351	-0,849936	0.001217056	0.020150618
Trim37	ENSRNOG00000006248	-0,849902	5.31E-05	0.001774227
Kif3c	ENSRNOG00000011394	-0,849314	0.00022886	0.005502525
Nab2	ENSRNOG00000008415	-0,849105	0.018840096	0.126194621
Mtmr12	ENSRNOG00000022929	-0,849031	0.00012416	0.003498045
Zswim6	ENSRNOG00000014528	-0,847818	0.021547579	0.136135959
Skap2	ENSRNOG00000012228	-0,846556	0.016952293	0.118042402
Nherf2	ENSRNOG00000002997	-0,846289	0.002030527	0.028886818
Nfil3	ENSRNOG00000011668	-0,845643	0.018097163	0.122909243
Actb	ENSRNOG00000034254	-0,843880	0.001057662	0.018025794
Cep128	ENSRNOG00000003908	-0,843448	0.013591207	0.103417027
Mysm1	ENSRNOG00000026299	-0,843105	0.00561622	0.058178666
Cntrl	ENSRNOG00000022015	-0,842260	0.022205334	0.138947902
Frmd6	ENSRNOG00000007329	-0,841882	9.37E-05	0.00277493
Ifitm2	ENSRNOG00000014936	-0,841270	4.85E-05	0.001647043
Camk2d	ENSRNOG00000011589	-0,840644	0.000720434	0.013603908
Fabp4	ENSRNOG00000010805	-0,840457	2.08E-06	0.000124582
Coro1a	ENSRNOG00000019430	-0,840320	0.015714697	0.113567565
Pde5a	ENSRNOG00000014443	-0,840122	0.006161711	0.062296855
Sptbn1	ENSRNOG00000005434	-0,839671	0.001271324	0.020784083
Arhgdib	ENSRNOG00000005809	-0,838349	0.002953734	0.037597361
Lpar6	ENSRNOG00000015577	-0,837649	0.023291785	0.143546679
Scn3a	ENSRNOG00000005007	-0,837275	0.003081598	0.038665036
Mob3b	ENSRNOG00000009453	-0,837269	0.018668882	0.125414757
Katnbl1	ENSRNOG00000005814	-0,837015	0.002923863	0.037278057
Dpy19l1	ENSRNOG00000026589	-0,836353	0.000179754	0.004629026
Sash1	ENSRNOG00000013160	-0,835193	2.83E-09	3.86E-07
Ppm1h	ENSRNOG00000004314	-0,834704	0.02526711	0.150049714
Prex2	ENSRNOG00000005391	-0,834314	0.008777427	0.077965967
Csf1	ENSRNOG00000018659	-0,833578	0.000707474	0.013425632
Snx14	ENSRNOG00000011348	-0,831948	0.005976473	0.060898262
Ptgfrn	ENSRNOG00000015655	-0,830867	0.002844798	0.036509022
Plp1	ENSRNOG00000002419	-0,830393	0.027636935	0.158769707
Traf3ip2	ENSRNOG00000000595	-0,830136	0.008766379	0.077912256

Ctsl	ENSRNOG00000018566	-0,829732	2.32E-07	1.85E-05
Myadm	ENSRNOG00000053450	-0,829727	0.000265724	0.006255309
Acsl5	ENSRNOG00000016265	-0,829019	0.005591199	0.057992791
Lgals3bp	ENSRNOG00000003217	-0,828249	0.004404785	0.04944276
Grb10	ENSRNOG00000004290	-0,828101	0.002390878	0.032111965
Matn2	ENSRNOG00000006060	-0,827607	0.044401657	0.204490707
Dap	ENSRNOG00000010747	-0,827324	0.000697097	0.013244836
Rab3il1	ENSRNOG00000020349	-0,827260	0.009423057	0.081663221
Baz1a	ENSRNOG00000006828	-0,826074	0.006014855	0.061089595
Gdap1	ENSRNOG00000005850	-0,825674	0.0470514	0.210681663
Mrc2	ENSRNOG00000006548	-0,825194	0.002798096	0.036117923
Carhsp1	ENSRNOG00000002610	-0,825107	0.002820259	0.036283763
Fuca1	ENSRNOG00000009325	-0,824634	0.002416817	0.032376615
Flna	ENSRNOG00000054890	-0,824105	0.006460341	0.063987361
Mycbp2	ENSRNOG00000010479	-0,823953	0.004746937	0.051972787
Synj1	ENSRNOG00000002051	-0,823425	0.001406468	0.022314426
Tll1	ENSRNOG00000033528	-0,822870	0.002120013	0.029517256
Taf1	ENSRNOG00000002565	-0,821863	0.012631022	0.098592849
Ap3s1	ENSRNOG00000003829	-0,820738	0.002696232	0.035226559
Timp2	ENSRNOG00000033143	-0,819968	8.24E-06	0.000386822
Fndc3b	ENSRNOG00000024089	-0,819859	0.000259325	0.006140258
Spata5	ENSRNOG00000017462	-0,819600	0.006335199	0.063347559
Cytip	ENSRNOG00000004772	-0,818480	0.04528326	0.206049414
Tanc2	ENSRNOG00000052840	-0,818376	0.0047079	0.051730646
Icam1	ENSRNOG00000020679	-0,816900	0.044060213	0.204001135
Nfxl1	ENSRNOG00000058588	-0,816489	0.001944577	0.028130471
Cfl1	ENSRNOG00000020660	-0,816234	2.16E-05	0.000846475
Ston1	ENSRNOG00000016688	-0,815886	6.33E-05	0.0020199
Sh3d19	ENSRNOG00000011752	-0,815569	8.60E-06	0.000400939
Kitlg	ENSRNOG00000005386	-0,814855	0.005987668	0.060932641
Stard9	ENSRNOG00000010050	-0,814766	0.007289849	0.068959229
Hrct1	ENSRNOG00000039336	-0,814761	0.015697442	0.113567565
Prkra	ENSRNOG00000011195	-0,814587	0.000419428	0.009012064
Ophn1	ENSRNOG00000026573	-0,814343	0.009173808	0.080296589
Htra3	ENSRNOG00000008182	-0,814280	0.000192757	0.0048991
Rasa1	ENSRNOG00000029185	-0,813881	2.78E-05	0.001042569
Sytl4	ENSRNOG00000003526	-0,812989	0.002132225	0.029634314
Fcna	ENSRNOG00000017063	-0,812868	0.022019711	0.138307617
Efhc1	ENSRNOG00000042729	-0,812690	0.027884101	0.159601136
Myh9	ENSRNOG00000049236	-0,812677	0.001645664	0.025062993
Fkbp5	ENSRNOG00000022523	-0,812263	0.024021391	0.145567199
Fmo3	ENSRNOG00000003620	-0,812187	0.048900992	0.215158842
Cygb	ENSRNOG00000011541	-0,810403	4.46E-06	0.000233003
Mfhas1	ENSRNOG00000011431	-0,809310	0.028805872	0.162702578
Metrnl	ENSRNOG00000046202	-0,808187	0.019179569	0.12797331
Fubp1	ENSRNOG00000043370	-0,807836	0.01070404	0.088284246
Arrb1	ENSRNOG00000030404	-0,807235	0.032545311	0.174126353
Eps8	ENSRNOG00000007047	-0,805045	0.003932481	0.046031593
Slc35d2	ENSRNOG00000027229	-0,804811	0.022700884	0.141000744
Arfgap3	ENSRNOG00000046472	-0,804638	0.000371633	0.008201204

Tmem237	ENSRNOG00000024085	-0,804500	0.00092272	0.016244047
Serpinb6a	ENSRNOG00000017962	-0,804239	0.001776079	0.026378757
Morc3	ENSRNOG00000026236	-0,804106	0.010355751	0.086279469
Flrt2	ENSRNOG00000003732	-0,803947	0.000226366	0.005450972
Tp63	ENSRNOG00000001924	-0,803922	0.001956194	0.028250285
Rasa3	ENSRNOG00000017671	-0,803257	0.000131609	0.003668087
Elf1	ENSRNOG00000011762	-0,802753	4.08E-05	0.001451112
Clec1a	ENSRNOG00000060460	-0,801374	0.041863881	0.198685187
Spats2	ENSRNOG00000052307	-0,800627	0.001244969	0.020449203
Acap2	ENSRNOG00000001730	-0,800307	5.37E-06	0.000272525
Ctif	ENSRNOG00000018342	-0,799076	0.0316303	0.170773272
Ptms	ENSRNOG00000060098	-0,797654	0.006537048	0.064419487
Arl6ip1	ENSRNOG00000017751	-0,797448	0.002864577	0.036702393
Tubb5	ENSRNOG00000061216	-0,797008	1.20E-05	0.000524242
Mcf2l	ENSRNOG00000028426	-0,796887	0.044804929	0.205070738
Zfp521	ENSRNOG00000016874	-0,795320	0.006512071	0.064295352
Tek	ENSRNOG00000008587	-0,794712	0.00227822	0.031026808
Sorbs2	ENSRNOG00000013391	-0,794180	8.56E-05	0.002585578
Ttc14	ENSRNOG00000011261	-0,794025	0.002551534	0.033652153
Rbp7	ENSRNOG00000015850	-0,793682	0.020216894	0.131845633
Zfp958	ENSRNOG00000065430	-0,793108	0.015221251	0.111703763
G2e3	ENSRNOG00000004232	-0,793047	0.002035581	0.028910072
Fgfr1	ENSRNOG00000016050	-0,789207	0.000745824	0.013944763
Swap70	ENSRNOG00000009910	-0,788872	0.000791701	0.01450608
Fnbp4	ENSRNOG00000007241	-0,788404	0.033243875	0.176176909
Col4a2	ENSRNOG00000023972	-0,788037	0.000982007	0.017132885
Il33	ENSRNOG00000016456	-0,787028	0.000111626	0.003185231
Capg	ENSRNOG00000013668	-0,785650	0.007110236	0.067961643
Copz2	ENSRNOG00000009225	-0,785559	0.002308774	0.031333364
Sf3b1	ENSRNOG00000013516	-0,785439	0.0049086	0.053182194
Rgs2	ENSRNOG00000003687	-0,784675	0.031984751	0.172132099
Tanc1	ENSRNOG00000025394	-0,783944	0.013563346	0.103282961
Inpp5d	ENSRNOG00000017020	-0,783777	0.039654977	0.1938332
Rbms1	ENSRNOG00000008482	-0,783700	0.000331193	0.007479318
Yes1	ENSRNOG00000037227	-0,783645	0.010672844	0.088073571
Plxna2	ENSRNOG00000007324	-0,783581	0.00643895	0.063865082
Plekhg5	ENSRNOG00000022694	-0,783501	0.038979718	0.191713916
Ddx10	ENSRNOG00000012500	-0,783115	0.007228331	0.068669145
Usf3	ENSRNOG00000027756	-0,782760	0.015462132	0.112728923
Dnaaf9	ENSRNOG00000021237	-0,782722	0.00162086	0.024891401
Kank3	ENSRNOG00000007230	-0,782679	0.020580183	0.133131501
Slc38a6	ENSRNOG00000022622	-0,782626	0.000710406	0.013448515
Col12a1	ENSRNOG00000058470	-0,781974	0.006636367	0.064905582
Pik3cg	ENSRNOG00000009385	-0,781234	0.005079067	0.054386162
Septin9	ENSRNOG00000002807	-0,781166	3.24E-06	0.000177604
Fmnl3	ENSRNOG00000056297	-0,780739	0.00273429	0.035589176
Fndc3a	ENSRNOG00000014478	-0,780607	0.000132935	0.00369184
Rnf213	ENSRNOG00000029658	-0,780310	0.00791159	0.073326932
Vmp1	ENSRNOG00000003967	-0,780117	0.004213682	0.048094628
Peak1	ENSRNOG00000042519	-0,779816	4.14E-05	0.001463091

Baz2b	ENSRNOG00000056984	-0,779733	0.018544599	0.125184076
Ppp1r9b	ENSRNOG00000052113	-0,779334	5.27E-06	0.000268512
Klc1	ENSRNOG00000011572	-0,779157	0.000221982	0.005373534
Myo5a	ENSRNOG00000058866	-0,778917	0.005379871	0.05647717
Cst3	ENSRNOG00000005195	-0,778118	0.001415575	0.022436077
Plekha4	ENSRNOG00000020942	-0,777164	0.026451488	0.15481374
Pola1	ENSRNOG00000013322	-0,776626	0.030984174	0.169031608
Wwc2	ENSRNOG00000013248	-0,776043	0.017387183	0.119405545
Spg21	ENSRNOG00000015019	-0,775786	0.00115837	0.019302032
Lgals1	ENSRNOG00000009884	-0,775583	0.013057823	0.100813119
Smarcad1	ENSRNOG00000006391	-0,775358	0.005317323	0.055900058
Ptn	ENSRNOG00000011946	-0,774801	0.003312451	0.040796826
Klhl11	ENSRNOG00000016921	-0,774497	0.009355245	0.081290979
Btnl9	ENSRNOG00000046377	-0,773960	0.004402362	0.04944276
Tpst1	ENSRNOG00000000900	-0,773139	0.014167617	0.106530636
Olfml3	ENSRNOG00000019211	-0,772174	0.023598109	0.14429299
Prelp	ENSRNOG00000003120	-0,772164	0.013413109	0.102662939
Cnot6	ENSRNOG00000054891	-0,772046	0.007624594	0.071132444
Ifngr1	ENSRNOG00000012074	-0,771749	0.000564078	0.011296066
Zc3hav1	ENSRNOG00000013948	-0,771737	0.010086868	0.084947786
Vipas39	ENSRNOG00000049223	-0,771609	0.002098598	0.029403023
Pfkp	ENSRNOG00000017163	-0,771343	0.007238964	0.068686393
Slc8a1	ENSRNOG00000008479	-0,771229	1.12E-07	9.80E-06
Tcn2	ENSRNOG00000004280	-0,771114	0.002987717	0.037844417
Elf4	ENSRNOG00000005352	-0,769536	0.001721306	0.025836179
Adar	ENSRNOG00000020744	-0,769528	0.011036716	0.089839098
Abi2	ENSRNOG00000017707	-0,769248	0.006385704	0.063612061
Ace	ENSRNOG00000062101	-0,768685	1.10E-06	7.16E-05
Gab2	ENSRNOG00000011882	-0,768525	0.001683559	0.025441167
Clk1	ENSRNOG00000025768	-0,768386	0.027110885	0.156904748
Dock9	ENSRNOG00000011969	-0,768068	0.010370649	0.086350877
Spry1	ENSRNOG00000025371	-0,767172	0.001277017	0.020833425
Mamdc2	ENSRNOG00000024620	-0,767171	0.0083284	0.075352191
Atp13a3	ENSRNOG00000066681	-0,766894	0.003732661	0.044268376
Iffo1	ENSRNOG00000018533	-0,766482	0.00177267	0.026363029
Elovl5	ENSRNOG00000006331	-0,764892	0.002718787	0.035416976
Glt8d2	ENSRNOG00000033579	-0,764880	0.030904091	0.168926504
Igf2r	ENSRNOG00000014997	-0,764719	0.001435867	0.022665452
Rasgrf2	ENSRNOG00000056151	-0,764589	0.018492461	0.12501656
Dennd2a	ENSRNOG00000026748	-0,763427	0.019318134	0.128567506
Bcl2l11	ENSRNOG00000016551	-0,763345	0.038134958	0.189451337
Usp37	ENSRNOG00000022720	-0,762013	0.00075678	0.014069968
Sh3bgrl3	ENSRNOG00000015967	-0,761622	0.01861402	0.125326894
Flnb	ENSRNOG00000009470	-0,761405	0.015998363	0.114441917
Fosl2	ENSRNOG00000068412	-0,761368	0.004288032	0.048654169
Ldb2	ENSRNOG00000003205	-0,761266	0.01256773	0.098147986
Slc39a14	ENSRNOG00000009832	-0,760798	0.005223221	0.055373135
Plcl1	ENSRNOG00000032659	-0,760477	0.004420525	0.049582777
Fer	ENSRNOG00000015898	-0,760050	0.015664422	0.113459644
Timp4	ENSRNOG00000007955	-0,759902	0.001647427	0.025065344

Ints2	ENSRNOG00000003576	-0,759714	0.006509726	0.064295352
Stag1	ENSRNOG00000015389	-0,759525	0.001316034	0.02135813
Dlgap4	ENSRNOG00000020225	-0,759449	2.18E-05	0.000848872
Atp6v1h	ENSRNOG00000030862	-0,758991	0.010206615	0.085494119
Arhgef15	ENSRNOG00000004566	-0,758255	0.018675368	0.125414757
Col15a1	ENSRNOG00000060381	-0,758174	0.004635276	0.051145613
Arap1	ENSRNOG00000019555	-0,758034	0.003153601	0.039243696
Zfp36l1	ENSRNOG00000058646	-0,756487	3.57E-05	0.001299452
B4galt5	ENSRNOG00000008283	-0,755810	0.012150009	0.09594381
Shc1	ENSRNOG00000020657	-0,754752	3.41E-05	0.001246033
Cd47	ENSRNOG00000001964	-0,754498	0.008562859	0.076584008
Zfand2a	ENSRNOG00000032917	-0,754482	4.45E-06	0.000233003
Rbm26	ENSRNOG00000009836	-0,753172	0.013805624	0.104362751
Tgfbr1	ENSRNOG00000007036	-0,752112	0.001575597	0.024352972
Ifrd1	ENSRNOG00000050997	-0,751949	0.000558931	0.011221833
Arl13b	ENSRNOG00000047194	-0,751247	0.01615779	0.115126716
Slc12a4	ENSRNOG00000019651	-0,751154	0.005107159	0.054624446
Wdr19	ENSRNOG00000002932	-0,750179	0.029857016	0.165823626
Chd1	ENSRNOG00000014434	-0,750117	0.006379984	0.063595747
Cul4b	ENSRNOG00000002585	-0,749735	0.000753607	0.014027712
Glipr2	ENSRNOG00000014838	-0,749349	0.012063204	0.095500362
Spire1	ENSRNOG00000025324	-0,749290	0.000209125	0.005171683
Cavin3	ENSRNOG00000017914	-0,748939	8.38E-05	0.002539155
Hnrnpa1	ENSRNOG00000036839	-0,748189	0.010070617	0.084947786
Irf3	ENSRNOG00000043388	-0,747978	0.033688265	0.176910609
Asah1	ENSRNOG00000010034	-0,746998	0.003437452	0.041872946
Myo9a	ENSRNOG00000011619	-0,746918	0.001820789	0.026863537
Zeb2	ENSRNOG00000004677	-0,746707	9.50E-05	0.002807892
Prkx	ENSRNOG00000060168	-0,746522	0.003244442	0.040149648
Pgghg	ENSRNOG00000014783	-0,745834	0.003078072	0.038665036
Panx1	ENSRNOG00000010060	-0,744173	0.045531003	0.206934954
Tmem117	ENSRNOG00000006068	-0,744055	0.003919412	0.045947656
Rsrp1	ENSRNOG00000017309	-0,743613	0.005078045	0.054386162
Zw10	ENSRNOG00000054479	-0,743512	0.000609051	0.011996232
Rcn3	ENSRNOG00000043007	-0,743370	0.008200163	0.074710083
Cttnbp2nl	ENSRNOG00000014479	-0,743262	0.000638705	0.01237689
Adgrg1	ENSRNOG00000014963	-0,742266	0.000563721	0.011296066
Gnai3	ENSRNOG00000019465	-0,742061	0.005224551	0.055373135
Dok2	ENSRNOG00000013922	-0,741202	0.028967407	0.163105242
Nek10	ENSRNOG00000005883	-0,741059	0.021480077	0.136025567
Frk	ENSRNOG00000000543	-0,740670	0.01916322	0.127939777
Arhgap42	ENSRNOG00000026821	-0,739448	0.001442452	0.022723366
Fcho2	ENSRNOG00000015334	-0,738955	0.005024498	0.054061932
Chodl	ENSRNOG00000001915	-0,738724	0.027690992	0.158904478
Psd3	ENSRNOG00000013884	-0,738505	0.043389667	0.202519774
Asap1	ENSRNOG00000058733	-0,737937	0.004096803	0.047245143
Ift140	ENSRNOG00000016545	-0,737809	0.042770306	0.2010487
Tacc1	ENSRNOG00000016423	-0,737673	0.000401246	0.008694585
Trmt2a	ENSRNOG00000001885	-0,737413	0.015462241	0.112728923
Rbm25	ENSRNOG00000002874	-0,737180	0.001792646	0.026548876

Zfp862	ENSRNOG00000066193	-0,737058	0.016863876	0.117504112
Pdgfd	ENSRNOG00000029148	-0,735983	0.0252061	0.149881211
Bcl2l1	ENSRNOG00000007946	-0,735921	0.015855949	0.114051564
Cd48	ENSRNOG00000004737	-0,735537	0.001360246	0.02173603
Pde3b	ENSRNOG00000011417	-0,735517	0.010092667	0.084950705
Ccdc134	ENSRNOG00000007262	-0,735300	0.025352277	0.150300026
Slc66a3	ENSRNOG00000005126	-0,734869	0.033312924	0.176415825
Cd34	ENSRNOG00000045558	-0,733674	0.017313849	0.119358306
Nkain1	ENSRNOG00000011445	-0,733489	0.009883839	0.084009932
Hspg2	ENSRNOG00000021437	-0,733454	0.000410189	0.008839206
Nbeal1	ENSRNOG00000021525	-0,733389	0.005169104	0.055059209
Gng11	ENSRNOG00000050469	-0,733041	0.025786038	0.152061497
Pttg1ip	ENSRNOG00000001223	-0,732705	0.00164061	0.025034963
Tmod3	ENSRNOG00000032436	-0,732656	0.000331721	0.007479318
Fbn1	ENSRNOG00000007302	-0,732130	0.004624625	0.051064246
Tns1	ENSRNOG00000014182	-0,730694	0.00021721	0.005315026
Vps54	ENSRNOG00000007125	-0,728774	0.007086717	0.067861739
Gpx1	ENSRNOG00000048812	-0,728300	0.046024162	0.208390148
Gm2a	ENSRNOG00000052219	-0,727968	0.010613813	0.087912392
Cdc14a	ENSRNOG00000014515	-0,726955	0.020606589	0.133131501
Scrn1	ENSRNOG00000009636	-0,726189	0.014305441	0.106786393
Trrap	ENSRNOG00000025244	-0,724745	0.01332209	0.102396727
Serpinb9	ENSRNOG00000033772	-0,724362	0.022491658	0.140168015
Pyroxd2	ENSRNOG00000015807	-0,722883	0.026405823	0.154662679
Slc36a4	ENSRNOG00000011455	-0,722645	0.002262995	0.030873437
Akap12	ENSRNOG00000019549	-0,722580	4.96E-05	0.00168016
Gas7	ENSRNOG00000049361	-0,721899	5.03E-05	0.001691108
Myo6	ENSRNOG00000011852	-0,720059	0.017873075	0.121915876
Zfpm2	ENSRNOG00000004109	-0,719973	0.03373022	0.177060927
Fmo2	ENSRNOG00000003510	-0,719907	0.00670247	0.065224534
Lrrk1	ENSRNOG00000012730	-0,719510	0.020626069	0.133176193
Ppfibp2	ENSRNOG00000019667	-0,719428	0.044354368	0.204450014
Entpd1	ENSRNOG00000014574	-0,719353	0.003521256	0.042561026
Trim44	ENSRNOG00000005191	-0,718735	0.000347121	0.007736984
Tnfrsf1a	ENSRNOG00000031312	-0,718679	0.000464304	0.009749135
Lpin2	ENSRNOG00000014876	-0,717567	0.017880474	0.121915876
Setd4	ENSRNOG00000001699	-0,717447	0.035240584	0.181415434
Btg1	ENSRNOG00000004284	-0,717118	0.005522282	0.057621307
Glb1l	ENSRNOG00000019243	-0,716920	0.003948622	0.04615186
Esyt1	ENSRNOG00000060753	-0,716480	0.000630911	0.012256355
Lama5	ENSRNOG00000053691	-0,716425	0.008010941	0.073765051
Rab8b	ENSRNOG00000018009	-0,716260	0.009632878	0.082806943
Terf1	ENSRNOG00000007291	-0,715869	0.008989808	0.079354789
Pde8a	ENSRNOG00000019005	-0,714725	0.016404778	0.116017449
Ccnl2	ENSRNOG00000018691	-0,714716	0.022163196	0.138843019
Tspan14	ENSRNOG00000010813	-0,714435	0.000135498	0.00375633
Golga1	ENSRNOG00000014493	-0,714195	0.032957579	0.175453711
Vps8	ENSRNOG00000001764	-0,713390	0.001854404	0.027128274
Arsb	ENSRNOG00000011150	-0,712775	0.009213696	0.080464903
Tenm3	ENSRNOG00000012802	-0,712563	0.019676499	0.130131458

Dock6	ENSRNOG00000010652	-0,712432	0.031763553	0.171237424
Xrn2	ENSRNOG00000011785	-0,712235	0.003205786	0.039797723
Orc2	ENSRNOG00000012558	-0,711700	0.009273152	0.080848187
Tent2	ENSRNOG00000012099	-0,710628	0.003082069	0.038665036
Atp8b2	ENSRNOG00000020822	-0,710472	0.012145104	0.09594381
Zfp322a	ENSRNOG00000017318	-0,710207	0.010650496	0.088010182
Akt3	ENSRNOG00000021497	-0,710041	0.000172427	0.004484835
Prelid1	ENSRNOG00000016410	-0,709718	0.001630831	0.024959086
Crlf3	ENSRNOG00000050657	-0,709469	0.001357845	0.021719936
Pcdh18	ENSRNOG00000009949	-0,709242	0.002987105	0.037844417
Ehbp1l1	ENSRNOG00000056038	-0,708971	0.003585679	0.043105618
Wipf1	ENSRNOG00000018406	-0,708920	9.29E-05	0.002755938
Fgd4	ENSRNOG00000059491	-0,708909	0.001968854	0.0283204
Plscr4	ENSRNOG00000008151	-0,708502	0.000777617	0.014320657
Clmp	ENSRNOG00000053239	-0,707738	0.007993514	0.073735667
Pard3b	ENSRNOG00000024345	-0,707267	0.005383094	0.05647717
As3mt	ENSRNOG00000020081	-0,707109	0.040824909	0.196691674
Rabgap1l	ENSRNOG00000002736	-0,706681	0.005263604	0.055597963
Cdkl3	ENSRNOG00000059485	-0,706648	0.045217247	0.205978747
Pspc1	ENSRNOG00000020782	-0,705790	0.018227978	0.1235822
Slf2	ENSRNOG00000014482	-0,705525	0.005130285	0.054746471
Tgfbi	ENSRNOG00000012216	-0,704796	0.002288121	0.03110726
Prtfdc1	ENSRNOG00000024874	-0,704364	0.004056185	0.04691564
Glis2	ENSRNOG00000004766	-0,703897	0.006396201	0.063675914
Rab11fip5	ENSRNOG00000056009	-0,703641	0.002058336	0.029055663
Sipa1	ENSRNOG00000020726	-0,703341	0.03394961	0.177912858
Pde1a	ENSRNOG00000054212	-0,702118	0.040949621	0.196912069
Rpph1	ENSRNOG00000054493	-0,700750	0.012864087	0.099712675
Pdia3	ENSRNOG00000015018	-0,700273	0.001101437	0.018571844
C2H1orf54	ENSRNOG00000059750	-0,700184	0.031565309	0.170737524
Nfkbia	ENSRNOG00000007390	-0,700165	0.019837403	0.130391307
Srsf7	ENSRNOG00000027360	-0,699860	0.02810154	0.160190108
Hps1	ENSRNOG00000045838	-0,699499	0.037212795	0.186746151
Dpysl2	ENSRNOG00000009625	-0,699329	0.002021158	0.028810284
Atp2b4	ENSRNOG00000003031	-0,699252	0.000980142	0.017119523
Shisa5	ENSRNOG00000020667	-0,699219	0.015799794	0.113910592
Zfp354c	ENSRNOG00000029205	-0,697703	0.016973052	0.118106368
Itpr2	ENSRNOG00000001804	-0,696225	0.032413442	0.173599663
Prr13	ENSRNOG00000042094	-0,696034	0.010713063	0.088291753
Elmo2	ENSRNOG00000018747	-0,695820	0.005782831	0.059391234
Mtss1	ENSRNOG00000009001	-0,695607	0.005740707	0.059114481
Gnl3	ENSRNOG00000028461	-0,694841	0.016604071	0.116487167
Cdc73	ENSRNOG00000003258	-0,694334	0.008165991	0.074575693
Gmfb	ENSRNOG00000064480	-0,694219	0.005982931	0.060924224
Ptprd	ENSRNOG00000005711	-0,693983	0.002240228	0.030744173
Ctsa	ENSRNOG00000015857	-0,693816	0.001087946	0.018404126
Picalm	ENSRNOG00000018322	-0,693717	0.010019997	0.084688782
Tmem176a	ENSRNOG00000023708	-0,693346	0.002270942	0.030954742
Tgfbr2	ENSRNOG00000013265	-0,693288	0.000132572	0.003688353
Fnbp1l	ENSRNOG00000013798	-0,692655	0.013777134	0.104299196

Eif1a	ENSRNOG00000031421	-0,692388	0.009389812	0.081500431
Mcm9	ENSRNOG00000003281	-0,692299	0.047994616	0.212744575
Rnasel	ENSRNOG00000027017	-0,691946	0.033916824	0.177860695
Nfat5	ENSRNOG00000011879	-0,691194	0.001974305	0.0283204
Cttnbp2	ENSRNOG00000061845	-0,689933	0.04402941	0.204001135
Actn4	ENSRNOG00000020433	-0,689265	0.008300668	0.075224296
Dgcr8	ENSRNOG00000001886	-0,688794	0.008581981	0.076710995
Tnks1bp1	ENSRNOG00000009195	-0,688630	0.001930613	0.027983486
Thoc2	ENSRNOG00000007315	-0,688532	0.020755277	0.133733342
Chn1	ENSRNOG00000017939	-0,687868	0.003255008	0.040248429
Smg1	ENSRNOG00000047386	-0,687295	0.015281161	0.111917525
Cd276	ENSRNOG00000033608	-0,686611	0.001705821	0.025653171
Clk4	ENSRNOG00000003714	-0,685334	0.013527879	0.103067274
Stn1	ENSRNOG00000020376	-0,685290	0.035125747	0.181018762
Etl4	ENSRNOG00000008666	-0,684794	0.018270678	0.123763986
Pgm2	ENSRNOG00000002185	-0,684674	0.005594555	0.057992791
Dgkq	ENSRNOG00000024112	-0,684517	0.029337785	0.164595856
Dennd6a	ENSRNOG00000011636	-0,684056	0.003481998	0.042184706
Mospd2	ENSRNOG00000003350	-0,683701	0.048431478	0.21381897
Cyp4v3	ENSRNOG00000042426	-0,682500	0.003571225	0.042998214
Naa16	ENSRNOG00000045863	-0,682436	0.022410653	0.139830986
Srsf6	ENSRNOG00000006380	-0,681849	0.017163108	0.118739438
Gen1	ENSRNOG00000004667	-0,681490	0.029692313	0.165553927
Man1c1	ENSRNOG00000017087	-0,680846	0.015973986	0.114372565
Rbm39	ENSRNOG00000019848	-0,680614	0.003831528	0.045121089
Cers5	ENSRNOG00000052990	-0,680584	0.002891185	0.036982486
Taok1	ENSRNOG00000015692	-0,680503	0.016288938	0.115539975
Uvrag	ENSRNOG00000016206	-0,679639	0.001842479	0.027029966
Pgam1	ENSRNOG00000050585	-0,679237	0.007997882	0.073735667
Pwwp2a	ENSRNOG00000003936	-0,678816	0.013770044	0.104296201
Stk3	ENSRNOG00000011278	-0,677324	0.011237121	0.090947712
Rhob	ENSRNOG00000021403	-0,677286	0.00722099	0.068643667
Ptpn13	ENSRNOG00000002061	-0,676744	0.021630063	0.136491043
Ttc23	ENSRNOG00000013877	-0,676202	0.002060034	0.029055663
S100pbp	ENSRNOG00000007099	-0,676065	0.000161052	0.004256979
Dnm2	ENSRNOG00000007649	-0,675516	0.01571419	0.113567565
Actr3	ENSRNOG00000003206	-0,675336	0.003821039	0.045031613
Ift122	ENSRNOG00000010952	-0,675093	0.009088679	0.07973064
Klf12	ENSRNOG00000009145	-0,674707	0.006499119	0.064248909
U2surp	ENSRNOG00000008607	-0,674129	0.011456382	0.092243113
Bmp2k	ENSRNOG00000002040	-0,674013	0.002763295	0.035787317
Ankrd42	ENSRNOG00000009664	-0,672999	0.024293634	0.14647632
Atrx	ENSRNOG00000056703	-0,672570	0.005270499	0.055597963
Rnpc3	ENSRNOG00000017310	-0,672358	0.015592324	0.113253339
Lrrcc1	ENSRNOG00000010891	-0,672061	0.013930416	0.105153045
Foxp1	ENSRNOG00000009184	-0,671710	0.015139385	0.1113127
Fam91a1	ENSRNOG00000008271	-0,671498	0.001177315	0.019575851
Ier2	ENSRNOG00000067274	-0,671056	0.013417329	0.102662939
Golim4	ENSRNOG00000024213	-0,670874	0.000622288	0.012149432
Agap1	ENSRNOG00000019476	-0,670822	0.002368703	0.031951862

Krit1	ENSRNOG00000007937	-0,670765	0.020890943	0.134274296
Git2	ENSRNOG00000001190	-0,670462	0.022903679	0.141884421
Mib1	ENSRNOG00000013281	-0,669871	0.004423624	0.049582777
Sacm1l	ENSRNOG00000005149	-0,669591	0.001921359	0.02792423
Fam172a	ENSRNOG00000013844	-0,669452	0.001267424	0.020763903
Arhgef40	ENSRNOG00000052354	-0,669364	0.001589405	0.024474203
Tead3	ENSRNOG00000000506	-0,669015	0.049044052	0.215544804
Psme4	ENSRNOG00000060340	-0,668734	0.017375341	0.119405545
Myo1d	ENSRNOG00000003276	-0,668690	0.012959665	0.100254012
Slk	ENSRNOG00000011339	-0,668441	0.008426144	0.075896644
Bbs7	ENSRNOG00000015816	-0,668039	0.018492443	0.12501656
Man2b1	ENSRNOG00000023910	-0,667980	0.010099652	0.084963595
Eif2ak3	ENSRNOG00000006069	-0,667377	0.002184299	0.030196427
B3galnt1	ENSRNOG00000012019	-0,667117	0.003358521	0.041233855
Xpo6	ENSRNOG00000016722	-0,666693	0.046621329	0.209748859
Itgb1	ENSRNOG00000010966	-0,666668	0.012508999	0.097836452
Map3k2	ENSRNOG00000014089	-0,665857	0.03116002	0.169530672
Tnfrsf1b	ENSRNOG00000016575	-0,665741	0.000642511	0.012435176
Septin2	ENSRNOG00000017952	-0,665708	0.018164784	0.123207374
Lrrc8c	ENSRNOG00000065000	-0,665525	0.012546824	0.098083053
Slc20a1	ENSRNOG00000018567	-0,665352	0.000612632	0.012040824
Snx16	ENSRNOG00000009953	-0,665307	0.008303327	0.075224296
Scaper	ENSRNOG00000006864	-0,664874	0.021494872	0.136025567
Snx1	ENSRNOG00000017029	-0,664578	0.000145076	0.00396533
Tinagl1	ENSRNOG00000013179	-0,663350	0.004674586	0.0515064
Spaca6	ENSRNOG00000039076	-0,662726	0.022589784	0.140554646
Shld2	ENSRNOG00000059057	-0,662642	0.006950981	0.066890849
Rab27a	ENSRNOG00000052499	-0,661998	0.032761898	0.174924734
Ascc3	ENSRNOG00000037604	-0,661498	0.014119879	0.106222946
Map4k4	ENSRNOG00000014013	-0,661468	0.001461485	0.022976723
Tspan5	ENSRNOG00000015913	-0,660528	0.026820652	0.155641845
Ifnar2	ENSRNOG00000045668	-0,660029	0.001694608	0.025533847
Tp53bp1	ENSRNOG00000013837	-0,659851	0.00125215	0.020535262
Plekha5	ENSRNOG00000008747	-0,659791	0.047594018	0.212070624
Idua	ENSRNOG00000000043	-0,659622	0.016456029	0.116116366
Azin1	ENSRNOG00000005333	-0,659347	0.016653654	0.11671792
Septin11	ENSRNOG00000002182	-0,659099	0.000119374	0.003375394
Ppfibp1	ENSRNOG00000031709	-0,658293	0.000502448	0.010313746
Nhlrc3	ENSRNOG00000070230	-0,658193	0.009337107	0.081249062
Hsf2	ENSRNOG00000000808	-0,656902	0.003794726	0.044789263
Hace1	ENSRNOG00000000327	-0,656742	0.00033912	0.007602141
Nek1	ENSRNOG00000010418	-0,655752	0.036904391	0.185653992
Exoc6b	ENSRNOG00000059615	-0,655506	0.017730417	0.121054765
Mink1	ENSRNOG00000033508	-0,655397	0.043883257	0.203725012
Txlna	ENSRNOG00000048242	-0,655200	0.019296934	0.128536225
Herc4	ENSRNOG00000061040	-0,655133	0.010295121	0.085862435
Haus7	ENSRNOG00000055648	-0,655128	0.019767195	0.130318069
Ip6k2	ENSRNOG00000020361	-0,655078	0.003072826	0.038665036
Fbh1	ENSRNOG00000018549	-0,654579	0.01576053	0.113732773
Csrp1	ENSRNOG00000008937	-0,653504	0.001003146	0.017327198

Syne3	ENSRNOG00000005287	-0,652480	0.017151135	0.118709323
Dennd4a	ENSRNOG00000011739	-0,652404	0.009428325	0.081663221
Cul7	ENSRNOG00000017857	-0,651848	0.004055583	0.04691564
Jam2	ENSRNOG00000042848	-0,650752	0.032101037	0.172638644
Epb41l5	ENSRNOG00000002538	-0,650373	0.019455903	0.129373869
Arid4b	ENSRNOG00000016391	-0,647743	0.010881937	0.089182821
Nod1	ENSRNOG00000010629	-0,646887	0.002835603	0.036451063
Tsc22d3	ENSRNOG00000056135	-0,646461	0.012818345	0.099596477
Soga1	ENSRNOG00000053240	-0,645199	0.00095928	0.016811672
Glt8d1	ENSRNOG00000018179	-0,644708	0.036859321	0.185653992
Ppp4r1	ENSRNOG00000013733	-0,644433	0.000483443	0.010016007
Cap1	ENSRNOG00000013492	-0,644310	0.009083764	0.07973064
Itgb6	ENSRNOG00000008346	-0,644174	0.00602457	0.061148402
Slc12a7	ENSRNOG00000016372	-0,643482	0.014100686	0.106129805
Ckb	ENSRNOG00000010872	-0,642758	0.001967351	0.0283204
Dlc1	ENSRNOG00000010780	-0,642212	0.004736873	0.051899077
Pdgfrb	ENSRNOG00000018461	-0,641766	0.003966776	0.046259262
Ddx60	ENSRNOG00000059097	-0,640540	0.018039253	0.122676369
Lmbr1l	ENSRNOG00000061607	-0,639351	0.017075531	0.118472217
Tbce	ENSRNOG00000029667	-0,638869	0.01844246	0.124819081
Luzp1	ENSRNOG00000022402	-0,638092	0.000808135	0.014726022
Snx6	ENSRNOG00000005249	-0,637879	0.000782948	0.014401807
Luc7l3	ENSRNOG00000002835	-0,637737	0.005413567	0.056758664
Adamtsl5	ENSRNOG00000033787	-0,637018	0.007426129	0.06974026
Ripor2	ENSRNOG00000018804	-0,636815	0.02022755	0.131859928
Adarb1	ENSRNOG00000001227	-0,636411	0.008251782	0.075051231
Ralgds	ENSRNOG00000010219	-0,636287	0.030511703	0.167648975
Gabpb2	ENSRNOG00000021105	-0,635075	0.00456556	0.050663409
Ppib	ENSRNOG00000016781	-0,634977	0.002802684	0.036147194
Apbb3	ENSRNOG00000017849	-0,634830	0.041866718	0.198685187
Hsd17b11	ENSRNOG00000002210	-0,634277	0.001977832	0.028322265
Aida	ENSRNOG00000058805	-0,633502	0.005999319	0.06097155
Vcl	ENSRNOG00000010765	-0,633213	0.0090492	0.079608435
Inafm1	ENSRNOG00000048848	-0,633182	0.012989709	0.10043656
Cmip	ENSRNOG00000013178	-0,632833	0.047281681	0.211088481
Adcy2	ENSRNOG00000032150	-0,632505	0.004681602	0.051512755
Igfbp7	ENSRNOG00000002050	-0,632212	0.021783195	0.137077168
Slc29a2	ENSRNOG00000020025	-0,631950	0.014354195	0.106954028
N4bp2	ENSRNOG00000027152	-0,631813	0.000102475	0.0029898
Ikbip	ENSRNOG00000008247	-0,631747	0.005665046	0.058528796
Usp33	ENSRNOG00000011294	-0,631639	0.030948558	0.169031608
Pdcd11	ENSRNOG00000020304	-0,631604	0.03670196	0.185474062
Cbl	ENSRNOG00000008444	-0,631393	0.005270744	0.055597963
Rbbp8	ENSRNOG00000012899	-0,630227	0.008078985	0.074128729
Ankfy1	ENSRNOG00000016212	-0,629985	0.002049212	0.028997931
Cdkn1c	ENSRNOG00000059500	-0,629951	0.043062304	0.201613578
Trmt13	ENSRNOG00000015140	-0,629815	0.046235146	0.208778427
Ptpn9	ENSRNOG00000017600	-0,629498	0.000763468	0.014160515
Dstn	ENSRNOG00000005924	-0,628798	0.009429534	0.081663221
Lasp1	ENSRNOG00000004132	-0,628435	0.000890017	0.015893919

Suco	ENSRNOG00000026542	-0,627086	0.007274854	0.068859192
Man1b1	ENSRNOG00000012559	-0,626766	0.005061423	0.054382245
Dcaf12	ENSRNOG00000050790	-0,626240	0.000358176	0.007937949
Otud5	ENSRNOG00000008764	-0,626008	0.00228247	0.031057538
Pla1a	ENSRNOG00000057153	-0,625986	0.021091913	0.134898195
Egf	ENSRNOG00000053979	-0,625874	0.043676639	0.203228931
Slco2b1	ENSRNOG00000017976	-0,625643	0.003962009	0.046238277
Nadk	ENSRNOG00000016936	-0,625578	0.003638125	0.043434469
Tcf12	ENSRNOG00000057754	-0,624068	0.003108861	0.038873232
Tprkb	ENSRNOG00000050645	-0,623798	0.01404042	0.105727278
Castor1	ENSRNOG00000006740	-0,623243	0.030132857	0.166596846
Smc1a	ENSRNOG00000003139	-0,622092	0.009232945	0.080542711
Tnfaip8l2	ENSRNOG00000021100	-0,621792	0.014510182	0.107805738
Casp8ap2	ENSRNOG00000006487	-0,621278	0.011594301	0.09306502
Pibf1	ENSRNOG00000009208	-0,620940	0.036634144	0.18525153
Rassf9	ENSRNOG00000038574	-0,620221	0.028305679	0.160891089
Smap2	ENSRNOG00000011421	-0,619952	0.001635331	0.024986289
Sorbs1	ENSRNOG00000015658	-0,619244	0.002346267	0.031704113
Lmna	ENSRNOG00000019638	-0,618836	0.011843687	0.094271116
Ly6c	ENSRNOG00000061813	-0,618610	0.041674437	0.19842182
Fzd6	ENSRNOG00000004660	-0,618165	0.016421329	0.116029163
Tlnrd1	ENSRNOG00000012349	-0,617958	0.007158418	0.068338326
Zfp267	ENSRNOG00000042283	-0,617871	0.033576041	0.176838333
C2h4orf3	ENSRNOG00000014654	-0,617303	0.045127932	0.205883799
Ankrd12	ENSRNOG00000012733	-0,616787	0.014576459	0.108150374
Ahi1	ENSRNOG00000013969	-0,616684	0.027957716	0.159846315
Tfpi	ENSRNOG00000005039	-0,616608	0.003114475	0.038891097
Osbpl8	ENSRNOG00000026962	-0,615900	0.002759338	0.035783923
Pum3	ENSRNOG00000012574	-0,615893	0.040626223	0.196154298
Mipol1	ENSRNOG00000009151	-0,615851	0.032347119	0.173423302
Slc35f5	ENSRNOG00000003403	-0,615346	0.023329575	0.143651291
Chd6	ENSRNOG00000016744	-0,615066	0.018568629	0.125195144
Relch	ENSRNOG00000015508	-0,613223	0.004997488	0.053845685
Gxylt1	ENSRNOG00000005234	-0,612504	0.000380159	0.008342076
Csnk1g1	ENSRNOG00000016620	-0,612335	0.020591474	0.133131501
Grk6	ENSRNOG00000014615	-0,611925	0.013678533	0.103906165
Rngtt	ENSRNOG00000007867	-0,610903	0.017944428	0.122256084
Agfg1	ENSRNOG00000015619	-0,610470	0.002959536	0.037624685
Rab4b	ENSRNOG00000001500	-0,610415	0.041242324	0.197343617
Ranbp1	ENSRNOG00000001884	-0,608964	0.008022178	0.073824883
Adam15	ENSRNOG00000020590	-0,608045	0.004514711	0.050337407
Stard13	ENSRNOG00000001090	-0,607579	0.0085125	0.076221119
Sntb2	ENSRNOG00000020344	-0,607464	0.004616513	0.051010834
Tbc1d15	ENSRNOG00000003889	-0,607396	0.02564751	0.151473923
Uhrf2	ENSRNOG00000011308	-0,607299	0.006150075	0.062219593
Cnp	ENSRNOG00000017496	-0,606962	0.016211373	0.115330226
Sec23b	ENSRNOG00000008411	-0,606304	0.025139151	0.149720174
Pck2	ENSRNOG00000018536	-0,605639	0.021518129	0.136060248
Crebzf	ENSRNOG00000018987	-0,605591	0.002385778	0.032098812
Nrros	ENSRNOG00000001752	-0,604371	0.006193454	0.062374932

Sri	ENSRNOG00000049780	-0,604241	0.001431344	0.022616984
Ythdc1	ENSRNOG00000001996	-0,604227	0.009331262	0.081249062
Tspan9	ENSRNOG00000051620	-0,603840	0.002094296	0.029383527
Gramd2b	ENSRNOG00000015225	-0,602645	0.012307051	0.096742612
Rbm5	ENSRNOG00000018153	-0,602442	0.028513435	0.161424169
Pdlim4	ENSRNOG00000050794	-0,601456	0.029746848	0.165565884
Ntn4	ENSRNOG00000005573	-0,601079	0.047081355	0.210681663
Ddx17	ENSRNOG00000051170	-0,600756	0.017659176	0.120723988
Cd302	ENSRNOG00000006623	-0,600195	0.04130654	0.197380187
Rapgef6	ENSRNOG00000009995	-0,599988	0.047460717	0.211691373
Cyb5r3	ENSRNOG00000009592	-0,599968	0.02078111	0.133844435
Pcf11	ENSRNOG00000009891	-0,599675	0.039877681	0.19426514
Gucy1a1	ENSRNOG00000012302	-0,599636	0.006218729	0.062467951
Dzip3	ENSRNOG00000001956	-0,599566	0.037477056	0.187445437
Ttpal	ENSRNOG00000009076	-0,599515	0.025068825	0.149415569
Fkbp9	ENSRNOG00000005478	-0,599107	0.026289961	0.154215961
Lats1	ENSRNOG00000014916	-0,599096	0.007897897	0.073243588
Phf6	ENSRNOG00000002276	-0,598575	0.027822619	0.159458131
Htra1	ENSRNOG00000020533	-0,598207	0.024011319	0.145567199
Srrm2	ENSRNOG00000058561	-0,598040	0.041362103	0.197380187
Caskin2	ENSRNOG00000004310	-0,597587	0.000439095	0.009358541
Tax1bp3	ENSRNOG00000019357	-0,597408	0.026802976	0.155641845
Ift80	ENSRNOG00000009808	-0,597303	0.011159477	0.090648933
Slf1	ENSRNOG00000040279	-0,596401	0.014935619	0.110178477
Supt20h	ENSRNOG00000059480	-0,595905	0.029534062	0.165163151
Glmn	ENSRNOG00000002054	-0,595880	0.037739933	0.188364201
Tbc1d12	ENSRNOG00000036598	-0,595876	0.013925263	0.105153045
Slc4a2	ENSRNOG00000014347	-0,595579	0.009074203	0.07973064
Gpr137b	ENSRNOG00000002480	-0,595514	0.022548811	0.140468
Daam1	ENSRNOG00000004345	-0,595229	0.002680402	0.035093
Bltp3b	ENSRNOG00000050317	-0,592925	0.037381728	0.187247415
Skic3	ENSRNOG00000040297	-0,592693	0.023732107	0.144698054
Spats2l	ENSRNOG00000016012	-0,592253	0.012553709	0.098087654
Usp20	ENSRNOG00000007710	-0,592219	0.004207935	0.048064247
Tshz2	ENSRNOG00000048433	-0,591990	0.029662896	0.165553927
Reep3	ENSRNOG00000000645	-0,591532	0.010978503	0.089693277
Atp11a	ENSRNOG00000017154	-0,591339	0.010040385	0.084739544
Nop58	ENSRNOG00000016486	-0,591168	0.038968738	0.191713916
Cdk5rap2	ENSRNOG00000005788	-0,590264	0.012207427	0.096299604
Arfip1	ENSRNOG00000010533	-0,590131	0.026052374	0.153211541
Npdc1	ENSRNOG00000013102	-0,590082	0.001724948	0.025841052
Emilin1	ENSRNOG00000008246	-0,590029	0.01998116	0.130801039
S1pr1	ENSRNOG00000013683	-0,589808	0.004454928	0.049861913
Gtf2h2	ENSRNOG00000018230	-0,589689	0.031737928	0.171226848
Acvrl1	ENSRNOG00000028713	-0,589506	0.008501471	0.076166137
Slc6a6	ENSRNOG00000009019	-0,589231	0.009773035	0.083523796
Gnb1	ENSRNOG00000016638	-0,589166	0.032071008	0.172536709
Dido1	ENSRNOG00000009936	-0,588984	0.033518914	0.176785605
Apaf1	ENSRNOG00000008022	-0,588831	0.002445863	0.0325697
Dock7	ENSRNOG00000008298	-0,587951	0.011668748	0.093497945

Arhgap1	ENSRNOG00000016610	-0,587478	0.013525272	0.103067274
Zfp748	ENSRNOG00000069921	-0,587164	0.03593727	0.18288132
Zfp207	ENSRNOG00000000236	-0,586878	0.047530172	0.211940493
Tnfaip2	ENSRNOG00000010165	-0,586815	0.020062308	0.13114232
Impact	ENSRNOG00000045844	-0,586745	0.036856188	0.185653992
Cdk17	ENSRNOG00000004148	-0,586744	0.019820472	0.130391307
Shprh	ENSRNOG00000014450	-0,585983	0.03436722	0.179377315
Tbc1d5	ENSRNOG00000010637	-0,585884	0.035331445	0.18167126
Mindy3	ENSRNOG00000016955	-0,585301	0.017384437	0.119405545
Zdhhc20	ENSRNOG00000011024	-0,585227	0.019964774	0.130759427
Tagln2	ENSRNOG00000008301	-0,584763	0.020085122	0.131205954
Itpripl2	ENSRNOG00000069633	-0,584296	0.026881805	0.15591396
Asap2	ENSRNOG00000061519	-0,584290	0.008473073	0.076042901
App	ENSRNOG00000006997	-0,584238	0.018811406	0.126165177
Fam43a	ENSRNOG00000001728	-0,584144	0.004343003	0.048996365
Eps15	ENSRNOG00000010299	-0,583220	0.009934142	0.084207801
Saa4	ENSRNOG00000055531	-0,581565	0.013408605	0.102662939
Hnrnpa3	ENSRNOG00000052968	-0,580997	0.000859644	0.01546565
Loxl2	ENSRNOG00000016758	-0,580822	0.005552658	0.057827814
Tut7	ENSRNOG00000016629	-0,580806	0.041487913	0.197730708
Map3k7	ENSRNOG00000005724	-0,580740	0.005432375	0.05691755
Rem1	ENSRNOG00000007567	-0,580001	0.016804194	0.117245563
Ctnnd1	ENSRNOG00000030790	-0,579284	0.002527475	0.0334563
Tcerg1	ENSRNOG00000018849	-0,578578	0.006725257	0.065405429
Atr	ENSRNOG00000010027	-0,578192	0.030295856	0.167014747
Fam76b	ENSRNOG00000024863	-0,578121	0.003311397	0.040796826
Snapc3	ENSRNOG00000010825	-0,577771	0.032234312	0.173120008
Prkch	ENSRNOG00000004873	-0,577482	0.015659871	0.113459644
Esam	ENSRNOG00000033217	-0,577371	0.01580909	0.113924894
Cstf2	ENSRNOG00000048025	-0,577107	0.031396068	0.170317108
Cep295	ENSRNOG00000010999	-0,577100	0.003285114	0.040524204
Ywhaq	ENSRNOG00000051650	-0,577064	0.017241048	0.118939018
Usp48	ENSRNOG00000013602	-0,576820	0.02276636	0.141202185
Atp2b1	ENSRNOG00000004026	-0,576153	0.007483974	0.070098805
Myl6	ENSRNOG00000054140	-0,575581	0.008349153	0.075452325
Arhgef3	ENSRNOG00000014363	-0,574976	0.011743477	0.093923705
Pde3a	ENSRNOG00000025042	-0,574216	0.026755557	0.155541632
Ppt1	ENSRNOG00000012616	-0,573269	0.042553257	0.200517654
Cd46	ENSRNOG00000008193	-0,571499	0.013666411	0.103864721
Kdm3a	ENSRNOG00000007814	-0,570651	0.041751394	0.198540586
Hnrnpu	ENSRNOG00000033790	-0,570146	0.018820289	0.126165663
Taok2	ENSRNOG00000019964	-0,569524	0.044351462	0.204450014
Gpatch8	ENSRNOG00000021044	-0,569169	0.035510843	0.182087617
Marf1	ENSRNOG00000056248	-0,568855	0.017023283	0.118350182
Cd63	ENSRNOG00000007650	-0,567671	0.011847423	0.094271116
Cdon	ENSRNOG00000011789	-0,567257	0.017078857	0.118472217
Cd274	ENSRNOG00000016112	-0,567211	0.044198948	0.204142982
Slc4a7	ENSRNOG00000005957	-0,566510	0.020924343	0.134378094
Atp9b	ENSRNOG00000032039	-0,566037	0.047996802	0.212744575
Erp29	ENSRNOG00000001348	-0,564527	0.021960644	0.138073782

Mgat4a	ENSRNOG00000018276	-0,563946	0.009659149	0.082960052
Setx	ENSRNOG00000013491	-0,563691	0.015823831	0.113928817
Rlim	ENSRNOG00000045695	-0,563178	0.032657428	0.174546391
Usp40	ENSRNOG00000018395	-0,563070	0.044239572	0.204222972
Cstf3	ENSRNOG00000012089	-0,560912	0.041232648	0.197343617
Myo1e	ENSRNOG00000061928	-0,560499	0.04152153	0.19783041
Efemp2	ENSRNOG00000020587	-0,560446	0.033549231	0.176825784
Add3	ENSRNOG00000012820	-0,560173	0.011183582	0.090777931
Bmal1	ENSRNOG00000014448	-0,560100	0.048028854	0.212744575
Med13	ENSRNOG00000003679	-0,559921	0.01028149	0.085801821
Arhgap29	ENSRNOG00000012563	-0,559702	0.016390647	0.115970152
Snx33	ENSRNOG00000017382	-0,559038	0.007084275	0.067861739
Capn2	ENSRNOG00000034015	-0,558459	0.00523438	0.055401928
Dmtf1	ENSRNOG00000005908	-0,557874	0.031018054	0.169090722
Nek6	ENSRNOG00000010897	-0,557318	0.036528041	0.184834973
Nagk	ENSRNOG00000013911	-0,557068	0.035433115	0.181953836
Nipbl	ENSRNOG00000056907	-0,556766	0.024988379	0.1492604
Niban1	ENSRNOG00000002403	-0,556696	0.006295156	0.063032474
Tagln	ENSRNOG00000017628	-0,556371	0.037389391	0.187247415
Ino80	ENSRNOG00000014483	-0,556118	0.046244824	0.208778427
Pygb	ENSRNOG00000007583	-0,556115	0.046938051	0.210681663
Smad1	ENSRNOG00000018483	-0,555567	0.009851941	0.083876091
Tspan4	ENSRNOG00000029810	-0,555361	0.045228068	0.205978747
Coro1c	ENSRNOG00000000697	-0,555115	0.010852513	0.089131338
Ahnak	ENSRNOG00000057569	-0,554327	0.025909952	0.152676647
Sh3pxd2a	ENSRNOG00000020353	-0,554127	0.010153453	0.085232113
Jak2	ENSRNOG00000059968	-0,553582	0.024897324	0.149249828
Btaf1	ENSRNOG00000017938	-0,552099	0.046421922	0.209274754
Il6st	ENSRNOG00000013963	-0,550892	0.02111586	0.13499594
Cdh13	ENSRNOG00000014371	-0,550822	0.030257186	0.166869718
Slc25a36	ENSRNOG00000013802	-0,548344	0.040863577	0.196691674
Acta2	ENSRNOG00000058039	-0,548263	0.043902234	0.203752399
Kdm5a	ENSRNOG00000010591	-0,548230	0.007486804	0.070098805
Wdfy3	ENSRNOG00000061121	-0,547736	0.015372153	0.112282298
Cpsf7	ENSRNOG00000020668	-0,547035	0.014496495	0.107805738
Plxnb2	ENSRNOG00000007133	-0,546929	0.000644249	0.012453354
Abi1	ENSRNOG00000031325	-0,546797	0.032853861	0.175235591
Vwa1	ENSRNOG00000018338	-0,545927	0.012238931	0.096401694
Rab3gap2	ENSRNOG00000002353	-0,544025	0.018077469	0.122829032
Angptl4	ENSRNOG00000007545	-0,542522	0.049912612	0.218192621
Arf3	ENSRNOG00000054775	-0,541375	0.020196184	0.131820923
Dis3	ENSRNOG00000009125	-0,540948	0.023994219	0.145567199
Cflar	ENSRNOG00000012473	-0,539857	0.020821342	0.133992777
Mcc	ENSRNOG00000011271	-0,539816	0.040620016	0.196154298
Zyx	ENSRNOG00000017354	-0,539724	0.000450518	0.00952384
Anxa4	ENSRNOG00000018159	-0,539248	0.0089075	0.078938089
Kidins220	ENSRNOG00000023176	-0,538735	0.047592956	0.212070624
Fgd6	ENSRNOG00000054515	-0,538195	0.025307445	0.150117367
Sp3	ENSRNOG00000060479	-0,537312	0.015704799	0.113567565
Pikfyve	ENSRNOG00000015158	-0,537088	0.015486886	0.112794526

Gnaq	ENSRNOG00000014183	-0,536976	0.026822744	0.155641845
Prr14l	ENSRNOG00000048891	-0,536799	0.018146382	0.123136166
Ptprg	ENSRNOG00000009419	-0,536753	0.003596836	0.043116056
Tra2a	ENSRNOG00000009156	-0,536313	0.039399861	0.192973853
H6pd	ENSRNOG00000017523	-0,535301	0.010637389	0.087983603
Dcaf17	ENSRNOG00000009403	-0,534483	0.023392354	0.143676548
Eif4ebp3	ENSRNOG00000030247	-0,534293	0.036953074	0.185808548
Hps3	ENSRNOG00000031406	-0,533909	0.038388067	0.190230943
Capza1	ENSRNOG00000013538	-0,533069	0.032927306	0.175453483
Tbc1d32	ENSRNOG00000000802	-0,532709	0.018704749	0.125557943
Hmgcr	ENSRNOG00000016122	-0,532642	0.006933882	0.066808836
H1f5	ENSRNOG00000060355	-0,532507	0.040612631	0.196154298
Map1b	ENSRNOG00000017428	-0,531961	0.035010565	0.180988087
Zfp451	ENSRNOG00000012718	-0,531532	0.01592962	0.114286323
Htt	ENSRNOG00000011073	-0,530642	0.022047778	0.138398219
Ctdspl2	ENSRNOG00000022141	-0,530440	0.032454053	0.173757438
Dtna	ENSRNOG00000016671	-0,529256	0.040700643	0.196320749
Lrp4	ENSRNOG00000015285	-0,529241	0.028985012	0.163145408
Ano6	ENSRNOG00000006995	-0,529115	0.008135026	0.074336483
Tcf7l2	ENSRNOG00000049232	-0,528967	0.0233733	0.143651291
Prkd3	ENSRNOG00000005289	-0,528909	0.029088726	0.163551912
Tet2	ENSRNOG00000023579	-0,528780	0.010887402	0.089182821
Rif1	ENSRNOG00000054901	-0,527785	0.016014066	0.114501677
Frrs1	ENSRNOG00000016351	-0,527673	0.022138301	0.138761684
Eeig2	ENSRNOG00000027540	-0,527640	0.046881054	0.210552556
Ptbp1	ENSRNOG00000010448	-0,527332	0.038481954	0.190407711
Arid4a	ENSRNOG00000026239	-0,527125	0.039775948	0.194083707
Tspan8	ENSRNOG00000004411	-0,526974	0.043113429	0.201774475
Rin2	ENSRNOG00000010131	-0,526833	0.019518146	0.129677066
Rab30	ENSRNOG00000010224	-0,526544	0.044580101	0.204618632
Cpsf6	ENSRNOG00000005927	-0,524850	0.019278418	0.128467816
Top2b	ENSRNOG00000048795	-0,524549	0.009514801	0.082218853
Parp4	ENSRNOG00000061152	-0,524202	0.034016698	0.178144592
Clk3	ENSRNOG00000030126	-0,523897	0.042364683	0.200194649
Uba6	ENSRNOG00000021931	-0,523581	0.038902872	0.191624012
Lamc1	ENSRNOG00000002680	-0,522068	0.017437609	0.119629214
Ap3b1	ENSRNOG00000010624	-0,521781	0.012471389	0.097640321
Ets1	ENSRNOG00000008941	-0,520709	0.021307825	0.135560951
Kcnab1	ENSRNOG00000056697	-0,520389	0.02622602	0.153898829
Phip	ENSRNOG00000008652	-0,520373	0.026075869	0.153248599
Ptk2	ENSRNOG00000007916	-0,520190	0.039662403	0.1938332
Gtf3c2	ENSRNOG00000056904	-0,520166	0.031042744	0.169166126
Cog3	ENSRNOG00000000155	-0,519279	0.046119881	0.208639007
Rbms2	ENSRNOG00000003076	-0,518441	0.03506439	0.181018762
Glb1	ENSRNOG00000010196	-0,518411	0.015176145	0.111425229
Ubtd2	ENSRNOG00000004137	-0,518139	0.039742536	0.194049786
Prickle2	ENSRNOG00000012364	-0,517733	0.026171241	0.153693153
Plce1	ENSRNOG00000014276	-0,517165	0.025271531	0.150049714
Pear1	ENSRNOG00000014243	-0,516891	0.018262376	0.12376156
Amt	ENSRNOG00000050214	-0,515529	0.033650297	0.176910609

Pbrm1	ENSRNOG00000028227	-0,514643	0.023785816	0.14490393
Trpm7	ENSRNOG00000057806	-0,513956	0.042869819	0.201178246
Zzef1	ENSRNOG00000024535	-0,513871	0.034504639	0.179793404
Pan3	ENSRNOG00000000942	-0,513239	0.015057298	0.110866114
Ankrd13a	ENSRNOG00000001204	-0,511640	0.001510964	0.023470411
Ap4e1	ENSRNOG00000022938	-0,511063	0.031750601	0.171226848
Ldlrad4	ENSRNOG00000016879	-0,510966	0.038448439	0.190407711
Gak	ENSRNOG00000000048	-0,509901	0.024622674	0.148116316
Tbc1d2b	ENSRNOG00000014543	-0,508397	0.020066963	0.13114232
Mast2	ENSRNOG00000058476	-0,508295	0.021155041	0.135029336
Crlf1	ENSRNOG00000020030	-0,507946	0.040909311	0.196778964
Prrc2c	ENSRNOG00000068743	-0,507413	0.0239539	0.145441061
Wasf2	ENSRNOG00000009805	-0,507046	0.002553071	0.033652153
Phtf1	ENSRNOG00000019785	-0,506986	0.025435948	0.150566894
Camsap2	ENSRNOG00000008741	-0,503675	0.003487852	0.042222793
Fmr1	ENSRNOG00000057464	-0,502867	0.027173366	0.157091298
Adam9	ENSRNOG00000017231	-0,501099	0.018649188	0.125414757
Nt5e	ENSRNOG00000011071	-0,500969	0.023233004	0.143354536
Zfp347	ENSRNOG00000071023	-0,500674	0.016756403	0.117121919
Frmd8	ENSRNOG00000020881	-0,500029	0.007409141	0.069664701
Ralgps2	ENSRNOG00000004736	-0,499650	0.02935178	0.16459597
Lrp6	ENSRNOG00000006338	-0,499209	0.006443897	0.063865082
Sgpl1	ENSRNOG00000000565	-0,499194	0.013463267	0.102822398
Adss	ENSRNOG00000004481	-0,498893	0.033673343	0.176910609
Snd1	ENSRNOG00000031173	-0,498696	0.026572166	0.154996014
Alms1	ENSRNOG00000022343	-0,498110	0.032711616	0.174776054
Zkscan1	ENSRNOG00000001335	-0,498043	0.011921134	0.094616033
Rbpms	ENSRNOG00000013328	-0,496498	0.029031047	0.163345507
Cdc42bpa	ENSRNOG00000002841	-0,494833	0.037434959	0.187355176
Eif4a1	ENSRNOG00000030628	-0,493478	0.032820914	0.175119809
Uggt1	ENSRNOG00000014901	-0,492067	0.016167988	0.115126716
Mtfr2	ENSRNOG00000058050	-0,491776	0.024797086	0.148820725
Gpihbp1	ENSRNOG00000064680	-0,491560	0.033235451	0.176176909
Tmem43	ENSRNOG00000007519	-0,490372	0.037963642	0.188788233
Coro1b	ENSRNOG00000021828	-0,490005	0.026889654	0.15591396
Ncbp3	ENSRNOG00000018550	-0,488037	0.031513398	0.170597201
Jag1	ENSRNOG00000007443	-0,487793	0.035119386	0.181018762
Lrig3	ENSRNOG00000007654	-0,487690	0.026393692	0.154649764
Arhgap12	ENSRNOG00000017791	-0,487568	0.025591756	0.151259316
Tmem68	ENSRNOG00000008872	-0,487342	0.040597596	0.196154298
Dpp7	ENSRNOG00000012640	-0,486647	0.027784983	0.159326477
Heg1	ENSRNOG00000001793	-0,486500	0.009986099	0.084556204
Nsd2	ENSRNOG00000038140	-0,486342	0.030412536	0.167411751
Tmem260	ENSRNOG00000013353	-0,486101	0.026057633	0.153211541
Agrn	ENSRNOG00000020205	-0,484331	0.047627414	0.212070624
Rcn2	ENSRNOG00000015780	-0,483669	0.027872605	0.15959397
Osbpl5	ENSRNOG00000020713	-0,483156	0.029170678	0.163894398
C1h9orf85	ENSRNOG00000027161	-0,482856	0.034939473	0.180849499
Pcdh19	ENSRNOG00000003929	-0,481803	0.01227847	0.096566661
Rab23	ENSRNOG00000012629	-0,481618	0.023495934	0.144007339

Adam17	ENSRNOG00000060694	-0,481363	0.026302191	0.154229634
Zfta	ENSRNOG00000032042	-0,480923	0.014220523	0.106568425
Smurf2	ENSRNOG00000014623	-0,480560	0.028682987	0.162207238
Ankrd28	ENSRNOG00000019697	-0,479918	0.048237341	0.213323239
Elovl1	ENSRNOG00000028448	-0,477696	0.019840812	0.130391307
Rc3h1	ENSRNOG00000002750	-0,475287	0.040530394	0.196045807
Notch3	ENSRNOG00000004346	-0,474594	0.037777043	0.188401514
Mical3	ENSRNOG00000053203	-0,474269	0.016346506	0.11571039
Wwtr1	ENSRNOG00000016617	-0,473073	0.042790367	0.2010487
Pggt1b	ENSRNOG00000003541	-0,472460	0.031499249	0.170579876
Prkci	ENSRNOG00000009652	-0,472192	0.030509777	0.167648975
Mafb	ENSRNOG00000016037	-0,471692	0.009502816	0.082160864
Ganab	ENSRNOG00000019724	-0,471161	0.034642885	0.180152255
Srsf1	ENSRNOG00000050323	-0,470875	0.041236549	0.197343617
Trmt11	ENSRNOG00000014240	-0,469197	0.048799449	0.215015672
Pms2	ENSRNOG00000001040	-0,469114	0.03368034	0.176910609
Gnai2	ENSRNOG00000016592	-0,468876	0.032962324	0.175453711
Dcbld2	ENSRNOG00000055281	-0,467278	0.048193122	0.2131882
Sec31a	ENSRNOG00000002251	-0,466029	0.003678675	0.043784384
Crip2	ENSRNOG00000005041	-0,465221	0.035966063	0.182882267
Arrdc4	ENSRNOG00000010775	-0,464961	0.012739022	0.09908835
Camta2	ENSRNOG00000004283	-0,464551	0.028371482	0.161153912
Evi5	ENSRNOG00000002039	-0,464281	0.032936799	0.175453483
Uba5	ENSRNOG00000011027	-0,464127	0.012499712	0.097812911
Mga	ENSRNOG00000006378	-0,464037	0.029901836	0.165967439
Vps37c	ENSRNOG00000046439	-0,463249	0.00307627	0.038665036
Lamb2	ENSRNOG00000047768	-0,462466	0.042016162	0.199091183
Atp6v0a1	ENSRNOG00000036814	-0,462313	0.04258266	0.200517654
Impdh1	ENSRNOG00000020032	-0,459618	0.01959793	0.129819618
Zfp131	ENSRNOG00000015925	-0,458362	0.024172583	0.145972421
Sestd1	ENSRNOG00000012603	-0,458148	0.001905995	0.02775271
Rubcn	ENSRNOG00000059880	-0,457448	0.017378321	0.119405545
Ggta1	ENSRNOG00000019179	-0,457230	0.033436358	0.176649188
Brwd1	ENSRNOG00000001632	-0,456253	0.047010257	0.210681663
Fermt2	ENSRNOG00000009102	-0,453994	0.037839384	0.188648678
Supt16h	ENSRNOG00000011953	-0,453396	0.022682229	0.140960961
Ptpn1	ENSRNOG00000010574	-0,453222	0.040454196	0.195859656
Sec14l1	ENSRNOG00000002722	-0,452894	0.009417502	0.081663221
Vps26c	ENSRNOG00000001681	-0,452669	0.048036094	0.212744575
Bmf	ENSRNOG00000007529	-0,451442	0.040418836	0.195856293
Tbc1d20	ENSRNOG00000005766	-0,451410	0.014761042	0.109115553
Setd5	ENSRNOG00000007472	-0,451226	0.015157203	0.111391142
Mcl1	ENSRNOG00000063678	-0,449197	0.047453884	0.211691373
Myo1c	ENSRNOG00000004072	-0,447711	0.041036785	0.197087888
Ggnbp2	ENSRNOG00000027860	-0,446619	0.029675223	0.165553927
Sec61a1	ENSRNOG00000013743	-0,446477	0.045264964	0.206026334
Myl9	ENSRNOG00000020246	-0,445970	0.021482347	0.136025567
Vps13c	ENSRNOG00000030213	-0,445940	0.031487293	0.170574417
Dcps	ENSRNOG00000009850	-0,445285	0.043765633	0.203311687
Pxk	ENSRNOG00000008024	-0,445065	0.038857765	0.191462358

Klf7	ENSRNOG00000046242	-0,442129	0.034737792	0.180344818
Cramp1	ENSRNOG00000024635	-0,441183	0.021268843	0.135473661
Akap10	ENSRNOG00000002899	-0,438717	0.048642494	0.214506103
Rasal2	ENSRNOG00000004917	-0,437537	0.034621643	0.180101905
Arhgdia	ENSRNOG00000036688	-0,437129	0.044275177	0.204266287
Zbed6	ENSRNOG00000053210	-0,435342	0.048714311	0.214762014
Ets2	ENSRNOG00000001647	-0,434789	0.048038217	0.212744575
Arhgap35	ENSRNOG00000015852	-0,431337	0.041275574	0.197380187
Ctnna1	ENSRNOG00000005796	-0,429504	0.030985512	0.169031608
Esyt2	ENSRNOG00000032391	-0,429091	0.03409123	0.178414969
Vps41	ENSRNOG00000012940	-0,428729	0.017730871	0.121054765
Mtrex	ENSRNOG00000010125	-0,427917	0.042796036	0.2010487
Cyth3	ENSRNOG00000001065	-0,427489	0.021495183	0.136025567
Os9	ENSRNOG00000025570	-0,425109	0.008409229	0.075819325
Lcorl	ENSRNOG00000003787	-0,424424	0.049488536	0.217191939
Msl3	ENSRNOG00000004016	-0,423928	0.044082706	0.204042946
Stxbp1	ENSRNOG00000015420	-0,420452	0.038223117	0.189715248
Morc2	ENSRNOG00000019624	-0,418522	0.009695113	0.083158921
Cpt1a	ENSRNOG00000014254	-0,416241	0.040994823	0.196947067
Cep192	ENSRNOG00000042879	-0,416071	0.04005978	0.194753491
Rhot1	ENSRNOG00000004093	-0,416054	0.044383031	0.204490707
Nisch	ENSRNOG00000018823	-0,409688	0.041090977	0.197185549
Ephb4	ENSRNOG00000045952	-0,409480	0.011681855	0.093526873
Cep120	ENSRNOG00000017035	-0,408476	0.024921894	0.1492604
Tie1	ENSRNOG00000020173	-0,407902	0.049468258	0.217191939
Igdcc4	ENSRNOG00000033496	-0,407652	0.028277207	0.160846615
Dmxl2	ENSRNOG00000009170	-0,407109	0.035364696	0.181690679
Tep1	ENSRNOG00000051706	-0,406638	0.033597013	0.176838333
Zfp790	ENSRNOG00000029043	-0,406105	0.038157785	0.189451337
Itgav	ENSRNOG00000004912	-0,404503	0.045606483	0.207217557
Cerk	ENSRNOG00000017022	-0,404491	0.043972814	0.203897751
Dhdds	ENSRNOG00000014665	-0,402576	0.011944166	0.094750561
Itgb5	ENSRNOG00000001795	-0,401910	0.010490022	0.087165086
Stk38	ENSRNOG00000000519	-0,398934	0.047659467	0.212127047
Stk4	ENSRNOG00000013529	-0,393149	0.039642634	0.1938332
Gtf3c3	ENSRNOG00000002091	-0,393129	0.04725211	0.211088481
Zeb1	ENSRNOG00000017863	-0,388298	0.033534425	0.176807563
Nfkb2	ENSRNOG00000019311	-0,387871	0.046327591	0.208970429
Lgals8	ENSRNOG00000018046	-0,383513	0.044587798	0.204618632
Fam117b	ENSRNOG00000022066	-0,376554	0.046758651	0.210184589
Prrc2a	ENSRNOG00000000852	-0,369984	0.041693191	0.19842182
Mylk	ENSRNOG00000002215	-0,352940	0.009890371	0.084019622
Cttn	ENSRNOG00000050994	-0,350966	0.047088279	0.210681663
Phc2	ENSRNOG00000006004	-0,348209	0.041962996	0.198899748
